# Linkage and regional association analysis reveal two new tightly-linked major-QTLs for pod number and seed number per pod in rapeseed (*Brassica napus L.*)

**DOI:** 10.1038/srep14481

**Published:** 2015-10-05

**Authors:** Jiaqin Shi, Jiepeng Zhan, Yuhua Yang, Jiang Ye, Shunmou Huang, Ruiyuan Li, Xinfa Wang, Guihua Liu, Hanzhong Wang

**Affiliations:** 1 Key Laboratory of Oil Crop Biology of the Ministry of Agriculture, Oil Crops Research Institute of the Chinese Academy of Agricultural Sciences, Wuhan 430062, China; 2 Key Laboratory of Information and Computing Science of Guizhou Province, Guizhou Normal University, Guiyang 550001, China

## Abstract

To facilitate the pseudochromosomes assembly and gene cloning in rapeseed, we developed a reference genetic population/map (named BnaZNF_2_) from two sequenced cultivars, Zhongshuang11 and No.73290, those exhibit significant differences in many traits, particularly yield components. The BnaZNF_2_ genetic map exhibited perfect collinearity with the physical map of *B. napus*, indicating its high quality. Comparative mapping revealed several genomic rearrangements between *B. napus* and *B. rapa* or *B. oleracea*. A total of eight and 16 QTLs were identified for pod number and seed number per pod, respectively, and of which three and five QTLs are identical to previously identified ones, whereas the other five and 11 are novel. Two new major QTL respectively for pod number and seed number per pod, *qPN.A06-1* and *qSN.A06-1* (*R*^2 ^= 22.8% and 32.1%), were colocalised with opposite effects, and only *qPN.A06-1* was confirmed and narrowed by regional association analysis to 180 kb including only 33 annotated genes. Conditional QTL analysis and subsequent NILs test indicated that tight linkage, rather than pleiotropy, was the genetic causation of their colocalisation. Our study demonstrates potential of this reference genetic population/map for precise QTL mapping and as a base for positional gene cloning in rapeseed.

Rapeseed, *Brassica napus* L. (AACC genome, 2n = 38), is mainly cultivated for the production of edible oil and is the second largest oil crop globally, after soybean. Rapeseed originated from recent (≈0.01 million years) allopolyploidy between the ancestors of two diploid species, *B. rapa* (AA genome, 2n = 20) and *B. oleracea* (CC genome, 2n = 18)^1^. Yield is the most important target for the genetic improvement of most crops, including rapeseed. Yield is also the most complex trait in crops. Yield is multiply determined by its component traits^2^, each of which is quantitatively inherited and controlled by many quantitative trait loci (QTLs). In addition, negative correlations are usually observed among yield components, which has traditionally been considered the genetic trade-off among competitive demands, i.e., antagonistic pleiotropy^3^. Among the three yield components of rapeseed, pod number is most highly correlated with seed yield, followed by seed number per pod, while the correlation of seed weight with yield is relatively low^2^, suggesting that the pod number and seed number per pod are the major contributors to the yield in rapeseed.

Nearly ten linkage QTL mapping studies and one association analysis study involving pod number and/or seed number per pod in rapeseed have been reported^2^,^4^,^5^,^6^,^7^,^8^,^9^,^10^,^11^,^12^, but the genetic and molecular bases of both traits remain ambiguous. First, the linkage maps used in these studies involved markers with unknown sequence information, and the genomic sequence of rapeseed was unknown, preventing the integration of these QTLs in the reference physical map and the identification of the underlying genes. Second, the genomic sequences of the parental cultivars used in these studies were unknown, complicating the development of molecular markers and the screening of candidate genes. More importantly, only a few major QTLs for pod number and/or seed number per pod have been identified, and none have been fine-mapped or further cloned.

Recent innovations in genome sequencing technology and bioinformatics have enabled the sequencing of European winter type rapeseed cultivar Darmor-*bzh*^13^ and Chinese semi-winter type cultivar Zhongshuang11 (http://www.brassica.info/resource/sequencing.php) by our own and several other institutes. Approximately a dozen linkage maps have been developed using different genetic populations of rapeseed (http://www.brassica.info/resource/maps/published-data.php); however, the number of sequence-based markers in these linkage maps was insufficient, and none of these genetic populations were derived from Zhongshuang11. To accurately anchor and orientate the 2730 sequence scaffolds of Zhongshuang11 (http://www.ncbi.nlm.nih.gov/genome/genomes/203), a skeleton linkage map of sequence-based markers must be constructed using a reference population derived from itself. In addition, tens of rapeseed cultivars have also been resequenced (http://www.brassica.info/resource/sequencing.php)^14^. These sequenced cultivars may represent a unique genetic resource for the development of reference populations for genetic study in rapeseed.

Among the sequenced cultivars, Zhongshuang11 (*de novo* sequencing) and No.73290 (resequencing) exhibit significant differences in many traits related to agronomy, plant/root architecture and yield (particularly pod number and seed number per pod)^15^, suggesting that they are ideal parents for reference population construction and genetic study in rapeseed. The main objectives of the current study were the following: (1) construction of a skeleton linkage map with sequence-based polymerase chain reaction (PCR) markers using the reference population derived from Zhongshuang11 and No.73290; (2) validation of the quality of reference skeleton linkage map by comparative mapping with the physical maps of *B. napus* and *B. rapa*/*B. oleracea*; (3) mapping and comparison of QTLs for pod number and seed number per pod using the reference skeleton linkage map; (4) validation and fine mapping of the major QTLs by regional association analysis; and (5) dissection of the genetic basis of the colocalised QTLs for pod number and seed number per pod by conditional QTL analysis.

## Results

### Construction of a skeleton linkage map using the BnaZNF_2_ population

Of the 9351 screened primer pairs, i.e. 6504 simple sequence repeat (SSR), 2592 single nucleotide polymorphism (SNP), 179 sequence tagged site (STS) and 76 Insertion/Deletion (InDel), 961 (10.3%) primer pairs displayed clear polymorphism between the two parental cultivars Zhongshuang11 and No.73290, producing 1038 sequence-based PCR markers in the BnaZNF_2_ population (Supplementary Table S1).

**Figure 1:**
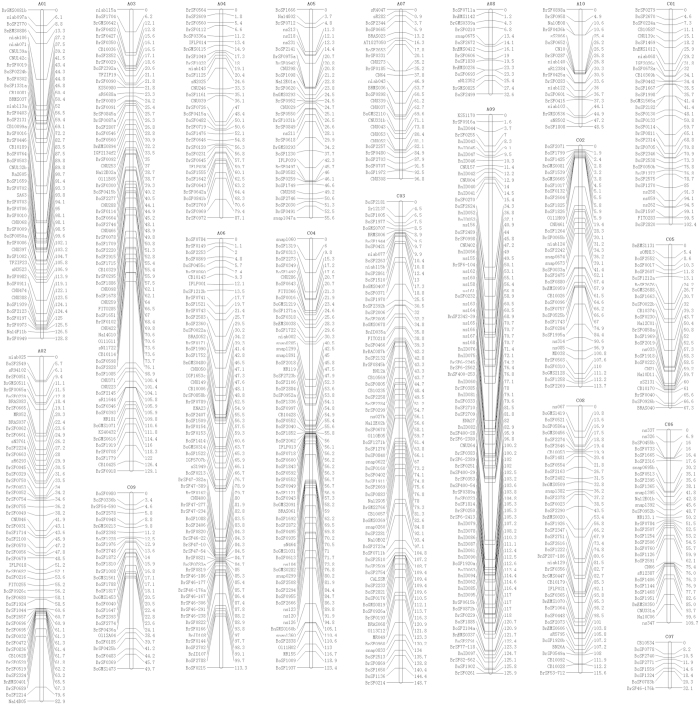
The BnaZNF_2_ genetic map of 19 linkage groups. The names of the assigned pseudochromosomes (A01–A10; C01–C09) are indicated at the top of each linkage group. The names of the 803 sequence-based PCR markers are provided on the left side of each linkage group. The genetic distances (cM) of these markers are indicated on the right side of each linkage group.

All 1038 markers were subjected to Joinmap 4.0 software for linkage analysis, which resulted in a linkage map of 19 linkage groups and 803 markers (710 SSR, 48 SNP, 31 InDel and 14 STS) (Fig. 1; Table 1). Of these, 499 SSR markers (prefixed by “BoSF”, “BrSF” and “BnSF”), 31 SNP (prefixed by “ns”) markers and all 31 InDel markers (prefixed by “BnID”) were newly developed and mapped (Supplementary Table S2). The 19 linkage groups were successfully assigned to the 19 chromosomes of the A (A01-A10) and C (C01–C09) genomes, respectively. The length of the 19 linkage groups ranged from 32.1 (C07) to 148.7 (C03) cM, with a sum and mean of 1763.2 and 92.8 cM, respectively. It should be noted that A05, A08, A10, C07 and C09 were typically shorter than the other 14 linkage groups. The marker density of the 19 linkage groups ranged from 4.01 (C07) to 1.45 (A09) cM, with an average of 2.19 cM.

Only 40 (5.0% of the total) markers exhibited significant (P ≤ 0.001) segregation distortion, of which 5 and 35 skewed to Zhongshuang11 and No.73290, respectively (Table 1). Interestingly, the distorted markers tended to be concentrated on/near the two ends of the linkage groups. The distorted markers on A06 bottom, A09 top and C02 bottom skewed to Zhongshuang11, whereas those on A01 top, A04 bottom and A09 bottom skewed to No.73290 (Supplementary Table S3).

### Validation of the quality of BnaZNF_2_ linkage map by comparative mapping with the physical maps of *B. napus* and *B. rapa*/*B. oleracea*

**Figure 2:**
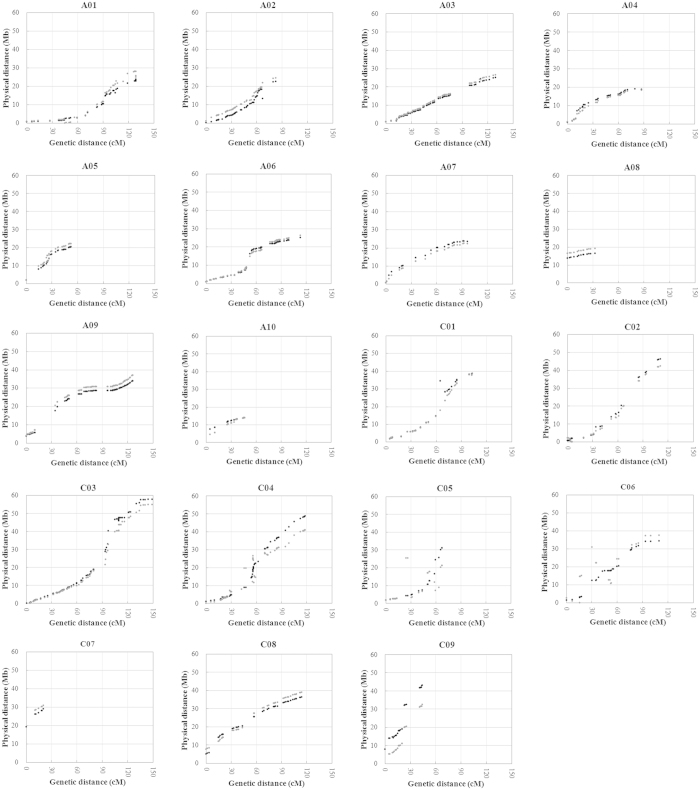
BnaZNF_2_ genetic map versus physical maps of *Brassica napus* and *B. rapa*/*B. oleracea*. The genetic and physical distances of the mapped markers are presented on the horizontal and vertical axes, respectively. The scatter dots indicate the genetic positions of the mapped markers on the linkage groups of BnaZNF_2_ genetic map and their physical positions on the pseudochromosomes (A01–A10; C01–C09) of *B. napus* (black) and *B. rapa*/*B. oleracea* (grey).

The availability of the pseudochromosomes of *B. napus*^13^, *B. rapa*^16^ and *B. oleracea*^17^ facilitated the comparison of genetic and physical maps in *Brassica*. Comparative mapping between the BnaZNF_2_ linkage map and the physical maps of *B. napus* and *B. rapa*/*B. oleracea* was based on the markers aligned to their pseudochromosomes, and the results are displayed using dot-plots (Fig. 2).

A total of 26, 17 and 733 markers were successfully aligned to the pseudochromosomes of *B. napus*, *B. rapa*/*B. oleracea* and both, respectively, while the remaining 27 failed (Supplementary Table S4). Nearly all markers mapped on each of the 19 linkage groups were collinear with the corresponding pseudochromosomes of *B. napus*. This collinearity was also supported by the high correlation between the genetic and physical positions of the aligned markers, with coefficients ranging from 0.884 (C05) to 0.997 (A03) for the different chromosomes (mean = 0.963) (Table 2). These results strongly indicate the high quality of the current BnaZNF_2_ linkage map. High collinearity was also observed between most linkage groups and the corresponding pseudochromosomes of *B. rapa*/*B. oleracea,* with the exception of C05 and C06. The discontinuous collinearity observed for C05 and C06 was also supported by the intermediate correlation between the genetic and physical positions of the aligned markers, with r = 0.586 and 0.640, respectively. A detailed comparison of the physical positions of these aligned markers revealed several obvious genomic rearrangements between the pseudochromosomes (including C05 and C06) of *B. napus* and *B. rapa*/*B. oleracea*: translocation in one location in A01, A02 and C01 and in two locations in C05 and C06; inversion in one location in C02 and C06; and reshuffling in A05 and C04 (Fig. 2; Supplementary Table S4).

### Phenotypic variation and correlation of pod number and seed number per pod in the parents and linkage population

**Figure 3:**
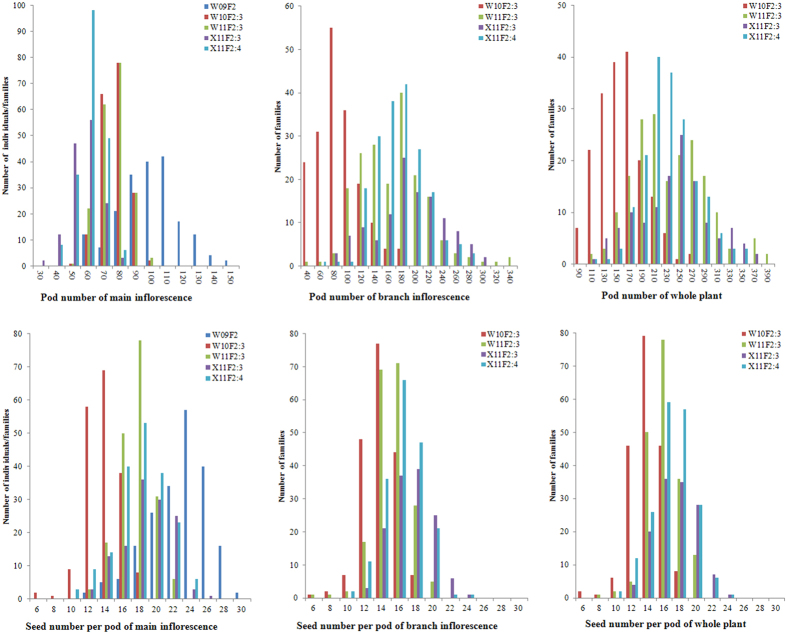
Distribution of pod number and seed number per pod in the BnaZNF_2_ and corresponding F_2:3_ and F_2:4_ populations planted in four environments. The horizontal axis represents the trait value of pod number and seed number per pod for the main inflorescence, branch inflorescence and whole plant. The vertical axis represents the number of individuals/families within the population. The different experiments (environment and population combination) are represented by different colours as indicated in the legend. W09F_2_, W10F_2:3_, W11F_2:3_, X11F_2:3_ and X11F_2:4_ were the codes of the five experiments which have been detailedly described in the first section of Methods.

The two parents (Zhongshuang11 and No.73290) differed significantly in pod number and seed number per pod in all four investigated environments (Table 3). The pod number of No.73290 was approximately 50% greater than that of Zhongshuang11, while the seed number per pod of Zhongshuang11 was nearly twice that of No.73290. Interestingly, the pod number of branch inflorescence was nearly twice that of main inflorescence in both the parents and populations. A normal or near-normal distribution was observed in the populations for both traits in all four investigated environments (Fig. 3), indicating a quantitative inheritance suitable for QTL identification. In addition, the populations exhibited transgressive segregation of both traits but with a small degree (Table 3), indicating that the favourable alleles were mainly distributed on one of the two parents.

The pod number of the whole plant was significantly positively correlated with those of the main and branch inflorescence with moderate and high coefficients, respectively (Table 4), suggesting that the pod number of the whole plant is mainly determined by that of the branch followed by the main inflorescence. The pod numbers of the main and branch inflorescence were also significantly positively correlated with moderate coefficient, suggesting that the genetic determination of the pod number of the main and branch inflorescence differs. The seed number per pod of the main and branch inflorescence were significantly positively correlated with high coefficient, indicating similar genetic control. As expected, the pod number and seed number per pod were significantly negatively correlated for the main and branch inflorescence as well as the whole plant, suggesting competition among sinks for assimilates. However, these correlation coefficients were all small, suggesting that the final seed yield of rapeseed (*B. napus* L.) could be improved by increasing its component traits, such as pod number and/or seed number per pod.

Analysis of variance (ANOVA) revealed that genotype, environment and the genotype × environment interaction all have significant effects on both the pod number and seed number per pod of the main and branch inflorescence as well as whole plant (Supplementary Table S5). The broad-sense heritability of the pod number was moderate as follows: main inflorescence (0.65)> whole plant (0.49)> branch inflorescence (0.45) (Table 4). Whereas, the broad-sense heritability of the seed number per pod was equally high for the main (0.83) and branch (0.82) inflorescence as well as whole plant (0.83).

### Linkage mapping and integration of the QTLs for pod number and seed number per pod

A total of 69 QTLs (51 and 18 at significant and suggestive levels, respectively) were detected for pod number and seed number per pod in five experiments (Supplementary Table S6A). After deleting seven non-reproducible suggestive QTLs, 16 and 46 QTLs were identified for pod number and seed number per pod, respectively, which were distributed on seven (A01, A03, A05, A06, A09, C02 and C06) and nine (A01-A03, A05-07, A09, C02 and C06) of the 19 linkage groups (Supplementary Table S6B). Where the confidence intervals of these identified QTLs in different experiments overlapped, they were integrated into a single consensus QTL for each trait (Supplementary Table S6C). Eight and 16 consensus QTLs were obtained for pod number and seed number per pod, respectively, and five and 13 of these were reproducible (Table 5).

**Figure 4:**
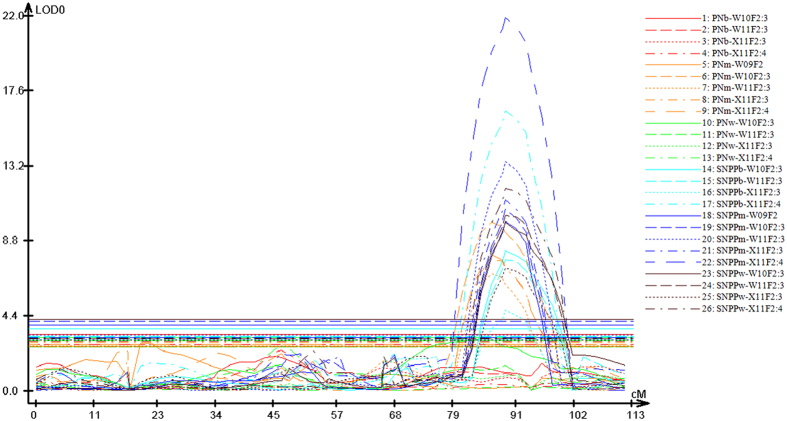
QTL scanning curves for the A06 linkage group for pod number and seed number per pod in five experiments. The horizontal and vertical axes, respectively, represent the genetic distance and LOD value. The lines and curves, respectively, indicate the threshold and true LOD values. The different traits and experiments are represented using different colours and (line/curve) styles, respectively, as indicated in the legend. PNm/PNb/PNw and SNPPm/SNPPb/SNPPw are the abbreviations of pod number and seed number per pod, respectively, for the main inflorescence/branch inflorescence/whole plant. W09F_2_, W10F_2:3_, W11F_2:3_, X11F_2:3_ and X11F_2:4_ were the codes of the five experiments.

Interestingly, the numbers of identified QTLs and consensus QTLs for seed number per pod were both much larger than those for pod number, consistent with the much higher heritability of seed number per pod compared to pod number (Table 4). In addition, the Zhongshuang11 alleles in the majority of both the identified and consensus QTLs decreased the pod number but increased the seed number per pod (Table 5; Supplementary Table S6C), consistent with the higher seed number per pod and lower pod number of Zhongshuang11 compared to No.73290 (Table 3). The consensus QTLs for pod number and seed number per pod explained 2.3–22.8% and 2.7–32.1% of the phenotypic variance, respectively. The additive/dominant effect of the consensus QTLs for pod number and seed number per pod ranged from −9.65/−7.48 to 7.29/9.17 and from −1.73/−1.05 to 1.97/1.59, respectively. Two consensus QTLs on the A06 linkage group (*qPN.A06-1* and *qSN.A06-1*) were repeatedly detected in four and all five experiments, respectively, and displayed a large effect (*R*^2 ^= 22.8% and 32.1%); thus, they can be treated as major QTLs (Fig. 4). Interestingly, the confidence intervals of three pairs of consensus QTLs for pod number and seed number per pod (*qPN.A05-1*/*qSN.A05-1*, *qPN.A06-1/qSN.A06-1* and *qPN.C02-1*/*qSN.C02-1*) overlapped well (Table 5). Of these, only *qPN.A06-1* and *qSN.A06-1* exhibited an opposite additive/dominant effect in the same experiments, explaining the moderate genetically negative correlation between pod number and seed number per pod (Table 4).

### Physical map-based comparison of currently and previously detected QTLs for pod number and seed number per pod in rapeseed

The availability of the pseudochromosomes of *B. napus*^13^ also enabled the physical map-based comparison of currently and previously detected QTLs for pod number and seed number per pod in rapeseed. The corresponding genomic intervals of these QTLs were determined by BLAST/e-PCR analysis using the associated markers (within confidence intervals) with available probe/primer sequences against the physical map of *B. napus*.

The genomic regions of the majority (≈80%) of these QTL could be determined, of which approximately a quarter overlapped (Table 6; Supplementary Table S7). In detail, *qPN.A03-1*, *qPN.A03-2*, *qPN.C06-1*, *qSN.A01-1*, *qSN.A09-1*, *qSN.C02-1*, *qSN.C06-1* and *qSN.C06-2* identified in the current study corresponded to *Sil/dm*^*2*^*_N3*, *qPN.A3-2*, *PNNP-C6*, *qSN.A1-1*, *qSN-LP1-A9a/qSN-OP2-A9*, *qSN.C2-1*, *qSN.C6-4* and *qSN.C6-1*, respectively, identified in the previous studies^2^,^5^,^8^,^9^. In addition, *PNLP-A3* and *qPN-LP2-A3b*, *PNNP-A5* and *qPN.A5-2*, *qPN.A6-1* and *PNLP-A6*, *Sil/dm2_N7* and *qPN.A7-1*, *NPP1_LG4* and *qPN.A7-2*, *qPN-LP1-A9* and *Sil/dm2_N9*, *qPN-LP3-C3a* and *qPN.C3-1*, *qSNA1* and *qSN.A1-7*, *qSN-OP3-A2/qSN-OP1-A2a/qSN-LP3-A2* and *SNLP-A2*, *qSN.A3-1* and *SNLP-A3*, *qSN.A5-1* and *qPN-OP1-A5*, *S/Sil_N5* and *qSN.A5-2*, *qSN.A9-1* and *SNNP-A9*, *qSN-OP1-C1* and *S/Sil_N11*, *qSN-OP3-C6b/qSN-LP1-C6* and *qSNC6*, *qSS.N19* and *qSN.C9-3*, which were identified in previous studies^2^,^4^,^5^,^9^,^11^,^12^, were also determined to be identical.

### Fine mapping of the major QTLs for pod number and seed number per pod by regional association analysis

To further fine-map the two colocalised major QTLs for pod number and seed number per pod, regional association analysis was conducted. The corresponding genomic region of the target major QTL was identified by the alignment of the primer-pairs sequences of the molecular markers within the QTL interval and reference genomic sequences of *B. napus*^13^ and *B. rapa*^16^. A total of 103 putative single-locus SSR markers (Supplementary Table S8) within the corresponding ~1.7 Mb genomic region of *B. rapa*^18^ were synthesised and used to screen polymorphism among several mini-core collection of the association population^15^. Of these, 35 SSR markers that amplified only one main band and exhibited obvious polymorphism were selected for further regional association analysis.

**Figure 5:**
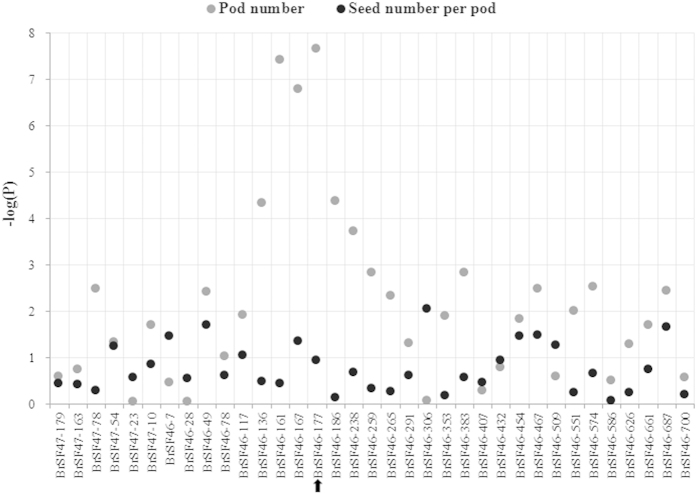
Manhattan plot for the association analysis of the target major QTL region for pod number and seed number per pod. The 35 single-locus SSR markers used for the association analysis are ordered on the horizontal axis according to their physical positions on the A06 pseudochromosome. The vertical axis shows the value of −log(P). The grey and black dots show the −log(P) values of the association of the 35 single-locus SSR markers with pod number and seed number per pod, respectively. The black arrow on the bottom indicates the peak signal.

**Figure 6:**
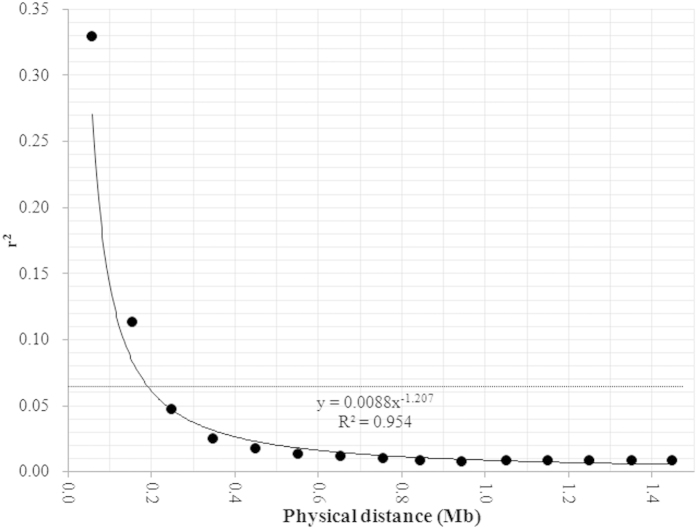
Trend of LD decay with increase in physical distance in the target major QTL region. The horizontal and vertical axes, respectively, represent the physical distance and the R^2^ of LD. The scatter plots show the mean R^2^ of the LD for each window. The dotted line indicates the background level of LD. The curve on the figure shows the trendline of the regression between physical distance and R^2^ of LD. The regression equation and its determination coefficient are also shown on the figure.

A large range of phenotypic variation was observed for both pod number (32–106) and seed number per pod (7.6–28.0) among the 576 inbred lines of the association population. Based on population structure and family relatedness, regional association analysis was conducted using mixed linear mode (MLM) by TASSEL3.0 software. Interestingly, a significant (P < 0.001) association signal was only observed for pod number and not for seed number per pod (Fig. 5). Of the six SSR markers associated with pod number, BrSF46–177 displayed the strongest association signal (P = 2.2E-08 and *R*^2 ^= 8.7%); this signal was very close (13 kb) to the peak marker (BrSF46–167) of *qPN.A06-1* identified by linkage analysis (Fig. 1; Table 5). Therefore, the confidence interval of *qPN.A06-1* was defined by genomic region between BrSF46-136 and BrSF46-238, which corresponds to 180 kb (from 22,607 to 22,787 kb) including only 33 annotated genes in the reference genome of *B. napus* (Table 7). Because the extent of linkage disequilibrium (LD) decay for the target QTL region was estimated to be 193 kb (Fig. 6), it was impossible to further fine-map it using the current association population. In addition, the –log(P) values of the association of the six SSR markers with pod number and seed number per pod were not accordant (Fig. 5). These results also suggested that the colocalisation of *qPN.A06-1* and *qSN.A06-1* was not likely caused by pleiotropy.

### Dissection of the genetic basis of the colocalised QTLs for pod number and seed number per pod by conditional QTL analysis

To determine the genetic basis (pleiotropy vs. tight-linkage) of the colocalisation of *qPN.A06-1* and *qSN.A06-1* in the same experiments, conditional QTL analysis was performed in both the linkage and association populations (Table 8). Regardless of whether pod number was conditioned by seed number per pod or seed number per pod was conditioned by pod number, the LOD values and *R*^2^ of the two major QTLs, as well as the –log(P) values and *R*^2^ of the six associated markers, exhibited only a small variation. These results suggest that tight linkage rather than pleiotropy is more likely the genetic basis of the colocalisation of *qPN.A06-1* and *qSN.A06-1*.

## Discussion

In the current study, an F_2_ population from two sequenced rapeseed cultivars was developed. Among the reported genetic populations in rapeseed^5^,^19^,^20^,^21^,^22^,^23^,^24^, this is the first for which parental genomic information is available, which will greatly facilitate subsequent studies. In addition, Zhongshuang11 and No.73290 exhibit large differences in many traits related to agronomy, plant/root architecture and yield^15^. More importantly, many QTLs with relatively large effects have been identified in this population, including for pod length, seed weight^15^, pod number, seed number per pod (Table 5) and flowering time (Shi *et al.* unpublished data). Due to the heterozygosity of the current BnaZNF_2_ population, the corresponding recombinant inbred lines population (named BnaZNRIL) was also developed in our lab. All major QTLs identified from the BnaZNF_2_ population have been fine-mapped in our laboratory, and the functional validation of the candidate genes is in progress. Therefore, the BnaZNF_2_/BnaZNRIL population is an ideal reference population for QTL mapping and map-based gene cloning in rapeseed.

To facilitate pseudochromosome assembly of Zhongshuang11, a skeleton linkage map of 803 sequence-based PCR markers was constructed using the BnaZNF_2_ population (Table 1). A total of 561 markers (499 SSR, 31 SNP and all 31 InDel) on the BnaZNF_2_ linkage map were developed in this study (Supplementary Table S2), increasing the number of sequence-based PCR markers in rapeseed^14^. To our knowledge, this is the first rapeseed linkage map comprising only sequence-based PCR markers. In addition, the current linkage map mainly (88.4%) comprises SSR markers and has more SSR markers (710) than other published linkage maps of *B. napus* (http://www.brassica.info/resource/maps/published-data.php). These favourable characteristics will facilitate the easy transfer of these markers to other genetic populations and the comparison/integration of different linkage maps in *B. napus* as well as in other *Brassica* species^25^,^26^.

The existence of short linkage groups is commonly observed in published linkage maps of *B. napus*^5^,^9^,^12^,^27^,^28^,^29^,^30^, but the detailed causation (insufficient number of markers, poor polymorphism of parents and low frequency of recombination) has not been investigated. Although a total of 9351 primer-pairs were used to screen the polymorphic markers for map construction (Supplementary Table S1), five short linkage groups were also identified in the current BnaZNF_2_ linkage map (Table 1). Of these, four linkage groups (A08, A10, C07 and C09; A05 was the exception) were nearly the same short length in a high-density genetic map constructed using the BnaZNRIL population and the *Brassica* 60K Illumina Infinium SNP array (Shi *et al.* unpublished data); therefore, they were unlikely to be caused by an insufficient number of markers. Because its genomic coverage ratio was not low, the short length of the C09 linkage group was obviously due to its low recombination frequency among all 19 linkage groups. For the A08, A10 and C07 linkage groups, their short length is likely due to the low polymorphism between parents because their genomic coverage ratios were very low, while the recombination frequencies were not low. In fact, the A08 and A10 linkage groups are also short in more than half of the published linkage maps of *B. napus*^5^,^9^,^12^,^27^,^28^,^29^,^31^. Thus, a lack of genetic diversity is likely a common characteristic of the two chromosomes in rapeseed, which may indicate a new direction for breaking the genetic bottleneck in rapeseed cultivars.

Interestingly, most of the distorted markers tended to cluster on both ends of the linkage groups and skew to the same parent (Supplementary Table S3). This clustering is not likely due to chance and suggests the presence of a segregation distortion region (SDR) in rapeseed, which can be explained by factors such as the selection of gametophytes and sporophytes, homeologous recombination and residual heterozygosity in parents^32^.

The major part of the constructed BnaZNF_2_ linkage map and physical map of *B. napus* were collinear (Fig. 2; Table 2), suggesting its high quality and suitability for rapeseed genomic studies such as pseudochromosome assembly and comparative mapping. The inconsistency between the genetic and physical distance of a few tightly-linked markers (Supplementary Table S4) may be due to genotyping errors, misanchored scaffolds or distorted markers. This is the first comparative study of the genetic and physical map of rapeseed. The BnaZNF_2_ skeleton linkage map-based comparison of the physical maps of *B. napus* and *B. rapa* or *B. oleracea* revealed several genomic rearrangements (including inversion, translocation and reshuffling) between these species. These rearrangements should occur very recently, after the formation of *B. napus* from the progenitors of the two diploid species *B. rapa* and *B. oleracea*. This finding is consistent with the reported genomic rearrangements in the resynthesised *Brassica* polyploids, including *B. napus*^33^,^34^,^35^,^36^.

In the reported linkage^2^,^4^,^5^,^6^,^7^,^8^,^9^,^11^,^12^ and association^10^ mapping studies of pod number and/or seed number per pod, more than 70 and 80 QTLs have been detected for pod number and seed number per pod, respectively, distributed on 16 (excluding A04, C04 and C07) and 17 (excluding C04 and C07) of the 19 linkage groups (Supplementary Table S7). Most of these QTLs exhibited a moderate effect, and only two on A02 (*qSNP2*) and C01 (*qSNP11b*) for pod number, three on A01 (*qSNA1*), C03 (*cqSS.N13*) and C09 (*qSS.C9*) for seed number per pod, and one on C01 (*qSNP11a*/*qSS11*) for both traits could be considered major QTLs^6^,^7^,^11^. In the current linkage and regional association analysis study, a total of eight and 16 QTLs were identified for pod number and seed number per pod, respectively, which were distributed on seven (A01, A03, A05, A06, A09, C02 and C06) and nine (A01-A03, A05-07, A09, C02 and C06) of the 19 linkage groups (Table 5). One and two thirds of them, respectively, exhibited a relatively large (*R*^2 ^≥ 10%) or moderate (*R*^2 ^< 10%) effect, and only two each for pod number and seed number per pod should be considered major QTLs. The recent completion of sequencing and assembling of rapeseed genome enabled the first physical map-based comparative QTL analysis in the current study, which undoubtedly increased the accuracy of comparison. Only three QTLs for pod number and five QTLs for seed number per pod identified in the current study had been reported previously (Table 6), whereas the remaining five and 11 are novel (Supplementary Table S7). In addition, seven pairs of QTLs for pod number and nine pairs of QTLs for seed number per pod, those had been reported in the previous studies^2^,^4^,^5^,^9^,^11^,^12^, are also identical. The aforementioned 24 pairs of QTLs common to the current and previous studies should be potential targets for marker-assisted selection to improve yield in rapeseed. More importantly, the major QTLs identified across these studies will be important targets of map-based gene cloning for elucidating the molecular basis of yield in rapeseed. These results demonstrate that both pod number and seed number per pod are controlled by a large number of loci that are distributed on nearly all of the 19 linkage groups and mostly exhibit moderate effects, which strongly suggests the complexity of the genetic architecture of both traits. This complexity is understandable because the pod number per plant and seed number per pod are affected by many biological/developmental processes, including inflorescence/flower/ovule differentiation, fertilization and pod/seed development^37^,^38^,^39^, each of which involves many genes^40^,^41^.

Several major QTLs for pod number and/or seed number per pod had been identified in previous QTL mapping studies^6^,^7^,^11^,^12^, but none have been fine-mapped and further cloned. The traditional/classical fine mapping strategy is based on screening of recombinant individuals from a large-scale segregation population of near-isogenic lines (NILs) developed from several rounds of successive backcrossing and/or self-crossing, which is time-consuming and labour intensive^42^. To directly fine-map the two colocalised major QTL (*qPN.A06-1* and *qSN.A06-1*) identified in the current study, a regional association strategy was proposed by using the high resolution of the natural population^15^. Using this strategy, *qPN.A06-1* was successfully narrowed to a much smaller (approximately 1/7) interval of 180 kb including only 33 annotated genes (Fig. 5). This result suggests that regional association analysis is an effective and highly efficient strategy for direct fine mapping of target major QTLs identified by preliminary linkage analysis. According to the detailed annotation of the 33 genes (Table 7), a homeodomain-like superfamily protein gene *GSBRNA2T00001826001* was likely to be the candidate^43^, because the two parents Zhongshuang11 and No.73290 showed significant difference in flowering time and flower organ number. To further confirm the candidate gene(s), comparative sequencing of the 180 kb genomic region between the two parents is in progress. However, *qSN.A06-1* was not validated by regional association analysis using the same association population and markers. This failure may have occurred because the genetic variation responsible for the major QTL of seed number per pod is rare in our association population or the expression of this major QTL depends on the genetic background^44^. These results suggest that the use of regional association analysis for fine mapping of the target major QTL depends on the type of variants (rare or common) and/or genetic background. These results also suggest that the fine mapping of *qSN.A06-1* have to use the traditional/classical NIL strategy.

Resource limitation force an organism to allocate energy to processes in a competitive manner^45^. In seed plants, the major trade-off between the number of fruits per inflorescence/plant and the number of seeds per fruit, which was traditionally explained by antagonistic pleiotropy, can be quantitated/reflected by the negative coefficients of genetic correlation^3^. For rapeseed, the negative correlation between pod number and seed number per pod has been commonly observed, including in the present study, and is consistent with the opposite additive-effect direction of most of the colocalised QTLs for both traits^2^,^6^,^9^,^46^. However, the genetic basis of these colocalised QTLs with the opposite effect for both traits has not previously been investigated and remains unknown. The existence of two colocalised major QTL with opposite direction for pod number and seed number per pod in the current study provided an ideal example to test the hypothesis of antagonistic pleiotropy. The genetic basis of their colocalisation was then investigated by further regional association analysis (Fig. 5) followed by conditional QTL analysis (Table 8). The results of both analyses suggested that their colocalisation was caused by tight-linkage rather than pleiotropy, which was also confirmed by the subsequent measurement of both traits on the NILs that were recombinant at the target major QTL region (Shi *et al.* unpublished data). This is the first study that provides solid evidences to clearly demonstrate a negative correlation between yield component traits in rapeseed as well as other crops. The existence of two tightly linked major QTLs for pod number and seed number per pod also indicates the retention of a large linkage drag during the breeding of the elite rapeseed cultivar Zhongshuang11. Therefore, the strategy described in this paper will be effective in increasing the pod number of Zhongshuang11 without decreasing the seed number per pod via the introgression of *qPN.A6* through backcross and marker-assisted selection^47^.

## Methods

### Population construction, field experiment and trait measurement

The reference genetic population (named BnaZNF_2_) used for the linkage analysis included 184 F_2_ individuals derived from Zhongshuang11 and No.73290. The natural population used for the association analysis was described in our previous study and comprises 576 global inbred lines, including the two parents of the linkage population^15^.

The F_2_ individuals were planted in Wuhan from Oct. 2008 to May 2009 (code W09F_2_). The F_2:3_ families were planted in Wuhan from Oct. 2009 to May 2010 (code W10F_2:3_) and Oct. 2010 to May 2011 (code W11F_2:3_) as well as in Xining from April to Aug. 2011 (code X11F_2:3_). The F_2:4_ families were planted in Xining from April to Aug. 2011 (code X11F_2:4_). The association population was planted in Wuhan from Oct. 2011 to May 2012 (code W12AP). The planting of the F_2:3_, F_2:4_ and association populations followed a randomised complete block design with two to three replications. Each block contained two rows, with 33.3 cm between rows and 16.7 cm between individual plants. The seeds were sown by hand, and the field management followed standard agricultural practice. In each block, 10 representative individuals from the middle of each row were harvested by hand at maturity.

Pod number was measured as the number of effective pods on the main inflorescence, branch inflorescence and whole plant, respectively (PNm, PNb and PNw). Seed number per pod was calculated as the number of well-filled seeds on the main inflorescence, branch inflorescence and whole plant, respectively, divided by the corresponding pod number (SNPPm, SNPPb and SNPPw).

### Molecular marker analysis and linkage map construction

Three groups of molecular markers were first used to screen polymorphism between the two parents of the linkage population, and the single-locus markers were then used to screen the mini-core collection of the association population. The first group, mainly consisting of SSR and STS markers, was downloaded from the public database of *Brassica* (http://www.brassica.info/resource/markers.php; http://brassica.nbi.ac.uk/IMSORB/). The second group, consisting of SSR markers (prefixed “BoSF”, “BrSF” and “BnSF”), was developed by our lab from the sequence scaffolds of *B. rapa*, *B. oleracea* and *B. napus*^18^. The third group consisted of SNP (prefixed “snap” and “ns”) and InDel (prefixed “BnID”) markers and was developed in our lab from the re-sequencing of several *B. oleracea* cultivars^48^ and No.73290^14^. For markers detected at more than one polymorphic locus, a lowercase alphabetic letter was added to distinguish the different loci. PCR, electrophoresis and silver staining were performed as previously described^18^.

The genetic linkage map was constructed using the software JoinMap 4.0 (http://www.kyazma.nl/index.php/mc.JoinMap) using a threshold for goodness-of-fit of ≤ 5, a recombination frequency of < 0.4 and minimum logarithm of odds (LOD) score of 2.0. All genetic distances were expressed in centimorgans (cM) as derived by the Kosambi function. Double-crossover events were examined, and the original scores were rechecked for potential scoring errors.

Linkage groups were assigned to the corresponding chromosomes (A01–A10; C01–C09) based on the common markers in the reported linkage maps of *B. napus* (http://www.brassica.info/CropStore/maps.php)^5^,^20^,^22^,^49^,^50^,^51^,^52^.

### QTL mapping and integration

QTL mapping was conducted using the composite interval mapping (CIM) procedure^53^ incorporated in WinQTLCart v2.5 software (http://statgen.ncsu.edu/qtlcart/WQTLCart.htm). The walk speed, number of control markers, window size and regression method were set to 1 cM, 5, 10 cM and forward regression, respectively. The default minimum distance between QTLs (5 cM) and minimum LOD from top to valley (1) were used to define a QTL peak in an experiment. The experiment-wise LOD threshold was determined by permutation analysis^54^ with 1000 repetitions. The LOD score corresponding to P = 0.05 (3.0–4.6) was used to identify significant QTLs. To avoid missing QTLs with a relatively small effect, a lower LOD score, corresponding to P = 0.10 (2.6–4.1), was adopted for the presence of suggestive QTLs. Both significant QTLs and reproducible suggestive QTLs were admitted^55^ and named as identified QTLs.

Because QTLs detected in different experiments and mapped to the same region of a chromosome might be several estimates of the position of a single QTL^56^, integration of these reproducible QTLs was conducted.

### Linkage disequilibrium and regional association analysis

Linkage disequilibrium (LD) was estimated as the correlation coefficient *R*^2^ between all pairs of markers using the standalone software TASSEL v3.0^57^. Rare alleles with a frequency of <0.05 were treated as missing data. The background level of LD was defined as the 95% quantile of the *R*^2^ value among unlinked markers^58^. The LD decay was calculated using the linked markers as previously described^58^.

The structure of the association population was inferred using the software STRUCTURE v2.3.4^59^. The iteration number and K-value (the putative number of genetic groups) were set to 3 and from 1 to 10, respectively. Both the length of the burnin period and number of MCMC (Markov Chain Monte Carlo) replications after burnin were set to 10000. The most likely K-value was determined by posterior probability [LnP(D)] and an *ad hoc* statistic Δk based on the rate of change in LnP(D) between successive k^60^.

The relative kinship of the 576 inbred lines of the association population was calculated using SPAGeDi v1.4 software^61^. All negative kinship values between individuals were set to zero.

The regional association analysis was conducted using single-locus SSR markers within the confidence interval of the target major QTL in the association population of 576 inbred lines. Based on the population structure and relative kinship (Q and K matrix), the calculation was performed with a mixed linear model (MLM) incorporated into the TASSEL v3.0 software. The threshold of the significant marker-trait association was set to P ≤ 0.001.

### Conditional QTL analysis

To determine the genetic basis of the colocalisation of QTLs for different traits^62^, conditional QTL analysis was performed using the conditional phenotypic values. The conditional phenotypic values y (T1|T2) were obtained by the mixed model approach for the conditional analysis of quantitative traits^63^ using QGAStation 1.0 (http://ibi.zju.edu.cn/software/qga/index.htm), where T1|T2 indicates that trait 1 is conditioned by trait 2.

### Statistical analysis

Broad-sense heritability was calculated as *h*^2 ^= σ_g_^2^/(σ_g_^2 ^+ σ_ge_^2^/n + σ_e_^2^/nr), where σ_g_^2^, σ_ge_^2^ and σ_e_^2^ are the variances of genotype, genotype by environment and error, respectively, while n and r are the number of environments and replicates, respectively. The genetic correlation was calculated as r_G _= cov_Gxy_/(σ_Gx_^2 ^× σ_Gy_^2^)^1/2^, where cov_Gxy_ is the genotypic covariance and σ_Gx_^2^ and σ_Gy_^2^ are the variances of the pairwise traits. The significance of each genetic correlation was determined using a t-test of the correlation coefficients^64^. The components of variance/covariance and the coefficients of correlation were estimated using the PROC GLM or CORR procedure, respectively, incorporated into SAS software version 8.1. The Excel statistical functions CHISQ.TEST and T.TEST were used to obtain the significance level (P_x_^2^_-test_ and P_t-test_) of the degree-of-fit and differences, respectively.

## Additional Information

**How to cite this article**: Shi, J. *et al.* Linkage and regional association analysis reveal two new tightly-linked major-QTLs for pod number and seed number per pod in rapeseed (*Brassica napus L.*). *Sci. Rep.* **5**, 14481; doi: 10.1038/srep14481 (2015).

## Supplementary Material

Supplementary Information

## Figures and Tables

**Table 1:** Summary statistics of the BnaZNF_2_ linkage map.

**Linkage group**	**Markers number**	**Genetic distance**	**Recombination frequency**^a^	**Marker density**^b^	**Coverage ratio (%)**^c^	**Number of marker skewed to**		
						**Zhongshuang11**	**No.73290**	**Total**
A01	49	128.8	5.6	2.63	98.2	0	5	5
A02	54	82.9	3.7	1.54	91.0	0	0	0
A03	72	129.1	5.3	1.79	81.7	0	0	0
A04	33	87.1	4.8	2.64	94.4	0	2	2
A05	33	55.6	4.0	1.68	61.1	0	0	0
A06	67	113.3	4.7	1.69	99.3	1	0	1
A07	27	96.8	4.2	3.59	96.7	0	0	0
A08	14	32.6	12.5	2.33	13.9	0	0	0
A09	87	125.9	4.2	1.45	88.5	2	28	30
A10	18	48.5	6.9	2.55	40.6	0	0	0
C01	34	102.4	2.7	3.01	99.5	1	0	1
C02	38	113.7	2.4	2.99	96.7	1	0	1
C03	75	148.7	2.6	1.98	95.4	0	0	0
C04	67	123.4	2.6	1.84	97.4	0	0	0
C05	26	67.3	2.3	2.59	68.2	0	0	0
C06	31	109.7	3.3	3.43	89.4	0	0	0
C07	8	32.1	3.3	4.01	21.5	0	0	0
C08	42	115.6	3.7	2.75	81.6	0	0	0
C09	28	49.7	1.4	1.78	72.7	0	0	0
Total	803	1763.2	3.4	2.19	80.8	5	35	40

^a^which is calculated as the genetic distance (cM) divided by physical distance (Mb).

^b^which is calculated as genetic distance (cM) divided by markers number.

^c^which is calculated as the covered physical distance (Mb) of linkage group divided by the length (Mb) of the corresponding pseudochromosome.

**Table 2:** Correlation coefficients between the genetic and physical positions of the aligned markers for each of the 19 linkage groups/pseudochromosomes of *B. napus* and *B. rapa*/*B. oleracea.*

**Linkage groups/Chromosomes**	**Correlation coefficients**	
	***B. napus***	***B. rapa*/*B. olercea***
A01	0.945	0.933
A02	0.964	0.982
A03	0.997	0.998
A04	0.942	0.971
A05	0.972	0.956
A06	0.955	0.971
A07	0.976	0.985
A08	0.986	0.990
A09	0.925	0.924
A10	0.981	0.989
C01	0.958	0.970
C02	0.967	0.963
C03	0.961	0.960
C04	0.982	0.968
C05	0.884	0.586
C06	0.985	0.640
C07	0.974	0.988
C08	0.983	0.993
C09	0.963	0.980
Mean	0.963	0.934

**Table 3:** Phenotypic variation of pod number and seed number per pod for the two parents and populations in four investigated environments.

**Environments**	**Materials**		**PNm**	**PNb**	**PNw**	**SNPPm**	**SNPPb**	**SNPPw**
W09	Parents	Zhongshuang11	76	/	/	24.1	/	/
		No.73290	124	/	/	13.7	/	/
		P_t-test_	1.4E-04	/	/	1.7E-07	/	/
	F_2_	Min	51	/	/	12.4	/	/
		Max	149	/	/	29.2	/	/
		Mean	100	/	/	20.8	/	/
W10	Parents	Zhongshuang11	58	82	140	16.3	17.1	17.1
		No.73290	89	148	237	7.0	8.1	7.8
		P_t-test_	8.7E-03	2.6E-04	7.0E-04	4.7E-07	6.3E-07	6.4E-07
	F_2:3_	Min	48	30	83	5.3	6.4	6.3
		Max	97	183	277	18.7	18.3	18.2
		Mean	76	86	159	13.6	13.8	13.8
W11	Parents	Zhongshuang11	65	126	191	21.0	19.5	20.3
		No.73290	94	173	267	11.4	10.3	10.2
		P_t-test_	2.2E-04	6.4E-03	5.5E-03	1.4E-06	5.5E-07	6.4E-06
	F_2:3_	Min	56	81	136	11.4	9.2	10.1
		Max	104	344	397	22.3	20.0	20.3
		Mean	76	165	233	17.4	15.1	15.9
X11	Parents	Zhongshuang11	45	149	194	22.9	21.2	21.2
		No.73290	76	235	311	12.3	10.8	11.2
		P_t-test_	5.3E-04	2.7E-03	2.1E-04	1.2E-06	8.0E-07	9.3E-07
	F_2:3_	Min	26	80	119	10.9	10.0	9.7
		Max	78	395	456	24.8	23.5	23.8
		Mean	57	197	247	18.6	17.2	17.4
	F_2:4_	Min	37	110	153	9.5	10.6	9.9
		Max	85	284	344	24.5	23.9	25.2
		Mean	60	176	232	17.9	16.4	16.8
Total	Parents	Zhongshuang11	61	119	175	21.1	19.3	19.5
		No.73290	96	185	272	11.1	9.7	9.7
		P_t-test_	1.2E-03	2.9E-03	2.1E-03	9.6E-04	1.2E-04	4.2E-05
	population	Min	52	79	134	10.6	8.2	9.2
		Max	99	236	315	21.8	20.4	20.1
		Mean	75	153	216	17.0	15.4	15.8

PNm/PNb/PNw and SNPPm/SNPPb/SNPPw are the abbreviations of pod number and seed number per pod, respectively, for the main inflorescence/branch inflorescence/whole plant.

**Table 4:** Genetic correlation and broad-sense heritability of pod number and seed number per pod.

	**PNm**	**PNb**	**PNw**	**SNPPm**	**SNPPb**	**SNPPw**
PNm						
PNb	0.49***					
PNw	0.61***	0.96***				
SNPPm	−0.41***	−0.31***	−0.34***			
SNPPb	−0.27***	−0.22**	−0.23**	0.87***		
SNPPw	−0.34***	−0.28***	−0.31***	0.94***	0.97***	
*h*^*2*^	0.65	0.45	0.49	0.83	0.82	0.83

^*^, ^**^ and ^***^represent the significant levels of P = 0.05, 0.01 and 0.001, respectively. PNm/PNb/PNw and SNPPm/SNPPb/SNPPw are the abbreviations of pod number and seed number per pod, respectively, for the main inflorescence/branch inflorescence/whole plant.

**Table 5:** Identified and consensus QTL for pod number and seed number per pod.

**Consensus QTL**	**Peak position**	**Confidence interval**	**Additive effect**	**Dominant effect**	**LOD value**	***R*^2^ (%)**	**Experiments**
*qPN.A01-1*	104.4	94.3–105.8	−4.90	2.52	3.0	12.5	W10F2:3m/X11F2:3m
*qPN.A03-1*	61.5	57.5–61.9	−9.65	7.90	3.6	3.0	W11F2:3w
*qPN.A03-2*	81.6	64.6–98	−1.73	−6.23	3.8	3.4	W11F2:3m
*qPN.A05-1*	10.8	4–13.8	−4.67	9.17	3.4	12.6	W09F2m
*qPN.A06-1*	86.3	82.6–93.0	−6.04	−2.49	8.1	22.8	W10F2:3m/W11F2:3m/X11F2:3m/11F2:4m
*qPN.A09-1*	44.0	42.5–54.4	4.05	−7.20	2.7	2.3	X11F2:3bw
*qPN.C02-1*	8.2	3.2–21.5	−5.14	2.91	4.3	18.5	W10F2:3m/W11F2:3m/X11F2:3m
*qPN.C06-1*	51.2	49.1–53.0	7.29	−7.48	4.4	15.0	W10F2:3m/W11F2:3m
*qSN.A01-1*	17.3	12.4–23.2	−1.01	−1.05	4.2	8.8	X11F2:3b
*qSN.A01-2*	113.9	106.6–117.7	−0.89	1.09	3.7	2.7	X11F2:4bmw
*qSN.A02-1*	6.1	0.4–17.6	0.36	0.76	4.7	5.3	W10F2:3bw
*qSN.A02-2*	43.1	36.2–45.9	1.11	−0.64	5.8	7.0	X11F2:4bmw
*qSN.A02*−*3*	81.6	79.7–82.9	0.32	0.06	4.2	4.3	X11F2:4mw
*qSN.A03-1*	14.1	12–20.1	−1.73	1.33	4.3	6.3	X11F2:3m
*qSN.A03*−*2*	118.0	116.0–123.7	−0.82	1.59	3.1	4.3	X11F2:3bw
*qSN.A05-1*	9.5	4.1–13.7	−1.03	1.47	4.1	4.7	X11F2:4bmw
*qSN.A06-1*	88.9	85.0–97.6	1.97	0.59	11.0	32.1	W09F2m/W10F2:3bmw/W11F2:3bmw/X11F2:3bmw/X11F2:4bmw
*qSN.A07-1*	6.7	3.7–15.9	0.75	0.53	5.9	6.9	W11F2:3bw
*qSN.A07-2*	54.4	35.8–61.1	0.86	0.62	7.5	15.8	W10F2:3bw/W11F2:3b/X11F2:4b
*qSN.A07-3*	75.7	69.6–78.8	0.48	0.52	3.9	3.8	W10F2:3m/W11F2:3mw
*qSN.A09-1*	68.7	68.5–82.2	0.59	0.15	3.6	4.9	W11F2:3bw
*qSN.C02-1*	5.8	2–18.1	−0.71	−0.13	3.8	6.7	W11F2:3w
*qSN.C06-1*	53.4	47.8–58.2	0.72	0.39	3.7	4.7	W11F2:3bw
*qSN.C06-2*	16.6	82.9–97.7	1.03	−1.02	4.6	8.6	W10F2:3bw

**Table 6:** The common QTLs identified in the current and previous studies for pod number and seed number per pod.

**Trait name**	**QTL name**	**Linkage group**	**Marker interval**	**Genomic region (Mb)**	**Reference**
Pod number	*PNLP-A3*	A03	BoGMS0230/BnGMS257	5.1–6.1	Ding *et al.*^9^
	*qPN-LP2-A3b*	A03	MR123/BRMS043	5.1–7.4	Shi *et al.*^8^
	*Sil/dm*^*2*^*_N3*	A03	E32M51_283E/MR12	10.8–13.2	Radoev *et al.*^5^
	*qPN.A03-1*	A03	BrSF0295/CNU098	11.4–13.5	Current study
	*qPN.A03-2*	A03	FITO285/BoSF2828	14.2–15.4	Current study
	*qPN.A3-2*	A03	SA29/CNU276	14.0–15.2	Shi *et al.*^2^
	*PNNP-A5*	A05	BnPHT1/BoGMS1565c	3.3–4.0	Ding *et al.*^9^
	*qPN.A5-2*	A05	E1HM31–130/CNU344	3.6–4.6	Shi *et al.*^2^
	*qPN.A6-1*	A06	IGF1075d/niab37	7.3–11.1	Shi *et al.*^2^
	*PNLP-A6*	A06	H001J23/BnGMS531	8.5–12.5	Ding *et al.*^9^
	*Sil/dm*^*2*^*_N7*	A07	CB10439/MR153b	10.5–13.5	Radoev *et al.*^5^
	*qPN.A7-1*	A07	E1HM34–220/35RXTRAP10-4	11.0–12.9	Shi *et al.*^2^
	*NPP1-LG4*	A07	M9E12750/Ra2A01	/−14.5	Yi *et al.*^4^
	*qPN.A7-2*	A07	niab063/sR7223	14.1–14.4	Shi *et al.*^2^
	*qPN-LP1-A9*	A09	HBr075/H091P21-4	32.5–32.8	Shi *et al.*^8^
	*Sil/dm*^*2*^*_N9*	A09	CB10533a/MR230	32.7–33.0	Radoev *et al.*^5^
	*qPN-LP3-C3a*	C03	BRMS106/pX141aE	3.9–6.7	Shi *et al.*^8^
	*qPN.C3-1*	C03	STS01/pX141bE	/−6.0	Shi *et al.*^2^
	*qPN.C06-1*	C06	BoSF1254/BoSF2507	17.9–18.8	Current study
	*PNNP-C6*	C06	BnGMS118/MR133.1	18.1–18.9	Ding *et al.*^9^
Seed number per pod	*qSN.A01-1*	A01	BoSF2770/niab106	0.5–1.5	Current study
	*qSN.A1-1*	A01	niab71/CNU142	0.7–1.5	Shi *et al.*^2^
	*qSN.A1-7*	A01	E10HM34-230/EST165	/−8.7−/	Shi *et al.*^2^
	*qSNA1*	A01	Na14G06/CB10189	8.2–8.9	Qi *et al.*^11^
	*qSN-OP3-A2*/*qSN-OP1-A2a*/*qSN-LP3-A2*	A02	sR6293a/BnSIZ1-A2	3.0–4.7	Shi *et al.*^8^
	*SNLP-A2*	A02	Na14H11/B049H14	4.2–4.5	Ding *et al.*^9^
	*qSN.A3-1*	A03	CNU210/S13M08-1-157	15.3–15.7	Shi *et al.*^2^
	*SNLP-A3*	A03	CNU270/HBr131	15.4–16.2	Ding *et al.*^9^
	*qSN.A5-1*	A05	CNU257/BRAS063	3.2–3.3	Shi *et al.*^2^
	*qPN-OP1-A5*	A05	BnPHT1-A5/sN12353b	3.2–4.4	Shi *et al.*^8^
	*S/Sil_N5*	A05	MD21/MR113	8.0–12.4	Radoev *et al.*^5^
	*qSN.A5-2*	A05	CNU206/pW161-2b	11.2–12.8	Shi *et al.*^2^
	*qSN.A9-1*	A09	P04M21-130/IGF5222b	1.0–1.8	Shi *et al.*^2^
	*SNNP-A9*	A09	HBr199c/HBr097c	1.2–1.8	Ding *et al.*^9^
	*qSN-LP1-A9a*/*qSN-OP2-A9*	A09	H055O17-4/HBr096	25.1–29.8	Shi *et al.*^8^
	*qSN.A09-1*	A09	BnSF2342-39/BnID082	28.1–29.1	Current study
	*qSN-OP1-C1*	C01	HR-Sp2-170/BRMS175	36.5–38.8	Shi *et al.*^8^
	*S/Sil_N11*	C01	CB10536/CB10357b	35.3–37.7	Radoev *et al.*^5^
	*qSN.C02-1*	C02	BoSF1425/CNU461	0.8–2.2	Current study
	*qSN.C2-1*	C02	pW119/sR12095	1.4–1.7	Shi *et al.*^2^
	*qSN.C6-4*	C06	HUA64-2/Na12E01a	16.9–18.1	Shi *et al.*^2^
	*qSN.C06-1*	C06	BoSF1254/BoSF1126	17.8–19.0	Current study
	*qSN.C6-1*	C06	pW134/AP1a	29.6–32.0	Shi *et al.*^2^
	*qSN.C06-2*	C06	BoSF1951/CNU331a	30.6–34.2	Current study
	*qSN-OP3-C6b*/*qSN-LP1-C6*	C06	BnPHT1-C6/PA28	34.4–36.0	Shi *et al.*^8^
	*qSNC6*	C06	CNU182/CNU052	34.8–35.9	Qi *et al.*^11^
	*qSS.N19*	C09	EA13MC03-150/Na10D07	39.4−/	Zhang *et al.*^12^
	*qSN.C9-3*	C09	IGF5193b/CB10288	39.0–40.0	Shi *et al.*^2^

**Table 7:** List of 33 annotated genes within the fine-mapped genomic region of *qPN.A06-1.*

***B. napus*** **gene_code**	***A. thaliana*gene_model**	**Gene_name**	**Annotation**
GSBRNA2T00001802001	AT2G02370.1	/	SNARE associated Golgi protein family
GSBRNA2T00001803001	AT2G02360.1	PP2-B10	Phloem protein 2-B10
GSBRNA2T00001804001	AT2G02250.1	PP2-B2	Phloem protein 2-B2
GSBRNA2T00001806001	AT2G02230.1	PP2-B1	Phloem protein 2-B1
GSBRNA2T00001808001	AT2G02230.1	PP2-B1	Phloem protein 2-B1
GSBRNA2T00001809001	/	/	/
GSBRNA2T00001811001	AT2G02230.1	PP2-B1	Phloem protein 2-B1
GSBRNA2T00001812001	AT2G02230.1	PP2-B1	Phloem protein 2-B1
GSBRNA2T00001813001	/	/	/
GSBRNA2T00001814001	AT2G02170.1	/	Remorin family protein
GSBRNA2T00001815001	AT3G49520.1	/	F-box and associated interaction domains-containing protein
GSBRNA2T00001816001	AT1G25055.1	/	F-box family protein
GSBRNA2T00001817001	AT2G02150.1	EMB2794	Tetratricopeptide repeat (TPR)-like superfamily protein
GSBRNA2T00001818001	AT2G02150.1	EMB2794	Tetratricopeptide repeat (TPR)-like superfamily protein
GSBRNA2T00001820001	AT2G02080.1	IDD4	Indeterminate(ID)-domain 4
GSBRNA2T00001821001	AT2G02130.1	LCR68, PDF2.3	Predicted to encode a PR (pathogenesis-related) protein
GSBRNA2T00001822001	AT2G02080.1	IDD4	Indeterminate(ID)-domain 4
GSBRNA2T00001824001	AT2G02070.1	IDD5	Indeterminate(ID)-domain 5
GSBRNA2T00001825001	AT2G02061.1	/	Nucleotide-diphospho-sugar transferase family protein
GSBRNA2T00001826001	AT2G02060.1	/	Homeodomain-like superfamily protein
GSBRNA2T00001828001	AT2G02050.1	/	NADH-ubiquinone oxidoreductase B18 subunit
GSBRNA2T00001829001	AT2G02040.1	NTR1, PTR2, PTR2-B	Encodes a di- and tri-peptide transporter
GSBRNA2T00001830001	AT2G01940.1	IDD15, SGR5	A putative transcription factor in gravity-perceptive cells
GSBRNA2T00001832001	AT2G01950.1	BRL2, VH1	Encodes a LRR receptor kinase and associated with provascular/procambial cells
GSBRNA2T00001835001	/	/	/
GSBRNA2T00001836001	AT2G01913.1	/	Unknown protein
GSBRNA2T00001837001	AT2G01910.1	MAP65-6	Microtubule associated protein (MAP65/ASE1) family protein
GSBRNA2T00001838001	AT2G01905.1	CYCJ18	Cyclin-dependent protein kinase regulator activity
GSBRNA2T00001839001	AT2G01880.1	PAP7	Serine/threonine phosphatase
GSBRNA2T00001840001	AT2G01870.1	/	Unknown protein
GSBRNA2T00001841001	AT2G01870.1	/	Unknown protein
GSBRNA2T00001842001	AT2G01860.1	EMB975	Embryo defective 975
GSBRNA2T00001843001	AT2G01850.1	EXGT-A3, XTH27	Homology to xyloglucan endotransglucosylases/hydrolases (XTHs)

**Table 8:** Conditional analysis on the target major QTL region in both linkage and association population.

	**Experiment code**	**LOD/*R*^2^ (%)**			
		**PNm**	**PNm**|**SNPPm**	**SNPPm**	**SNPPm**|**PNm**
Linkage population	W09F_2_	NS	NS	10.0/25.6	11.9/23.3
	W10F_2:3_	9.9/23.9	10.5/19.7	10.4/28.1	11.4/27.8
	W11F_2:3_	6.9/20.5	6.2/18.1	13.4/34.6	8.2/24.2
	X11F_2:3_	7.7/24.0	7.6/19.8	21.8/44.7	18.9/48.2
	X11F_2:4_	8.0/22.8	9.7/20.2	11.3/43.6	12.9/47.6
	**Associated marker**	**−log(P)**/***R***^**2**^ **(%)**			
		**PNm**	**PNm**|**SNPPm**	**SNPPm**	**SNPPm**|**PNm**
Association population	BrSF46-136	4.3/4.6	4.1/4.2	NS	NS
	BrSF46-161	7.4/6.8	6.5/5.8	NS	NS
	BrSF46-167	6.8/8.5	5.4/6.8	NS	NS
	BrSF46-177	7.7/8.7	6.6/7.4	NS	NS
	BrSF46-186	4.4/4.5	4.8/4.3	NS	NS
	BrSF46-238	3.7/4.1	3.0/3.6	NS	NS
