# Supplementary material for: Linkage and regional association analysis reveal two new tightly-linked major-QTLs for pod number and seed number per pod in rapeseed (Brassica napus L.)
Source: Sci Rep. 2015 Oct 5;5:14481. doi: 10.1038/srep14481 (PMC4593047; doi:10.1038/srep14481)
Supplement: Supplementary Information [file srep14481-s1.pdf]

Supplementary Information

Linkage and regional association analysis reveal two new tightly-linked major-QTLs for pod number and seed number per pod in rapeseed

Jiaqin Shi[1‡], Jiepeng Zhan[1‡], Yuhua Yang[1], Jiang Ye[1], Shunmou Huang[1], Ruiyuan Li[2], Xinfa Wang[1], Guihua Liu[1], Hanzhong Wang[1*]

[1]Key Laboratory of Oil Crop Biology of the Ministry of Agriculture, Oil Crops Research Institute of the Chinese Academy of Agricultural Sciences, Wuhan 430062, China.

[2]Key Laboratory of Information and Computing Science, Guizhou Normal University, Guiyang 550001, China.

[‡]These authors contributed equally to this work.

[*]Corresponding aurthor: Hanzhong Wang.

Tel: +86-027-86711916;

Fax: +86-027-86811837;

E-mail: wanghz@oilcrops.cn.

Table S1 Summary statistics for primer-pairs screening for parents and marker genotyping in the BnaZNF$_2$ population.

Marker type	Prefix of primer-pairs	Screened primer-pairs	Polymorphic primer-pairs	Polymorphic markers	Source of primer-pairs
InDel	BnID	76	33	34	our unpublished data
SNP	IGF	98	4	4	http://brassica.nbi.ac.uk/IMSORB/
	ns	294	108	110	Huang et al. 2013
	snap	2200	46	48	Wang et al.2012
SSR	BN	24	2	2	Kresovich et al. (1995); Szewc-McFadden AK et al.(1996)
	BnEMS,BnGMS,BoGMS,BrGMS	602	63	71	Cheng et al (2009); Xu et al (2010); Li et al (2010)
	BoSF, BrSF, BnSF	4275	517	566	Shi et al. 2014
	BRAS,CB	373	35	37	Piquemal et al. (2005); Radoev et al. 2008
	BRMS	40	4	4	Suwabe et al. 2002
	CNU	191	43	49	Kim et al. (2009)
	ENA	13	2	2	Choi et al. (2007)
	FITO	255	6	6	Iniguez-Luy et al. 2009
	HRI	6	1	1	Smith LB and King GJ (2000)
	KS	50	5	5	http://www.brassica.info/resource/markers.php
	MB	5	0	0	Lagercrantz et al. (1993)
	MD	2	1	1	Uzanova and Ecke (1999)
	MR	8	6	6	Uzanova and Ecke (1999)
	Na,Ni,Ol,Ra	398	24	27	Lowe et al. 2004
	nga	30	0	0	Bell and Ecker (1994)
	niab	144	20	23	Kim et al. (2009)
	SA	3	1	1	Qiut et al. (2006)
	sN,sO,sR,sS	85	28	29	AAFC Consortium
STS	AT, IFLP,TF	179	12	12	Sun et al. 2013
**Total**		**9351**	**961**	**1038**	

Table S2 List of 561 newly developed and mapped markers (499 SSR, 31 SNP and 31 InDel) on the BnaZNF2 linkage map.

Marker_name	Marker_Type	Forward_Primer (5'-3')	Reverse_Primer (5'-3')
BnID035a	InDel	GGAATAAGTTGCTTGGGTTGC	GCCAGCGAGGTTCTCAGC
BnID040	InDel	TTTCATCCAAGGAGTAGCGG	CCGTTGACAAAAAGGGCTAC
BnID041	InDel	GTTCCGTTCGGGTTCATCTA	GCTCTTCGAACACGTTCTCC
BnID042	InDel	TTTCCAACTGGAACGCAAAT	GTGACCAGGCTCATCGAAAT
BnID043	InDel	TTCGATACCACCACCAGACA	CATCCTCCTCCTCCATTTGA
BnID044	InDel	CCCATGAGGACAAGGAAGAA	CAACCGTTTGTCAAAGACTGA
BnID045	InDel	CCTGGTTGTGCATTTCCTTT	ATGGCTTTACACGGAGCAGT
BnID046	InDel	CCAGCCATGAGAGTTAGGGA	TGGTGGAGTGTTGGTTTTTG
BnID047	InDel	CCCTTTCAATATGAGAGAGAGGA	TTAATTTGGATATGCATGGGC
BnID052	InDel	GAGGGAACAAAAGAGAGCCA	GGATCACCAGCTGAGGAAGA
BnID053	InDel	GCTCATACCGATGAACATTCTTT	TGTTCGGATAAGCCTTCTTGA
BnID056	InDel	TGGTGCACTACAAGAACATGG	ATGACCCCATGCAACCTTAC
BnID060	InDel	CAAGCATCGAGTTCCACTCA	CATTATTGTTACGGCCCGAT
BnID061	InDel	ACCCCATTGTCTTGGAATCA	CGGAATAACTGCACCGAAAC
BnID062	InDel	GCGACACGTCTCTGGAGAAT	TGCAAGTGCATCCAAAGTTC
BnID063	InDel	ACGCTTTTCCCCTTAGGAAC	GCACATTCTTCCCTTTTTGC
BnID075	InDel	CGCCCTTCAAATACAAGCTC	CTTCCTGAAAGCGTCTACCG
BnID076	InDel	AGCTAAGAGCTGAAGCACGG	AGATGCTGAAATTCGTCTGTGA
BnID078	InDel	TTATGAGGAGGTGACTGGGTG	GCGCAGACACCTATGGAAAT
BnID079	InDel	CAGTCCTGAGCTATTTGGTCC	AACTACGGAAGGGTCAAGCC
BnID081	InDel	GGAGGGAGGGTGCAAGTATT	CACCAACCGTTTTTCATTCC
BnID082	InDel	TACGACCCGCCAAATATCAT	GACGTTTTGGGTATCTGTTCG
BnID085	InDel	CCCTTCCTTCTCCATTCTCC	GGCCCATTAAACTCTCACGA
BnID086	InDel	TCTGTTCTCATGCTGTGGCT	TCCCCTTGGTGTTTTGTCTC
BnID087	InDel	CTTCCCCGACCAAAATGAT	AAGCAAGAGGGGAAAAGAGC
BnID090	InDel	CTCGACGTCTGGGAGATCA	ATCGGCATTCCGTGATAGAT
BnID094	InDel	CACATGAAACCCACCACTGA	GGCCCGTACTATGATCCTCA

BnID095	InDel	GACAACGGCTAGGGTTTCAA	TGAGAGCTCTGTTGCGAAAA
BnID097	InDel	TTGCTTTAGCGGAGCTTGTT	GCAAGCCGGTTATTGAATTT
BnID107	InDel	AAGCACACGTGTCCCACTTT	TGCAGCCAAAAGAGAACCTT
BnID108	InDel	AACTCTCGGCCCATGAACTA	CGGTATCGGAGGAATGAGAA
BnSF2342-39	SSR	GGCGAACAAGAAACCGATTA	TTCCGAATTAAACCGCAAAC
BnSF287-186	SSR	CAGATACATGTGAAAGTTTGAAACAA	ATGTAAACCTCCCCGGTTTT
BnSF400-253	SSR	ATCACCAATGCCATCCTCAT	ATTCAAGACATTTGGCCTGC
BnSF400-28	SSR	AAATTGCAACCCCATAACACA	TCCCACACTAGAGAGAGCGAA
BnSF400-29	SSR	TTGAATCGAATCAGGAAGGG	CATTTAGCAACCTCCACCGT
BnSF400-54	SSR	TGTCGTTTGATGGATGCCTA	GAGGACTCGGTCTGAACTCG
BoSF0016	SSR	GGCGAGGAGGTTGTTGATTA	CTTCGACGGAGATTTTCGAG
BoSF0017	SSR	GGGCATTTCTTTTCGTGGTA	CTCACGGACTTTGCTGACAA
BoSF0022a	SSR	TGCCATCAAAGGAGTGTTCA	TCATCTGCTCAACATGAGGC
BoSF0022b	SSR	TGCCATCAAAGGAGTGTTCA	TCATCTGCTCAACATGAGGC
BoSF0023	SSR	CCCTATTTGCGTGCGTCTAT	GAAACCACCCATCAAGGAAA
BoSF0033a	SSR	GTTGTGGTCTGCCAAAAGGT	CGCAGGAAACTGTAAGAGCC
BoSF0040	SSR	CCAAAATTTGCATGAAAGCC	CAGCTTGTGCTTGTCTTCTTG
BoSF0050a	SSR	TGTACGTGCACCACGTTTTT	CTTCGATTACTCGGTGGCAT
BoSF0050b	SSR	TGTACGTGCACCACGTTTTT	CTTCGATTACTCGGTGGCAT
BoSF0096	SSR	CTTGGTCCGTTGCTCTTTTC	AACTGATCGAAAGCTCGCAT
BoSF0114	SSR	TCTGCCAAATTGACCAGAGA	TGTAAATGGAACAAGCAAGCA
BoSF0130	SSR	AAATCCAAACCCACAACCAA	CCCATCACAGAGGCAAGAGT
BoSF0132	SSR	GGTCATCCACATTGGTTAGAAA	GAGGAGTGTCCAGGACCGTA
BoSF0133	SSR	CGTTTCGAGTGTCTCACCAA	TCGAGGCTAGGTTTTGCAGT
BoSF0160	SSR	GAAGGTCCTTACATGGTGAAGC	AAACACAGTGCTGATGCAGG
BoSF0176	SSR	ATGAAAACCACAATCACGCA	TTTCGATGGCACAAAAATTG
BoSF0185	SSR	ATGGACTGTGTTTTTGGTCG	ATGAAACCAAGGCAAAATGC
BoSF0197	SSR	CGGATTCATCAACCTCGTCT	CTCACCGCTTCAAAATCCAT
BoSF0213	SSR	GAAAATCCTGCAAACCCTGA	CTCTCCATATCTCCGGACCA
BoSF0215	SSR	CAGGTCACTACAAGTGGGCA	TCCCTATCCATGAGACGAGC

BoSF0216	SSR	GAGCCTTTGCACACGAAAAC	GGCGTCTAATAGCATGTACAAGTG
BoSF0222	SSR	CTCTTCCCACCGGTATTCCT	CGAGTTCACCGAGCACAATA
BoSF0223	SSR	AGCTCACGAATCTGCTCCAT	CCGAAACCAAGATAGGTGGA
BoSF0224a	SSR	TTCACAAGGCTTCTCAGCAA	CTGATCTTGGAATGCCGTTT
BoSF0224b	SSR	TTCACAAGGCTTCTCAGCAA	CTGATCTTGGAATGCCGTTT
BoSF0230	SSR	GGCAAAAGAAGAGACAGCGA	AGTGGGGGACCATAAACCTC
BoSF0231	SSR	CTCGTGGTTCAGGACTCCAT	GTGGCGAGTTTTGATTTGGT
BoSF0232	SSR	CAGACTCTTTGACCGCCATT	GCTCCGGTCAGATCCATAAA
BoSF0239	SSR	TGTGTGGAACAGTGTTTGGAA	TCTCTGGCTCTCTTTCGCTC
BoSF0259	SSR	GTGGTTGGAAATATCGACGC	TGAAGCTCACAAATCGCAAG
BoSF0270	SSR	TGAACCGTGACAGAGAGTGC	CCTCACCCAAAAGCCAAGTA
BoSF0284	SSR	TCTCTACTTTCTCGGGTGGG	GCGTGTTCTCCTCCCTCTAC
BoSF0302	SSR	CATGCGGAAGCATTTGAGTA	CCGATCGAGCTCATGATAAA
BoSF0310	SSR	TCCCTCAACATACCCATTGA	TGTCCATGACCATTGCTTGT
BoSF0318	SSR	CAAACAGGCCCGTCTCTAAG	AGGTGGAGCTGCATTTGATT
BoSF0329	SSR	AACCGTTACAGACACCGCTC	AGGAAGCTTTATCCCCCAGA
BoSF0333	SSR	CGATCAGTACCAGCCGAAAT	TCTCTCAGCTCCTCAACCGT
BoSF0336a	SSR	CCGAATCTTCCATTTACCGA	CCATGTCCATCTTCGCTTTT
BoSF0336b	SSR	CCGAATCTTCCATTTACCGA	CCATGTCCATCTTCGCTTTT
BoSF0346	SSR	AAAACATTGGGGCAAATCAA	GCGAGACGTATTGTTTGGGT
BoSF0349	SSR	AAACCCACACATCATCTTCCA	AGCATCTTTGCTCTTGGCAT
BoSF0365	SSR	GAGATTGGAAGAGCGCAAAC	ACCCCAAGCCTGGTTAGACT
BoSF0371	SSR	TCAACAAACCAGTAACCACGTT	CAACACGAGCCAGAGATGAA
BoSF0393	SSR	TCGAATGGTTGTTTGTTGTTG	GCGAGACGTATTGTTTGGGT
BoSF0402	SSR	CCTTACGCAAGACCAGAAGC	GACTCGAAGCTGTGGTGACA
BoSF0415a	SSR	AGCAGAAACGAAACCTGCAT	TCGTTAAGGTGTGGAGTGATTG
BoSF0415b	SSR	AGCAGAAACGAAACCTGCAT	TCGTTAAGGTGTGGAGTGATTG
BoSF0421	SSR	GGACAAAAGTGTGTCATGCG	AAGGTAAGCCGTTCGGTTCT
BoSF0442	SSR	TCGTTGGTCTTGCCTTTCTT	CGTATTTCTTGAGTGCAAACG
BoSF0448	SSR	CTTCTCCAAATCCTGGTGGA	AAGCATCCAAACCCATCAAC

BoSF0455b	SSR	TCGAATGCCATATCACAGGA	ACCACCATAGCCAAAACCAA
BoSF0455c	SSR	TCGAATGCCATATCACAGGA	ACCACCATAGCCAAAACCAA
BoSF0466	SSR	TAGCATGTTCGAACCGTCAG	AAATAACTGGACGCCCAAAA
BoSF0482	SSR	GGTGATCAACCATTGCAACA	GACTCCACCGAAGGTACCAA
BoSF0483	SSR	GCATCTGATCTTTCCGCTTC	GGTTTTGTTTTGATTGGGACA
BoSF0495	SSR	TCTCCTTGCATCGGCTAAGT	TTTTGGGCTGTTTAGTTCCG
BoSF0503	SSR	ACCATGGCCTCTTGTATTGC	CGGAGTGTTCCCAATCAACT
BoSF0513	SSR	CCTTTTACAAAGGCAATTGAGA	AAAACCCTGAACACACCGAG
BoSF0521	SSR	CTCACGAGCTATTGATCGCA	CACGAATGATGAGCCAAGAA
BoSF0525b	SSR	GGAGAATAGCAGGCAGCAAC	TTGACCCAAGCATGCAAATA
BoSF0546	SSR	GCGTAGGTCGTTGACAGACA	GCTCCCATGGTAGTGTTGGT
BoSF0552	SSR	GCCCTAAACATGGCATTGTC	GGGTTTCTTCAAACACCAGAA
BoSF0554	SSR	TGCGTCACAACCCTTTCATA	TGCGCGGATCTTAAGCTAGT
BoSF0560	SSR	GGATTGCAGAGGACGAGGTA	GCGCAATTGCTGTTAAAAAGA
BoSF0582	SSR	CACACATGGTGGCTTTCTTG	GATTTCCCGACAAAGTTCCA
BoSF0586a	SSR	GAACAGACGAAACGACAGGC	AGCACCAAAAGCTATCCCAG
BoSF0592	SSR	TTTGAAACGGAGGACATCAA	CCATGTGATCGCCATTGTTA
BoSF0598	SSR	GTATGAAGGACAACGCGACA	TTAGCAGACTTTAGCGCCGT
BoSF0600	SSR	GGGGCCCAACTTTTACCTTA	TATTTTGTTTGCAGCCGTTG
BoSF0601	SSR	AAAACCCCACTTTTGACGTG	CCGTCGTTATCATCCTCGAT
BoSF0613	SSR	TGGAATTGTCTCCCCAAATC	CGAACTGAACCCGAATGAGT
BoSF0643	SSR	TAGCCCTACTGGCCCCTATT	CGCACTCCGGTTTCTCTAAA
BoSF0652	SSR	TCTTCCTCCTCCCTCCATTT	ACGTAAACCTGAGGAGCGAA
BoSF0664	SSR	TTCTCTGCGTTTTCAGGTGAT	CCTAAGAGCCGTGACATGGT
BoSF0665	SSR	AAAATCGGGGGTTATCTTGC	TTGGCCAAAGTTTCTAGCGT
BoSF0670	SSR	ATCTTCCTCCACGTTCATGC	TGAGGAGTGTGTGTGTGTGTG
BoSF0678a	SSR	AACTTTCTCTCCTCCACGCA	TTTCCGCCGATAAAAAGAGA
BoSF0693	SSR	ATGCATCTTAGCTGGCACAT	TTTCTCGCCAGAATCGAACT
BoSF0707	SSR	ACTTCCAATGGGGGTTCTTC	GGCGTTCTTTGCTATACCGT
BoSF0711a	SSR	GAGGCGATTCGTCGTTCTAA	ATTGGCCATTTCAGTGAGGT

BoSF0711b	SSR	GAGGCGATTCGTCGTTCTAA	ATTGGCCATTTCAGTGAGGT
BoSF0718	SSR	CAAAACGTGGCTAACAACCA	TTTCGTCGGTTTAGGTTTCG
BoSF0733	SSR	CGTGGACATAACGGACAATG	CTTGAAGCTCTCCAGCAAGAA
BoSF0757	SSR	CAAAAGTGGTTGTGCATTGG	GCTCTTGGAATGGCGTCTAC
BoSF0760	SSR	CCAAACGGTCATTTCGTCTT	CAGGAGGAAGGAATCGATGA
BoSF0764	SSR	CCCAAGTTCGCTTAATGGTT	GTTCTGGGGCAAATGACTGT
BoSF0778	SSR	GAGTCCCACCGATCAAGAAA	GATGAGAGAGATCAGGGAAGGA
BoSF0783a	SSR	GCCTTGGATGAAGACAGAGG	GCGGTGAAGGGTGTAACTGT
BoSF0783b	SSR	GCCTTGGATGAAGACAGAGG	GCGGTGAAGGGTGTAACTGT
BoSF0784	SSR	GCTTTCTCAGAGGTGCGTTC	CACTTCCGTAGGAGGTGCAT
BoSF0805	SSR	GCCCCATCCATATACCATTG	ATATGGAGGCGTGGCATTAC
BoSF0840	SSR	GCGAAGCTTCCTTTGTCATC	ATCTGAGACGGAGCGTGAGT
BoSF0841b	SSR	AGCGGTGGAAAGCTACTTCA	CAACACCGTCGATCCCTATC
BoSF0869	SSR	AGCACCGCCGATGACTATAC	TGAAGCGAGAGGACGAATCT
BoSF0872b	SSR	TGGAAATAGGCATATTACATTGG	GAAGAGCAGGAAGTTTTGGC
BoSF0880	SSR	ACGATTCCAATCACAAAGGC	GTCGTCGTTGCTCTCCTTCT
BoSF0883	SSR	TGAATGTGTGTGTGTGGAGTG	GGCGCATCCAATACCTAACA
BoSF0898	SSR	ATTCAGTACAAAGGCCGTGC	TGGAATGTGCTTCAGAGTCG
BoSF0911	SSR	ATTCCCGGAGGATCAAAAAG	GATGACCAGTCAAAGAGCCC
BoSF0926a	SSR	TTCCAACAAACTCATGCCAG	AACAAAAGGGATGACGTTGC
BoSF0926b	SSR	TTCCAACAAACTCATGCCAG	AACAAAAGGGATGACGTTGC
BoSF0926c	SSR	TTCCAACAAACTCATGCCAG	AACAAAAGGGATGACGTTGC
BoSF0935	SSR	TGGCGATCGAGTTATGTGAA	GTCCGGTTATGTCTGTGCCT
BoSF0943	SSR	AGCCGCGTTAATTTGCTAAG	GGGGAGTGGGTTAGGGTTTA
BoSF0948	SSR	GGCCAAGAGAAAAGGAAACC	CGTCCATAACCGTCTCATCC
BoSF0949	SSR	TCAGATTAAGGGAGCAACCG	AAGCAGCCGAAAATGCTAAA
BoSF0952a	SSR	GGGTTCATATCAAAAATAAAAGCA	TGTTGCATAGAGTTCTCCATAGAT
BoSF0952b	SSR	GGGTTCATATCAAAAATAAAAGCA	TGTTGCATAGAGTTCTCCATAGAT
BoSF0955	SSR	ATCACCAGCATCACCATGAA	TGCTCACATAATTGTCAATACAGC
BoSF0960	SSR	CATTGTACATGACCGCCAAC	TGCGTGCTCTTTTGATATGC

BoSF0980	SSR	CTAGCACCATTGGATCGACA	CCCCGAATCAATCATCAAAC
BoSF0997	SSR	TTCACATGATAACACCATTGAACA	CCAAACTCTTAAAAAGGGACCA
BoSF1005	SSR	CAACCTCGGATTTTGATTCG	ATTCATTTGTGCAGCAGTGG
BoSF1009	SSR	TCGCTGAAGAGGAGGAACAT	TTTCTCTCCTCGCTGGGTTA
BoSF1017	SSR	GCCACAAGTGTTTCCTTTCC	CACAACTTTGCCGGTGTATG
BoSF1064	SSR	ATGCCCGCAAGTATAAAGTAGT	AGTACCGTCACCTGGATTCG
BoSF1085	SSR	GCCACACATCAGACACCAAC	TCTGTGGCCATGTTAGTGGA
BoSF1088	SSR	TTCAAACTTTTGGCGATTCC	ACGAAAACAAAACAGGTCCG
BoSF1098	SSR	TTCAGCGTTGACTCTTCCTG	ACTCCTTTGTTGTTCCGTGC
BoSF1121	SSR	TGTGTACCTCGCCTAGCCTT	GGTGATGTTTGTTTTTGGGG
BoSF1125	SSR	GCTGACCAGATGGAGAGCAT	TCCATGTACTGCCTTTCACAA
BoSF1126	SSR	TCATGGGGAGGACAAAAGTC	GCTGCTCTTTTTGTTGTGTGG
BoSF1136	SSR	ATGCAATCACACGGAATCAA	CTTCGTCTTCTTCGTCACCA
BoSF1144	SSR	CCGCACCATCGAAAATAAAT	TGGTGGTTTTTGCATTTTTG
BoSF1161	SSR	GACTTCATACAGCTGCCCGT	CGAATTTTCTCCCAAACATCA
BoSF1212a	SSR	TAAGGAAGCAATCCACCACC	CTTTCCCAATCCTTTCCCTC
BoSF1212b	SSR	TAAGGAAGCAATCCACCACC	CTTTCCCAATCCTTTCCCTC
BoSF1222	SSR	CACTTGGACTACCGCTGTCA	TAGAAACAACTTGGGACGGC
BoSF1228	SSR	TCTCTTTGAAAATGTGGCCC	TATGTGGACGATCTTTTGCG
BoSF1230	SSR	GGAGAAAGTGGTGAGCTTCG	ATTCTTTACACGACCGCCCT
BoSF1254	SSR	TGTGGGTGTAAGATATGCGTCT	ATCTTCCCATAAGGCACACG
BoSF1258	SSR	AGGAGGAGGAAGAGCAGAGG	GTGTGGTCGTCAATTTGTGC
BoSF1264	SSR	TTGTTATCTATATTTCCAAACAAACCT	TTGTTTGCAATGGTTGTTGTT
BoSF1270	SSR	GCATTGATTGGATGATTCAAAA	ACTGATCTCCTCACCCGTTG
BoSF1271a	SSR	GGATGGCAGCTTTTTGTGTT	AATTCTGATACGCAGGCACC
BoSF1271b	SSR	GGATGGCAGCTTTTTGTGTT	AATTCTGATACGCAGGCACC
BoSF1276	SSR	CCTTGCATCTCCTCTTCCTG	ATGCAGCCACCTTCATATCC
BoSF1319	SSR	AGGAACGGTTGCTTACGAGA	ATCCCTCAGCTCACTCCTCA
BoSF1324	SSR	CCAACACAGGTTACACCAACA	TTGGAAGCCATGAAGACGTT
BoSF1331a	SSR	ACTGGGCTGGCTGCTAAATA	AGAAATGCGCGTTTTTAAGG

BoSF1336	SSR	TTTCATTCGGGAAGATCAAAA	TCCGTAGCTATTTCCTTGATTTCT
BoSF1365	SSR	CGCCTATAACATTAGTTGTCGC	GGTGACCAATGAAATTGGAA
BoSF1372	SSR	CGCTGCCGGTAAAGTTTCTA	CTGCAGGATTGCATTTAGCA
BoSF1406	SSR	TACCATGAGTGCAAAACCCA	GGAAGCTGCTGGTTCAGTTC
BoSF1414	SSR	CCCCTATGCACATGGAAACT	CGGTAAAGAATCTTCAAGCCC
BoSF1425	SSR	TTTTATGTTTCGGCACATGG	CCCTGCGAGCCTACAATAAG
BoSF1468	SSR	TTGTTCTCCTTTGTTTCTGATCC	CTTTGTTTCTGGGTTGAAGTTG
BoSF1469	SSR	AAAAACAAAAAGCAAGGGGC	GAGCCAAAACAGCAATCACA
BoSF1476	SSR	GCATATCACAGACCAGTCGC	TTTCCGTACTCGGTAGGAGG
BoSF1481	SSR	TTGCTATTGGCATAGCTTCTTG	GGGAGTTGTTGAAGAAGGAGG
BoSF1492	SSR	CGTTGTGCTTTGTGAGCATT	CCTCCATTTCCAATCGAATC
BoSF1509	SSR	GTGCGGTGGTCAAGTTTTCT	TGCGGCATTAGTTGTCAGTC
BoSF1510	SSR	TGACATTGACGCCATTAGGT	GCCAGCTCAGTTTAATTCGG
BoSF1521	SSR	CACTCCAGATGTGTCGTTGG	ATGAGATGGGAGGATGGTGA
BoSF1522	SSR	AGTTGCCTCCTCTGCAAGAC	TCCCCAACTATCACGTCCTC
BoSF1539	SSR	TCATACCCGGATGCAATTCT	TGAAGGATCCGTCTTTCCAC
BoSF1555	SSR	TGCCATCAACTGAGACGAAG	GAGACGAGACTTCAACATTGTTTTT
BoSF1559	SSR	GCCTGTTTATCTCACAAATACTTTCA	CTTGGGATTTTGACCAAAGG
BoSF1597	SSR	TTGCCAGCTTCAGACCAAG	CGATTGGAAGATCTCAGAGGA
BoSF1642	SSR	ACCAGCAAGTTGGGATGAAC	GGCTGTCTTCTCATTGATTCAG
BoSF1647	SSR	TAACCAACAACCTCCAATGA	CAATCCGAGAACAAAGATAAA
BoSF1650	SSR	CTATTCAGCTCCAAGCCCAG	TCATCATTCCCTCCAACCTC
BoSF1651	SSR	TGGGATCCATTTTGTTAGCC	TCATTGGTTTATGATTTGTAGAGCA
BoSF1659	SSR	CGGTTTGTTCCTTGACGAAT	CCATCAGCAACCTCTACTTGC
BoSF1663	SSR	TCTGAGGAATCGAAATTGGG	GCGTACACTTGTCCGGTTGT
BoSF1665	SSR	TTGCTTCCTCCATCCCTAAA	CAAAATCTTTTTAAATTCTTTGGATCT
BoSF1666	SSR	CTGGCTCTGTCTCTTGAGGG	CCAACCACAAAGGAGGAAGA
BoSF1667	SSR	TCACCCTATGCACCGTTAGA	AACTCAAGGCCGACAAAGAA
BoSF1678	SSR	CTTTCTGTCTCTCCCTCATTC	CGCTGTTATCCGACTCCA
BoSF1692	SSR	CAAGCTGCATCCATCCATTA	ATGTCATGGAGCCCTCGTAG

BoSF1704	SSR	CGATCACTTGGATTCGGTCT	CGCGATTTTACGGAGAAGAG
BoSF1709	SSR	CGACACGGAGCTTTAAGGAG	CGCGTCATCTACGCAAACTA
BoSF1714	SSR	AAATTGCCGAAACCCTAAGC	CGACGAAAACCCCTCTGTAA
BoSF1715	SSR	GATACACTCCGGGTCTGGAA	GCAAAAGAGAGGCACGTTTC
BoSF1722	SSR	CACAGGGATCGAACTCCATAA	CGGTTCCTTAAAATATGGGTGA
BoSF1743	SSR	GCAGTGATGACCACTCTTTTCA	GTTCCATATCGCTGGCGTAG
BoSF1749	SSR	AGCTTGAGAACCCTCCAACA	AGACTCGCTCGGTGATGATT
BoSF1752	SSR	GAACTTGCAGCAGGACATGA	ACTCAAACAGAACCGAACCG
BoSF1779	SSR	GGAGGGAGAGAGAGAGAGGG	TAAACCGGTATTGGGTCGGT
BoSF1788	SSR	ACGGATGTGAGGAGATCAGG	TGATGCTGTCCAGGTACCAA
BoSF1799	SSR	GTTCTCATTCGTCTTGGGGA	CGTGAGGTCATCTCCATCCT
BoSF1808	SSR	CCTCTGTCCGCATATTTGGT	CTCTTGGACATATCTCCGGC
BoSF1810	SSR	ACCCCTGTTAAAAGAACGCA	GTTCGCCGAAAAACACAAAT
BoSF1814	SSR	TTGTGACTGTTACCTCGGCA	TATGCATATGGGTTGCTTCG
BoSF1817	SSR	TGAAACAAACAAAGCCTGTGA	TTGTTTTCCAACTCTTGGGG
BoSF1825	SSR	CGGTAATTGTTTTTGTGGCA	CCCTGCCATTAGTGGAAACA
BoSF1826	SSR	TGAGATGGTCCCTGAGAACC	CCCTTTTCCCCTTCTATTGG
BoSF1830	SSR	CCGACATCGGAAGATTAGGA	CCAACCTATTACTGGCGGAA
BoSF1843	SSR	TCTCCACGTTGTCCATCAAA	TTTAATCAACGATGCTTGCG
BoSF1852	SSR	TTGGGAGAGCGAGAGTGAAT	TGGCTGATTATTGACGAATTG
BoSF1872	SSR	CACTCAGAGGAGGAGGTTGC	GCCACGTGTAGGCATGTAGA
BoSF1885	SSR	CCCAATGGGGTCTGTTTTAG	GAACCATGTTGGGTCCTCTC
BoSF1886	SSR	TAACCCTCGGGTCACAATTT	ATCTTGTCTTCATCGCACCC
BoSF1902	SSR	GCCAAAAACATGAGAGCACA	GCGAAGAGCACAGATGATGA
BoSF1911	SSR	ATCCTCGGACAATCAAATCG	CCCAACCAATTATAAAAACATGC
BoSF1918	SSR	TACCCCTCTGCATCTTCCAG	GGGGCTTGGTATACATTGGA
BoSF1919	SSR	AAACGCCCAAGGCTTCTTAC	CGGTGTTGTGAAATGGTGTC
BoSF1920a	SSR	CAGGCTCGAGACAATCCAAT	ATGTGAGGGGTGGTTGTGTT
BoSF1920b	SSR	CAGGCTCGAGACAATCCAAT	ATGTGAGGGGTGGTTGTGTT
BoSF1924	SSR	ACCGTCAATCAGAAACGGTC	CTAATTAACCACGGAGCCGA

BoSF1926	SSR	TGGCTTGCATTCAGCTAAGA	CCTTCCTGTTTCAATCCTCG
BoSF1937	SSR	TCAAACGTTTTGCTGTTTGC	AGATTGAAGCTATGCGGAGG
BoSF1939	SSR	AATCGAGTTCTTTCTCGCCA	CGAGAGAGCAAACCAAGTCG
BoSF1944	SSR	TGTGATTCAAGAACAGCCCA	CACACGCACACCTACGACTT
BoSF1951	SSR	GAAAGTGCCTTCCTTGCACT	CCCTTTCACTATTTCCAGCA
BoSF1969	SSR	TGCAGATTGATGGGTTGAAG	CCCTAAGGTCGTTTTCATGC
BoSF1970	SSR	TGACCCAGCTATCGCACATA	GGTTCTTCTTGCCGTCGTAG
BoSF1972	SSR	TTATCGACGCCGTCTTAACC	TTGCTTTCAATAGGGGATGG
BoSF1976	SSR	TCGGGGAAAGCCTCTAACTT	CCCTGGTCCCAACCTTTAAT
BoSF1977	SSR	ACATGTACATAGGCCCCCAA	ACGCCAACAAAGGAATCAAC
BoSF1990	SSR	AGGGGAAGGGAAATGAGAAA	GCCTAGCGCTAAAGTGATCG
BoSF1995a	SSR	ACCCCAAACTATCCCCAAAG	ACTCAAAACATGGCGGTTTC
BoSF1998	SSR	CCCCAGTACCAGTGATAGCG	TTTTAGGATTGGAGGAGGCA
BoSF2005	SSR	GGAAACGTTGAGATGGGAAA	CTGATCATCTCAGGCCACAA
BoSF2006	SSR	GGATTGGGGTGAGAAAGGTT	ATATGCCGCAACACCTGAAT
BoSF2018	SSR	TTGCTCATTCAGCACGAATC	GAAAACGTCCCTCCCTCTTT
BoSF2019	SSR	GCAACTCAACAGTTGGGAAAA	TAGGCGCCAATGTTAATGCT
BoSF2030	SSR	GCTGCTTCAAGGAGGTTCAG	CCATTGGAACCTGGGAATTA
BoSF2040	SSR	CCATCTTTTACGCCCTCTCA	CCCGAAAGAGACAGTTTCCA
BoSF2062	SSR	CTCCTACCCCTCTAAACCGC	GCCAATTACCCACACTTGCT
BoSF2071	SSR	GAAATGGAGTAAATATGTTACGGCTAA	TGCAGAGTGAAGTGGTGAGG
BoSF2073	SSR	TGACCAATTTTTATTTCCCCC	CTTGGTCTAAGGCATCCGAA
BoSF2100	SSR	TCCCCTTTTCTTTCTTGGCT	ATGCTGAAACTCTCCCCCTT
BoSF2104a	SSR	GACTTCCATCGAGCACCTTT	ACTGGAAACTCTTCAGCGGT
BoSF2106	SSR	GAGTCATCGCAAGGCTCTTC	TCACCCACCCACCTATCCTA
BoSF2123	SSR	TTCAAGATTCGGAGACCAGAA	CTCCGAGCTTCCATCATCTC
BoSF2131	SSR	CCCAAAGCCTCCTTCTCTCT	GAAAACGAAGAGGTGTCTATAAGAA
BoSF2132	SSR	ATGCCTGGGAGATTAACGTG	GGACAGCCTATCTGAGGCTTA
BoSF2141	SSR	GGATCCCGTTTCTGAACTGA	TACATCGGCGGTTCACAGTA
BoSF2145	SSR	TGGTAAATGTCTCGGCTTCC	CGCAGGCTTCTGTATGTGAA

BoSF2163	SSR	GGGTGGAACTTCAACTCCAG	ACATTTCCACGTCAACCCAT
BoSF2181	SSR	CGACAGCTGCATGAGGATT	TCAGGAGATGGCTGTGAAGA
BoSF2182	SSR	CTCATGATGACTTGGCGTTG	TGGAAAGGGAAGCATCAAAC
BoSF2214	SSR	TCGATTTACGTCTCTCACGG	AGGAATGTGGTTGTGGCATT
BoSF2220	SSR	ATCTGAACATGGGGGATTGA	TGACAACAAATCTTTTCAAGAGTCA
BoSF2224	SSR	CCTACAGCAGTAACGGCTCC	AATGACAGGTCCCCTCCTCT
BoSF2233	SSR	CGCAACCATGTGAAGAAGAA	TCTACACTGAACCCGAAGGAA
BoSF2242	SSR	CGTTGTCTCAAATCAATGGC	TCATCCATTATCATCGGGGT
BoSF2253	SSR	CGAAAGCAATAGCCTATATCCG	GCATGCATGGTTACATTTTCA
BoSF2257	SSR	CGTGCGGAGTATTTCCATTT	CAGAGCTCGCTTCTGGACTT
BoSF2258	SSR	TCGATTTGAACGGAATGGAT	GGCCTAATGGGCTTCACATA
BoSF2263	SSR	TCCTATCCAACATGTTCCTCC	TCATCGATCTGCTTCTTTGTATG
BoSF2269	SSR	GAGCATCATTAACGCGGTCT	CTTCATTTGCTGGGGATCAT
BoSF2273	SSR	CACGTAGACAAGAAGCGCAG	TTTGTTCATTTACTCCCGGC
BoSF2274	SSR	CTTCTTAGGTCGTTCCTGCG	AAACCCCACCATAGATTCCC
BoSF2277	SSR	GAGAATCCAATCCGGACAGA	CACGTTCAGGTTCCCTGATT
BoSF2281	SSR	CGATCTTCTCCTCTCTCTCCAA	TGTGACGATCAACCATTGCT
BoSF2284	SSR	CGAGATCAATAGGTAGCGGC	ACGTGGTCTCAGATTTTGGG
BoSF2290	SSR	GCCTCCGATCAACAAGAAAA	ACCAGCATGGTCAAGTCGTT
BoSF2294	SSR	ATCTCGAATCGCATTGATCC	GCCCTCGTGTCAAATCCTTA
BoSF2314	SSR	CAGCTTCTGGTGATGCGTAA	CCAAGAAAACAAACGGCTCT
BoSF2316	SSR	TTTTGAGTTCACAAGGGTTGC	ACCTGAGCCTGAAGTCCAAA
BoSF2324	SSR	AGCTTCACTGCGAAGGAAAA	TCTTTCCCATTTTCACAGCC
BoSF2344	SSR	CCTGAAACGAATCGGTGAAT	AGCTCCGACCTTTCTCAACA
BoSF2346	SSR	TTAAGCCAAGCTTCCCAAGA	GGCTGTTGCGTATTACCGTT
BoSF2347	SSR	TGTTTGACGAGTGAGTTGGC	TACAGGAGTGGGGATTGCTC
BoSF2360	SSR	GCAGGTGAGGATCGGAATAA	ATTCCCGAGGAGAACTGTCA
BoSF2378	SSR	GAGGAAATGAGATCCATAGGTCA	GGAATGGAAATATTCTGAAACTCC
BoSF2392a	SSR	TGACCAATCCCTTATTGTGGA	TGTTTTGATTAGCTTGTCGATTG
BoSF2392b	SSR	TGACCAATCCCTTATTGTGGA	TGTTTTGATTAGCTTGTCGATTG

BoSF2393	SSR	CTGGCCCCTATCATATCGAA	GCCATGTCAAGGCAATTTCT
BoSF2395	SSR	GGTTTAATCTTTTAAGCCATAGCC	CAAGCAAAAGATGTGTCTGGA
BoSF2398	SSR	CTTTCACGCCTCGAGAGTTC	GGACATGAAGCTCTGATCGAA
BoSF2406	SSR	AAGGAGGATCACGAGGAGGT	CATGGTAGCATCGAAAGCCT
BoSF2407	SSR	CTAACAAGCAATCAACCGCA	AGCTCTTCGACGGATTCAAA
BoSF2475	SSR	TTCCAAAATCAGCCAGCTTC	AGATGGGCAACCAATCAATC
BoSF2482	SSR	GGTTTCTTGAGAATGCTCGG	TTGGCAAATAGATGCTTTGAA
BoSF2489	SSR	GAGCAGCAGAACCAAAGGAC	ATCATGACGATGACAAGCCA
BoSF2499	SSR	GAGGCTGGCTTTGTATGGAG	TCAAAACGATCCCTCAGGTC
BoSF2506	SSR	AATCTTTGGCCCTTCCCTAA	TGCAAGACTTGGCTCAACAC
BoSF2507	SSR	GGGCGTATCAGGAAGAGTGA	ATGTGTGCCTGGGAGAAAAT
BoSF2508	SSR	CGGTACGGCGAATAGAAAAA	GTCCCCGATGAAACAGTGAT
BoSF2509	SSR	TCGTCGTCATTTCTCTGTCG	CTGTAACGAAAGGCGGGTAA
BoSF2510	SSR	TGGCATTTGAGTAGTGGCCT	AGTGGCCCACACTTCCATAG
BoSF2513	SSR	CATCGTGAACGTGCTCATCT	AGGAAGTTCAGACCAAGCGA
BoSF2538	SSR	GTCCCTGTCAAAACGACAAA	GGAGCTAGGGCTTCGTTTTT
BoSF2549	SSR	ACGCTAGGCAAAGAGCAGAG	ATGCTTAGGCTTGTTGGGTG
BoSF2552	SSR	GGTGCACACCAATGACTACG	CATGAAATGCTGAGAGAAAAGG
BoSF2575	SSR	CTGCAACCGATTTGGAGTTT	CGATCTGATTGATGTGTGGC
BoSF2578	SSR	ACAATGGTGGGAGTGGAGAA	GAACAAGTCGCATTGTTTATTGA
BoSF2583	SSR	CTTCTTGCTTTGGCCTTTTG	GTGTTTGCGTGTTGGACATC
BoSF2591	SSR	CCAAAGGTGTCACGCATGTA	TAATTCGGCCTCTGTGTTCC
BoSF2604	SSR	CGAGAATCTCATCTGGCTCC	TGCATGACTAATGCTTCGGA
BoSF2607	SSR	TTTTCGTGAAGCTGATGCTG	CCAGCTTTCTTTAAAATCCCAA
BoSF2609	SSR	CTTCTCCAACAAAAGCTCGG	AGAGGACTGGTCCCCAAAGT
BoSF2616	SSR	GCACTGTCCATCTGCAACAT	CCAGTCTTGGACATGCGTTA
BoSF2624	SSR	AAAAGCGAAATGAACCAACG	TCTTCGAGAGGAGAATAAGAGTTGA
BoSF2646	SSR	GCAACATACCATTTTTGGGG	ATCTCCTTTCCAGCGAGTCA
BoSF2653	SSR	ATATGGCCCTGCAAACAGAG	CAGCTTATGGAATGCCCCT
BoSF2657	SSR	CAAAAGGCAAAGACCGAGAG	CCACTGACCTTGGATGTGTG

BoSF2666	SSR	CCTCGATGAGACGCAATTTT	CATTCAAGGTTCAGCTCCTG
BoSF2669	SSR	CGAGTTGGAGCCTACTTTGG	ACAAGCAGTGCTGCAAACAA
BoSF2670	SSR	AACGCAGAATCAGGCAAAAC	TTAGGAGTGAGCTCGGGAAG
BoSF2672	SSR	GGTCCGAGTTCCAACACAGT	CAAGGGGGTTTTGATTCCTT
BoSF2676	SSR	TGGGAGAAACATGGGAAGAG	CGCAGCTTGTTCTCTCAAAA
BoSF2702	SSR	CTTTGGCAGAATGTGGGAAT	GGTCTTAGCGCAGAGGACAA
BoSF2703	SSR	GGATCTAGGACACAAGCAGCA	TGAATCCTCCTACACCGTCC
BoSF2709	SSR	TCGTCTGGAGGAAACAACAA	AAGCGTACGTGGACCATTTC
BoSF2710	SSR	TAGAACGCAAACCAGTTCCC	CAGAGGCATTGGATGTGTTG
BoSF2723a	SSR	CGCACTAGTGACCTCGATGA	AGCTTCAAGTTCAACGGTTTG
BoSF2723b	SSR	CGCACTAGTGACCTCGATGA	AGCTTCAAGTTCAACGGTTTG
BoSF2740	SSR	GTTTGCGCCCAATAGTGTTT	TTTGCGAATCTGTACTTGTCG
BoSF2744	SSR	TCACAAGCAACGAGAACTGC	CTGGCTTGCTTCACTTTCCT
BoSF2746	SSR	GGCGAGAGAAAGAGAGAGATCA	AACGCAACTCAAAAGATGGG
BoSF2748	SSR	TGTTCACCGCATGAATGAAA	ACAATCAGATGGAAACCCCA
BoSF2751	SSR	TCCATGACTCTCGTGCTGTC	TGTGTGACGAATTCACTGGG
BoSF2754	SSR	GGCAAAATCACTTCGCAAAT	CAGACCTCGAAAAAGGCTTG
BoSF2769	SSR	CGGCCTAGACACCTCTTCTG	GAAAACGGCGATTAGTTTCA
BoSF2770	SSR	TCACCCTGTGAAAAATGCAA	GGAAACAGCCACTTCACCAT
BoSF2771	SSR	TTCAATCGCCATTGTTCAAA	TTGGGAGGATAAGAACGACG
BoSF2774	SSR	GTGATGAGAAGCTAAGGGCG	TTTTGGGAAGTTCCTCTGGA
BoSF2788	SSR	TCCAGAGCAGCGTATAGCAA	CCAAAAACACGGAGAAAGGA
BoSF2791	SSR	GGAACTCAACGAGGAAAACG	AGAAGCTTTGCTTTGCTTGC
BoSF2807	SSR	TCGGTAAACCAATCCCAAAA	GAAGGAGCTGAGCAAGGAGA
BoSF2820	SSR	GTTGCGATACGGAAGGAAAA	CAGAGTCCGTTCCCCTATGA
BoSF2821	SSR	AGTGCGTTCTCCTCGTTGTT	CGCCAATTCGTTAGGGAGTA
BoSF2828	SSR	GGCGTTGATGTTGCTCAGTA	TACACACCATTCCCTCCCAT
BoSF2838	SSR	GGGACGCAAAATTTACGCTA	CTTGCCGTGACCCAATAAGT
BoSF2852	SSR	TGCCTCATAGTTCAGGTCCC	CAGGAAACCTCACGGATGAT
BoSF2861	SSR	GCTCGTTGTTCAGCTCCTTC	CCATGATGCGTCTGGAGTAA

BoSF2872	SSR	GAACCCAAGGAAACATGCAC	TCAGAGTGTCCTCTGCCTCA
BoSF2884	SSR	CACTCCTACCACCTCGCTTC	GATTCCTCCTGCAAGTTTGG
BoSF2915	SSR	GTTGCAGCTTCAGCCATTTT	GAGCGCAATTCTCTGAAACC
BrSF0006	SSR	CATCGCTAATCAGAAAATTAGTGAAA	CGTTGGTAAGATGGGCTAACA
BrSF0009	SSR	AACCATATCAAAAGCATGGC	GAGTAGAATGAAATACTTTGTCGGC
BrSF0010	SSR	AAACGGGAAATTTAAGCGGA	GCTTCCCGGAAAGTCTTCTT
BrSF0016	SSR	CGCAACGAACTCTGTCTTCA	ACACTGCCTCAAGCGAATCT
BrSF0019	SSR	GACTTTTGTCCCTTTCCCAA	TAAGCACAAACCACACCCAA
BrSF0029	SSR	TAGGGTTTAAGGTTGTGGCG	CCAAACTAACCGAAACCGAA
BrSF0031	SSR	GCTGGCAATTTATAATTATCTTGAA	TTCCGAAATTATCGCAATGAG
BrSF0032	SSR	TGGGGCTTCCAATTTTCTAA	TGGATGTCGTCCAAGATGAA
BrSF0040	SSR	CAAGTTGGTCGAACGTGTGT	GTGATCGAACCGGTGGTTAT
BrSF0045	SSR	TCGTATGGCTAAACCAAAATGT	TTTTTCTCTTTTTAGCATTATCCCA
BrSF0049	SSR	CGTTGCAATGTTTTTCTTTTAAT	GCTGCGTTCACGTACCCTTA
BrSF0051	SSR	CATTGATGAAACTTGGGACTAGAA	CAATCAACATAAATAATACTCCATTTG
BrSF0052	SSR	GTGAAGTGAGGTTTTGGGGA	AGTGGCATACCATAGACGGG
BrSF0053	SSR	TGACTCAATCTAACATCATGGAA	TCATCATGCAGGTATGTGGG
BrSF0056	SSR	TTTGATTCATCCTATTTGCTTGTAA	AACAAAAATGGAACAAGGATAACA
BrSF0062	SSR	ACGGCTCACAGTTTGCTCTT	TCACCTTGGTTTACAGAGTCCA
BrSF0065a	SSR	ACGGATTTATGCAATTCGGA	CCAAACAATATAAATTTTGAACACC
BrSF0065b	SSR	ACGGATTTATGCAATTCGGA	CCAAACAATATAAATTTTGAACACC
BrSF0078	SSR	TTTCACATGTTTTCTTGGTCATC	CAGCGAAGAACGGAGAAGAG
BrSF0087a	SSR	TGTTTTTCCAGTAAATTGCCC	AGTTGAAGGGAAAGAGGGGA
BrSF0089	SSR	CAACATTATTTTGACCCCCA	CCTAGCTTTAACTCGGCTTCA
BrSF0090	SSR	AAAAACCAGCACAACGGAAC	AGGGAGGGATCAAGAGGATG
BrSF0091	SSR	TTCAGCCAGAAACCTCCATC	CCAAGGCCAAGCTGATAAAG
BrSF0092	SSR	TTTCCAGGGTGAAAGAAACC	ATATGCTCTCCGTCCGTTTG
BrSF0102	SSR	TAAATCTCCATGTGGTGGCA	CTTTCTTTGGTCGGGGTTTT
BrSF0112	SSR	TTCCACCCCTTGATCAACAT	CCTCAAAGGAAGAATGGGGT
BrSF0114	SSR	CACCACGAGATACAAGCAAA	TTGTTACAACAAGATGCAAATAATCA

BrSF0120	SSR	CAACTTCGGGGAAATTTGAA	CATTGTCCAAATGAAAGAAAACA
BrSF0144	SSR	CAATGAATGTGATGTTATCCGA	AAGATGTGAGGGTAAGTTTGTGG
BrSF0149	SSR	AGAGAGTGGGATCGATGGTG	TTTCTGGATCCGCAAACTAA
BrSF0153	SSR	GATGTTCATTCCGTGCAAGA	GTGGATTAATAGCCCGACCA
BrSF0154	SSR	CGGGTGGACCTAAATAGCAT	AACTGCTTTGAAAGTTAATATTTGGA
BrSF0162	SSR	AACCATGTAGTTCAGAAAGCACA	GTTAGAAATGCGCCGTCAAT
BrSF0166	SSR	GATGGAAAGTAGCTCCTGCG	AAGCAGAATTTGGGCCTTTT
BrSF0171	SSR	AATCACCGGCGTTGTTTTT	GCGGATCTCGTTGATGTTCT
BrSF0185	SSR	ATTGCACGCATCCACATAAA	TTCAAAGGATAAGGGCATCG
BrSF0193	SSR	TTGAAATTGTCCAAAACACTTGA	TGGATTTGAATAATACATGCCAA
BrSF0210	SSR	TCAAGCTTCAGCCCTTAAAAA	TGCCTTCATAAACACGCAAA
BrSF0214	SSR	CGTATAATTTTCATAGGCGACG	AGCATGCTTATGACTCTGGGA
BrSF0229	SSR	AAAAACATAAAGACAACTTTGATGAC	GAGATTTTGACAAACGACTTGAAA
BrSF0250	SSR	ATTTGCAACGGTCAAAACAA	CCATGTGAGAAAACCATCTCC
BrSF0251	SSR	GACCGCCACCAGATCTTAAA	TCAACTTGACATGTTCACTAATAGTTT
BrSF0255	SSR	CAAGTTTTTCGTGGAATGGAA	CAAAGATACAAATGACAATAAAACCA
BrSF0261	SSR	TGATGGGTCTGAATTGGGTT	TCCTCGTCGATATGGTGACA
BrSF0279	SSR	AAATTCAATGGTTAAAGTCAATGTT	CATATGCGGGCTTAATCGTT
BrSF0283	SSR	CTTGTCTCTTGTAACTTGCCCA	TGAGAACATCTGACCCACCA
BrSF0287	SSR	TGACAAACTTGTAGATAATTTCCAGTG	GAGATTATAACTTTTCCAATGTGTGC
BrSF0295	SSR	TCGTGATGACTACGGAACCA	CCAAAGTACAACATGATTTTAAGGA
BrSF0299	SSR	AAGACAATGTGGGCAGAGAA	TCTTGAGGAGTGTGTGTGTGTG
BrSF0300	SSR	CCTTCTGCGTTGCTTCTTCT	ACTTTCGTTTCTGGGGTCCT
BrSF0313	SSR	TGAGTCAAATTGGGTGACGA	TCTGGTCAAAGAGATTGCAACTAA
BrSF0331	SSR	TCTTCTCCGATTGGTTTTGC	TTTAGAGGCAATTCCGTTGG
BrSF0350	SSR	TCAAAGTATTGATTTAGTTTGATTGAA	ACGGAGGACGTTGACAAATC
BrSF0353	SSR	TTGACATTGGAGAATTGGCA	ACAAGTACGTCGGCTCAAGG
BrSF0356	SSR	ATGGCGAAGATAACGTTTGG	TAGGGATGGTGGGGAACATA
BrSF0369	SSR	TGAGGAAGATGATTGAGGGG	ACCCAAGTATACCAACGCCA
BrSF0383	SSR	ACCAAACCAAACCAAACCAA	CGATCTTTTGGTGGAAGGAA

BrSF0385	SSR	ACCAGAAGACGATTCCAAGC	GCGAAGACTGACACGACTTG
BrSF0389a	SSR	AGGGACTCAGTGCTTCCTCA	AACGATCTCGGTGGTGGTAG
BrSF0403	SSR	TTCCCCATATATCACGCCAT	TAGATCAACGGCCATGATGA
BrSF0415	SSR	TTTTCTTTCCATTCACAGCAGA	GAGAAAATCACGGAAGAAAATCC
BrSF0422	SSR	GGATCTAGAAACCCCTTCACAA	TGATCGAGATCGAGACCCTT
BrSF0425a	SSR	CTGACCCCACTACTTTCCCA	CAGAGATGAGAGACCGCACA
BrSF0425b	SSR	CTGACCCCACTACTTTCCCA	CAGAGATGAGAGACCGCACA
BrSF0427	SSR	TGCGTGGAATTGATCTGAAA	ACAGCCTGACCGTAACAACC
BrSF0436a	SSR	TTGTTTGAAGTGAGAAAGAAATCAA	TGCTAAGCTAAGAGTGAGCATGA
BrSF0436b	SSR	TTGTTTGAAGTGAGAAAGAAATCAA	TGCTAAGCTAAGAGTGAGCATGA
BrSF0446	SSR	CCGGGTATAGATCGGGTTTT	GAGAAGAGAAGTGATGCGGC
BrSF0472	SSR	AGGCCCTCTCTGTCCATTCT	CCTCCGGTGTCCATAACAAC
BrSF0480	SSR	TTCTGTCCAGCTGCAATCAC	AGCATCACTAGCTTCGCCAT
BrSF0491	SSR	GCAACCACAAAATATCGAAAA	TCATGGCTCTCACGTTTCAG
BrSF0497	SSR	CCGCCGACAGTTTCATATTT	AGTTCTAAACCATGCCCCCT
BrSF0503	SSR	GATTCGAGTCCCCATGTCAC	CACTTGGAGTGTGCAGCTTT
BrSF0519	SSR	ATATGAGCCACACGTCAGCA	TGTTGATGTACACTTACAGCTAATTTT
BrSF0520	SSR	GCTGCATCATAAACCACATGA	GAAAATTGAATGCAAATTCCAGA
BrSF0549a	SSR	GCTACTCATCTCCGGCTCAC	GTCCACCTCGTCACTACCGT
BrSF0550	SSR	TGGCAATCTTCTGCTCTTGA	AATGGCTCATTTTCCTGTGG
BrSF0552	SSR	ACCCAACTCTAACGTGTGGC	AAAGCAGCAACCAAGCAAAT
BrSF0560	SSR	GCCAAAAGAGAAAGCGAATG	GAAGATCTGGTGCGGAAGAG
BrSF0564	SSR	CGGCAGCTTGTTAAGGCTAC	GCCTTGTTCCATCCTCCATA
BrSF0570	SSR	TTGACGGTTTACCGGATTTC	GAGCACCATTGCCAATTTTT
BrSF0606	SSR	TCGGGCAACTAAAAGGCTTA	TGGGGAGCTATCATCTTTGT
BrSF0615b	SSR	GAAGAGCAGGAAGTTTTGGC	TCTTTTTCGACAGCATATTGAGA
BrSF0618	SSR	TTGCGTATAAAACGTGCCTG	CCGTGCTGATCAATCAAAGA
BrSF0620	SSR	AAATCCCGCATTTTTACACG	TTGTATTCGTTTCAACTTTTAAGCA
BrSF0642a	SSR	ACGTGGACTGGGAAAAGATG	CACCTGTTCCCCAAAAGAGA
BrSF0643	SSR	ATGCTTTGTGTTGCCTGTTG	AATGCGAATGTCATATGGTGA

BrSF0645	SSR	TGGGTTAGTCAAAAAGGGGC	CATCCTTCCCAAGTCCTAACA
BrSF0646	SSR	ATAACCAGCGGTGCCAATAC	GGAGAAAGAATGGCATCGAG
BrSF0661	SSR	CCCCTGCAATTTGTAACCAC	CCCTTTTGAACAAGGTACGC
BrSF0663	SSR	ACCGAAACATGTGGCTCCTA	CGAGAAACATCATCCAATTCA
BrSF0665	SSR	CGCCATGACTTGCTGTTAGA	CCATAAATAGAGGGACGCGA
BrSF0679	SSR	ATCCAACGGCTACCCTTCTT	ATCTCTCCGGCCAAATCTTC
BrSF0680	SSR	TTTTACAACCGTGCACACAAA	AAATCCGATCCCCAACATCT
BrSF0682	SSR	TGCTAATGCTCCCGTATTCA	CGGTTTTCCTCTCTTCTCGTC
BrSF0689	SSR	TTGCCATTTTGAACATTAGGAA	TGAGCCTCTCTGAGTCTCGAA
BrSF0695	SSR	CATCATGTGCCTTGTCGAAC	ATTAAACCCCCACCCAAAGA
BrSF0696	SSR	GAATGATTTTCAATTTTTAGGTTTCA	TGATTGACCGACTGATTCAAA
BrSF0702	SSR	TGCCTTGTGCTCCATAAACA	ACGCAAGCCAAAAAGAAGAA
BrSF0703	SSR	TGCACCACACATAGCAAACA	TGAGTTGTGGGAATGCAAAA
BrSF0705	SSR	TGAAAACACTGCGATTGAGC	TGTCAAATCAGACAGCCTGG
BrSF0706	SSR	CGAGGGAAATACCCATACCA	GAGAAGATGTTCCCGAGGTG
BrSF0708	SSR	GATTTCTCAGAGCCGAGCTG	CTCTAACGTCTAGGGGCACG
BrSF0712	SSR	CCCACTGAGAAAATGAAGGAA	CGTTTCCAAAGCCAAATGTT
BrSF0719	SSR	CGCCGTGGATACTTCATTTT	CTTCCACGCACTGTAAAGCA
BrSF0726	SSR	AATCGTCAATGATCATCGGTT	ATGCACACGCATTATCCAAA
BrSF0741	SSR	CGTCTCGAAGAAGCAAAAGG	AGATTCCTCCGGTGATGTTG
BrSF0743	SSR	TCACGTCATCTCCATCATCAA	TGGCATGATTACCAATGACTG
BrSF0750	SSR	GGGAGAAGGAAGAGCAAAAGA	TCGTCGCTAAAATGAAATTGA
BrSF0754	SSR	GCAAATCAAGCAAGGTTGAAG	TCAACTTTTTATGAATCCTTCTTTG
BrSF0755	SSR	CTTCAAGAAACGATATCGGGA	GGTTTCAATTCGTCGGAAAT
BrSF0784	SSR	TTGGGGACTTGTCACATCAG	GGACCCGTGGATAAAAGGAT
BrSF0789	SSR	TGGTTATTTCATGGTGGCCT	AACCTCCAAAGTCAAACCTTTTT
BrSF0811	SSR	AAAAGGAAGGGCAAAGTGGT	TTTTGCCATTCGTGTCGTAA
BrSF0819	SSR	CGAAATTAAATCGTACATGCATAA	TTTGTGAAACAAAACGAGCG
BrSF0820	SSR	TGGGTTTGTGAAAATATGGTGA	TGTAAAGTTGTGCAAGGATTGTG
BrSF0821	SSR	GATGCGCTCACAATTCTTCA	CCTATTGACCCCACACCACT

BrSF0822	SSR	GGGCAGGCTAACCCCTAGTA	GAAGACGACGACATCGGTTTA
BrSF0836	SSR	AATCGTCGAGTTTCACCGTC	CTTCCCTGAGACAAGCGAAC
BrSF0845a	SSR	CACCAGAGAGAACGCAATCA	TGCTCCTGATGCATTAACCA
BrSF0845b	SSR	CACCAGAGAGAACGCAATCA	TGCTCCTGATGCATTAACCA
BrSF0849	SSR	TTGCTCTTTGAGGGTTTTGG	CGCCTCTTTGCCCTAAAGTA
BrSF0858a	SSR	CGAAGTTCTCCGGTAACCAA	CTTCGAACTCGACCAGAAGG
BrSF0858b	SSR	CGAAGTTCTCCGGTAACCAA	CTTCGAACTCGACCAGAAGG
BrSF0860	SSR	GATGCCAGTACATGACGACG	ATTCCCCACATTTTCCATCA
BrSF0869	SSR	TTCACTGCTCGTGTAATCGC	TTTGGTGGTGTTGCTTGTGT
BrSF0898a	SSR	CCAACAACGTTCGTGTTCAA	TTTACCGTACCGGAATCCAA
BrSF0916a	SSR	CGGGTCCATGGTCTATAACTTC	CCCACACACAGAGTCCCCTA
BrSF0918	SSR	GGGTGAATCTCAGTCAGGGA	CCCTCGTTCACGACTTCTCT
BrSF0942	SSR	TGACATTCGCATCAGATTTGT	TTCCTCCATCGTCTTTGTCC
BrSF0949	SSR	GCATCAAACGCACACCAATA	TCTGCCCCTGTCTCTCTGTT
BrSF0952	SSR	TTGGTTCAAATTATTTTTAATGGAAG	AAACCCCAGATTTTCATCATTTT
BrSF0958	SSR	TGCGCAACTAGATAGAGCATGT	CACCATGACCCTCCTGATTT
BrSF0969	SSR	CAGAAAAACACGTTTTGGCA	AATTTGGGATCGGTTTAGGG
BrSF0972	SSR	AATGGCGACGATTCTGTTTC	TCATTATTTTGGATAATTTAGACCCTT
BrSF0973	SSR	ACGCAGCTTACAGTCGTGTG	TCCAAGCAGTTTCTTTGCAT
BrSF0975a	SSR	TGGTTTTCTCTTTTCATTTCTTTCTAT	TTTGAAAATGTTGAAAAAGAAGTTG
BrSF0982	SSR	GAGACCGTGTGTCCTCTCGT	GGTGGAAACAATAAAGGCCA
BrSF0998	SSR	GTGATTGGTTGGCACATGAC	GTGATGGTACGGCTTAGGCT
BrSF1002	SSR	TTTTCAATGTGAGATTTCTTTCAA	AAGATGCAAATGATCATAAAATCG
BrSF1008	SSR	TCCCGTCAGATAGTGTTCTTTT	TTGGTTTATATCTTTTTGGCTTATGA
BrSF1031b	SSR	AGAAGACTCGAAGGGGAAGG	CAAACTCCAACCACGAACCT
BrSF1032	SSR	CCGCGTTTCTGAACCTAAAA	GCAAAATATGCCGGACTCAC
BrSF1044	SSR	TCAAACGATTACCAATCTAAAAACA	TCAAATTAATATGTTGGATGGCA
BrSF1049	SSR	AACTCAGTAAACCCCAAAAATAAA	TTTCCTATGCTCATGCAAAGA
BrSF46-167	SSR	TCGGCTAGAATGGCTACAAGA	TGCCCATTCGTGTATGTTTG
BrSF46-176a	SSR	ATCACCATTGTGTTATTTCTTCAG	GGATCAAATGGATTTAGCGG

BrSF46-176b	SSR	ATCACCATTGTGTTATTTCTTCAG	GGATCAAATGGATTTAGCGG
BrSF46-177	SSR	AGCTGGTACATGATTGCGTG	CAACTCTGCCATTCACATGC
BrSF46-186	SSR	GCCAAAGTGCATCCCATCTA	CCTTTGGAAAGTCGAGAGACC
BrSF46-22	SSR	TCGTGTAAGTGAGAGGAGAGTAAAGA	AGCTGGTGCAGTATGTCAGC
BrSF46-238	SSR	TTGCGAGATTATTTTTAGATATGTGC	GAAAGCGTGAATCAAAAACG
BrSF46-291	SSR	CCACGTCTGAGGTGAGGTTT	CTTTTCTTCAATGCCACCGT
BrSF46-306	SSR	ATGATCATGTGGACCGTTGA	TGATTGTTATTCCCAACTTTTTAGG
BrSF47-10	SSR	ATCAATTTTTCCGAGACCCC	AAGCTGGTGAACTCGTTTCG
BrSF47-234	SSR	CGGTTCATGTTTTGATTTGG	TATGGGATGTCCGAAGGTGT
BrSF47-277	SSR	TGATGACACGTGACTACACTCAA	ATCCAGGCGAAACATCGTAT
BrSF47-382a	SSR	GACTGTTGTTCTTCCTCGGG	TCCACAGCGAGAGATACACG
BrSF47-389	SSR	AAGAGGAGCATCATGGGTGT	CACGGTTGATAATAACGCGA
BrSF47-54	SSR	GAGTTGAAACCCCAAGCAAG	GCGAAACAGAGGCAATAAGG
BrSF53-712	SSR	ACCCCATTGGGTCACAATTA	ACCATGAGGAGGCAGAGAGA
BrSF54-590	SSR	ACGGACGTCTTCTCCTCTGA	AACGGGATGGAATGTTATGG
BrSF6-104	SSR	AAGATCTCTCAAATGTACGCCA	TCTCAAACCGAAGACAAAACAA
BrSF6-2245	SSR	GAGGAAAGAGTTGCCGTTTG	CTGCTCTCAAACCACTGCAC
BrSF6-2380	SSR	TCACCAGCTCAACTGTTTGG	AACGTTCAAGCGGAATTGTC
BrSF6-2389	SSR	TCAACTTGACATGTTCACTAATAGTTT	CAATAGACACGGAAATGGGC
BrSF6-2413	SSR	TTTGCTTGGAATCAAAATTCTTT	AGCTTCAAATCAGCGGAGAA
BrSF6-2562	SSR	TGTTTCCCCTATATATTTATTTGTGGT	TCAAAATAACAACCCATTCCA
BrSF77-118	SSR	CACTCTCCCGCTGTAGGAAG	GAAACCAAAGCAATCCATCAA
BrSF82-562	SSR	GCGAGATCCAACCAAAAACA	GTGGCCCAGGTTTAAAATCA
ns027b	SNP	CTTGCTGTGAGTTTGTGGGTCCGA	CGCCGCTGCTCCAACGCTAG
ns033	SNP	CGCTGCATCACCCCGCATCA	TGACCCTGCTCCAGCCTAGTCTCAAA
ns059	SNP	CACGGAAAGGCAGACGCGGAG	AGAGATTCTTACCACAGATGTGACAGGTACGC
ns067	SNP	ACACGTGCAAAACGAGTTACTATAATAGTTTTATG	ACAGTTTTTGGATTGGAAGAAAGAAGATAAAATGG
ns085	SNP	GGTGTTTGAACAGTCTGCATTTTCAGGAG	CGGTTTAAAATTTTGGTTAATTCGGTTAGTTCAGTT
ns104	SNP	TCCTGCTTCCTTACAGCCTCAGTGTTACC	ATGGAAATCAGAGCAAAAACAGAAACTTTGGT
ns120	SNP	CTTCAGTCATTGCAGCTTATTTCTCTGATATGTTC	TGAAGAACAGGTTATAGCCTTAGCGATAGGAGTTT

ns123	SNP	ATCAAAATCCCAAATTAAACCAAAATATTCTCCAGA	AAACCGGTTTAGGCCTTTTCCCTCC
ns126	SNP	CAGATTCAGGCAATATGGATTATGGCATC	GTGTGTAGAGTCCGACCCGGTTCAAA
ns155	SNP	CTCTAAACCGTTCAGAATAGCGTCGAGATATCTT	ACGATGGGGAACGTCGACAACG
ns156	SNP	ACTAAGCTTCACTAGGTAAATCTAATCTATGTGGGT	GAATCACGCGAGTGAAGAAGCTGCTAAG
ns158	SNP	AAAGCACGAACGTCAAAGCCGTTCTT	TGGTGCTAGAAAGCAACCGTCTTTCG
ns160	SNP	CACTTCTTTTTGTGGGTTGTTAAATTGCTTCC	AAACACCATTTTACCTTCTCCTCCCTTGTTTAAG
ns161	SNP	CCACCCTTTCATCAGTGACATGACCG	AGTTTCCATCGGTAACCCTAATGGTTTGTTC
ns162	SNP	GCATTCGTTGAACTTGCTTTGAGATGTATTAGC	GCTGCGACATCATTTTTAAGAGATCCTCTGT
ns163	SNP	CCGGAAGGCTCGCAAATGCTC	GCCACCGTTCCCGATCCCAT
ns164	SNP	CAAATCCTTAACAGAAAACCAAACCGGAGTTT	TAACGGCGACGACGGTTGACG
ns165	SNP	CATGAGAGAGAATAACTGAGAAACTGCAACCAT	TTCCCCTTGGATCCACAGATGGC
ns166	SNP	TGCAACTAACTCATCAGCACTTAAGACTGGC	TTCCCCTTGGATCCACAGATGGC
ns167	SNP	CATCAGCACTTAAGACTAGCGACTGCTGATAAC	AACTTGACGAGGACGGGTGAGATAATGAG
ns168	SNP	CACACTAATGCACAATCATGTAACGCAGGAT	TTGAATTATTTGGGAGCGCTGGATGA
ns211	SNP	GCAAAGGCCTCTTCTGCAACTGTTACT	GGTTGGGTGAGTTGATATTGTTTTATCTGATCTGT
ns213	SNP	GCTGCACCATGGAGGTCTGATGTAGATAGT	GCTCCAACAAGTCCAGACAGGGTCC
ns218	SNP	TCTTCACTTGGGACCAAACAAATGGAAT	GAAACTTACCGCTACTGTTGCGTGAGGA
ns231	SNP	CCAAAGTTAATCAAATCATGCCTTGTGTATTTAACT	AAATTGAACGCTTTGAGGATTTGGTTACCTACTAG
ns258	SNP	AACCTTAGCCTAGCAAGCACAATAGAGATGAAAT	GCTTCAGTATGGGTAGATTCAATCGATATCTTTGTT
ns262	SNP	CAGTGTTTTAACAAGCGAAAGGACGCAAATT	TTACCATACCAGCCTTCACCATTCCTGA
ns314	SNP	ATCGATCAAACTACAAGGAGTTGCTAATTCCAGT	AACATATGGGCTTACACAGAGCATGGTTAATATACA
ns326	SNP	GAGTCCAGCCCGAATGTTTCAGAACT	CAACAGCTTCACCTGACGCAGAACC
ns337	SNP	TGCATTACAGTTGCTCATCTGCGCTTAAT	TGACTAAAGCTCTCTATGGTCTTAAACAGGCTCC
ns347	SNP	CCATATCACGTGCCGTTGAAGCAAAT	CTTGGCTTGCTCAGTTACAGGTGTGTGTT

Table S3 CHISQ test for segregation of 803 markers on the 19 linkage groups of BnaZNF$_2$ genetic map.

Linkage group	Marker_name	Position	A	B	H	C	D	-	Px^2	Toward
A01	BrGMS0081b	0.0	0	119	0	0	52	8	2.E-41	No.73290
A01	niab097a	6.1	0	98	0	0	77	4	3.E-21	No.73290
A01	BoSF2770	8.8	0	121	0	0	55	3	6.E-41	No.73290
A01	BnEMS0886	13.3	13	60	14	0	0	92	2.E-20	No.73290
A01	niab106	27.2	41	0	0	126	0	12	9.E-01	
A01	niab071	37.5	48	0	0	131	0	0	6.E-01	
A01	CNU139a	39.2	42	0	0	133	0	4	8.E-01	
A01	CNU142c	41.5	44	0	0	135	0	0	9.E-01	
A01	BrSF0019	43.4	43	45	89	0	0	2	1.E+00	
A01	BoSF0224b	44.3	41	0	0	137	0	1	5.E-01	
A01	BoSF0302	44.8	0	42	0	0	135	2	7.E-01	
A01	BoSF1331a	46.8	43	0	0	131	0	5	9.E-01	
A01	CB10081	50.4	41	42	93	0	0	3	7.E-01	
A01	BRMS037	50.4	39	0	0	133	0	7	5.E-01	
A01	niab113a	52.0	41	0	0	138	0	0	5.E-01	
A01	BrSF0403	56.5	42	41	89	0	0	7	9.E-01	
A01	BoSF2131	59.4	50	42	86	0	0	1	6.E-01	
A01	CB10099a	69.1	44	0	0	120	0	15	6.E-01	
A01	BrSF0016	72.2	0	37	0	0	141	1	2.E-01	
A01	BrSF0446	82.7	49	37	92	0	0	1	4.E-01	
A01	CB10189	83.5	49	40	87	0	0	3	6.E-01	
A01	BoSF0764	87.5	0	39	0	0	137	3	4.E-01	
A01	CNU132b	89.8	28	20	41	0	0	90	4.E-01	
A01	BoSF0503	89.8	42	37	98	0	0	2	3.E-01	
A01	Ra2G05	90.7	40	36	95	0	0	8	3.E-01	
A01	BoSF1659	91.4	42	0	0	136	0	1	7.E-01	
A01	BrSF0702	93.3	39	36	102	0	0	2	1.E-01	
A01	SA63	93.8	37	37	105	0	0	0	7.E-02	
A01	BrSF0703	94.1	36	37	106	0	0	0	5.E-02	
A01	BrSF0706	95.0	0	37	0	0	140	2	2.E-01	
A01	CNU068	98.1	37	0	0	142	0	0	2.E-01	
A01	BrSF0010	98.1	38	71	69	0	0	1	2.E-05	No.73290
A01	BrSF0009	98.5	36	0	0	143	0	0	1.E-01	
A01	BoSF0050a	99.6	40	0	0	136	0	3	5.E-01	
A01	BrSF0006	102.1	36	0	0	140	0	3	2.E-01	
A01	CNU397	103.2	42	0	0	137	0	0	6.E-01	
A01	BrSF1002	104.7	0	47	0	0	130	2	6.E-01	
A01	TFZIP23	105.8	0	45	0	0	134	0	1.E+00	
A01	sN3523	106.9	41	45	91	0	0	2	9.E-01	
A01	BrSF0982	113.9	42	0	0	128	0	9	9.E-01	
A01	BoSF0911	119.1	0	48	0	0	130	1	5.E-01	
A01	CNU474	122.1	42	0	0	137	0	0	6.E-01	
A01	CNU388	123.5	0	41	0	0	137	1	5.E-01	

A01	BoSF1939	124.1	0	33	0	0	129	17	2.E-01	
A01	BoSF2123	124.4	39	0	0	140	0	0	3.E-01	
A01	BoSF0197	125.0	38	0	0	139	0	2	3.E-01	
A01	BrSF0973	125.5	0	38	0	0	134	7	4.E-01	
A01	Na14F11b	126.5	45	0	0	124	0	10	6.E-01	
A01	BrSF0949	128.8	30	32	104	0	0	13	5.E-03	
A02	niab025	0.0	53	0	0	122	0	4	1.E-01	
A02	BoSF2549	5.7	44	0	0	130	0	5	9.E-01	
A02	sR94102	6.1	0	20	0	0	66	93	7.E-01	
A02	BrSF0051	9.4	55	36	87	0	0	1	1.E-01	
A02	BrGMS0511	11.1	42	0	0	117	0	20	7.E-01	
A02	BrSF0065a	11.5	0	37	0	0	140	2	2.E-01	
A02	BoSF0239	12.8	48	38	92	0	0	1	5.E-01	
A02	BRAS083	18.4	0	38	0	0	140	1	3.E-01	
A02	BrSF0665	19.1	50	0	0	127	0	2	3.E-01	
A02	MR052	20.3	49	0	0	128	0	2	4.E-01	
A02	BRAS037	22.4	48	44	83	0	0	4	7.E-01	
A02	BrSF0062	23.6	50	0	0	129	0	0	4.E-01	
A02	BrSF0661	24.9	45	45	85	0	0	4	9.E-01	
A02	sN3761	26.2	46	0	0	123	0	10	5.E-01	
A02	BoSF2224	27.2	48	42	85	0	0	4	8.E-01	
A02	BrSF0663	28.0	45	0	0	131	0	3	9.E-01	
A02	sR6293	29.9	49	47	83	0	0	0	6.E-01	
A02	BrSF0045	30.5	50	44	75	0	0	10	3.E-01	
A02	BoSF0329	31.6	46	0	0	133	0	0	8.E-01	
A02	BrSF0750	33.5	46	42	91	0	0	0	9.E-01	
A02	BrSF0053	34.0	46	42	90	0	0	1	9.E-01	
A02	BrSF0052	34.2	48	0	0	129	0	2	5.E-01	
A02	BrSF0754	34.6	48	41	90	0	0	0	8.E-01	
A02	BrSF0755	36.2	50	43	86	0	0	0	7.E-01	
A02	BrSF0049	38.2	52	0	0	126	0	1	2.E-01	
A02	CNU046	41.9	53	42	82	0	0	2	3.E-01	
A02	BrSF0031	43.1	51	41	85	0	0	2	5.E-01	
A02	BoSF1714	43.6	39	0	0	96	0	44	3.E-01	
A02	BoSF2100	45.9	46	0	0	128	0	5	7.E-01	
A02	BrSF0570	47.2	48	0	0	128	0	3	4.E-01	
A02	BrSF0056	47.8	49	42	87	0	0	1	7.E-01	
A02	BrSF0679	48.5	0	36	0	0	140	3	2.E-01	
A02	IFLP018	51.2	0	47	0	0	132	0	7.E-01	
A02	BrSF0682	52.1	47	0	0	128	0	4	6.E-01	
A02	BoSF0216	53.6	0	40	0	0	136	3	5.E-01	
A02	FITO255	55.2	51	0	0	121	0	7	2.E-01	
A02	BoSF0926c	56.2	52	39	87	0	0	1	4.E-01	
A02	BrSF0680	58.1	0	19	0	0	70	90	4.E-01	
A02	BoSF1924	58.5	52	36	90	0	0	1	2.E-01	
A02	BrSF1044	60.6	54	0	0	125	0	0	1.E-01	

A02	BoSF2657	60.7	51	36	84	0	0	8	3.E-01	
A02	BrSF0696	60.9	0	35	0	0	144	0	9.E-02	
A02	BrSF0695	61.1	52	36	91	0	0	0	2.E-01	
A02	BrSF0032	61.3	53	0	0	125	0	1	1.E-01	
A02	BrSF0472	61.3	53	36	90	0	0	0	2.E-01	
A02	BrSF0836	61.4	54	36	89	0	0	0	2.E-01	
A02	CB10628	61.7	52	36	87	0	0	4	2.E-01	
A02	BrSF0520	61.8	52	36	89	0	0	2	2.E-01	
A02	BrSF0519	62.2	51	37	90	0	0	1	3.E-01	
A02	BoSF2324	63.2	52	33	82	0	0	12	1.E-01	
A02	BnEMS0401	65.5	0	37	0	0	141	1	2.E-01	
A02	BrSF0689	67.3	58	39	82	0	0	0	7.E-02	
A02	BoSF2214	79.6	52	33	93	0	0	1	1.E-01	
A02	Na14B05	82.9	51	0	0	127	0	1	3.E-01	
A03	niab115a	0.0	60	31	87	0	0	1	8.E-03	
A03	BoSF1704	6.2	49	30	95	0	0	5	6.E-02	
A03	BrGMS0642	12.1	44	0	0	104	0	31	2.E-01	
A03	BrSF0422	12.8	50	35	94	0	0	0	2.E-01	
A03	BrSF0427	15.6	44	36	78	0	0	21	7.E-01	
A03	BrSF0350	16.1	52	35	91	0	0	1	2.E-01	
A03	CB10036	16.5	52	34	88	0	0	5	2.E-01	
A03	BoSF2852	17.1	52	31	94	0	0	2	6.E-02	
A03	BrSF0029	18.6	28	22	38	0	0	91	3.E-01	
A03	BoSF2392a	20.6	0	40	0	0	137	2	5.E-01	
A03	TFZIP19	21.1	0	41	0	0	135	3	6.E-01	
A03	BrSF0090	21.8	49	47	80	0	0	3	5.E-01	
A03	KS50980	21.8	56	40	82	0	0	1	1.E-01	
A03	sR6688a	23.3	49	38	76	0	0	16	3.E-01	
A03	BrSF0089	24.2	54	40	84	0	0	1	3.E-01	
A03	BrSF0091	25.4	0	40	0	0	139	0	4.E-01	
A03	BrSF0845a	25.7	54	0	0	123	0	2	9.E-02	
A03	BrSF0087a	26.3	53	0	0	125	0	1	1.E-01	
A03	BoSF2807	26.8	55	42	80	0	0	2	2.E-01	
A03	BoSF0546	28.8	0	42	0	0	135	2	7.E-01	
A03	BoSF0560	30.9	0	44	0	0	134	1	9.E-01	
A03	BnEMS0894	32.2	55	40	80	0	0	4	1.E-01	
A03	IGF2134f2	33.5	52	0	0	124	0	3	2.E-01	
A03	BrSF0092	35.0	49	45	83	0	0	2	6.E-01	
A03	CNU253	37.0	0	47	0	0	132	0	7.E-01	
A03	Na12E02a	38.7	46	43	88	0	0	2	9.E-01	
A03	Ol11B05	38.7	47	43	88	0	0	1	9.E-01	
A03	BrSF0300	39.3	48	43	84	0	0	4	8.E-01	
A03	BoSF0415b	40.2	0	42	0	0	130	7	9.E-01	
A03	BoSF2277	40.5	47	0	0	128	0	4	6.E-01	
A03	CNU288	44.1	50	43	86	0	0	0	7.E-01	
A03	BrSF0114	44.9	48	0	0	130	0	1	5.E-01	

A03	BoSF0664	45.2	0	45	0	0	133	1	9.E-01	
A03	BoSF2744	48.1	41	48	87	0	0	3	7.E-01	
A03	CNU466	48.9	0	42	0	0	122	15	9.E-01	
A03	BrSF0078	49.8	0	47	0	0	131	1	7.E-01	
A03	BoSF1709	50.8	45	47	84	0	0	3	8.E-01	
A03	BoSF2220	51.3	50	0	0	121	0	8	2.E-01	
A03	BoSF2915	52.8	43	48	87	0	0	1	8.E-01	
A03	CB10329	55.4	45	48	83	0	0	3	7.E-01	
A03	BoSF1715	55.4	0	49	0	0	126	4	4.E-01	
A03	BrSF0295	55.9	0	50	0	0	125	4	3.E-01	
A03	BoSF1886	57.5	45	54	78	0	0	2	2.E-01	
A03	CNU098	62.1	39	0	0	140	0	0	3.E-01	
A03	BoSF1678	62.1	39	0	0	139	0	1	3.E-01	
A03	CNU259	64.0	0	54	0	0	124	1	1.E-01	
A03	FITO285	66.5	38	43	96	0	0	2	5.E-01	
A03	BoSF1651	67.0	39	45	93	0	0	2	6.E-01	
A03	BrSF0102	68.4	37	46	95	0	0	1	4.E-01	
A03	CNU422	69.8	32	45	94	0	0	8	2.E-01	
A03	Na14G10	70.6	35	46	95	0	0	3	3.E-01	
A03	Ol11G11	70.6	31	49	88	0	0	11	1.E-01	
A03	sN11722	73.6	34	49	94	0	0	2	3.E-01	
A03	CB10114	73.6	39	0	0	140	0	0	3.E-01	
A03	BoSF0598	73.7	35	0	0	141	0	3	1.E-01	
A03	BoSF2828	76.1	38	0	0	131	0	10	5.E-01	
A03	BoSF1085	98.9	0	46	0	0	133	0	8.E-01	
A03	CNU371	100.4	0	43	0	0	129	7	1.E+00	
A03	CNU223	101.4	36	45	98	0	0	0	3.E-01	
A03	BoSF2145	104.1	39	0	0	140	0	0	3.E-01	
A03	sR11644	105.8	35	53	84	0	0	7	1.E-01	
A03	BoSF0346	105.9	0	48	0	0	131	0	6.E-01	
A03	BoSF0393	105.9	39	0	0	140	0	0	3.E-01	
A03	MR181	109.8	0	47	0	0	130	2	6.E-01	
A03	BoGMS1071	110.6	0	45	0	0	125	9	7.E-01	
A03	KS40432	111.8	36	45	96	0	0	2	3.E-01	
A03	BnGMS0616	114.4	38	46	87	0	0	8	7.E-01	
A03	BoSF1919	116.0	40	0	0	126	0	13	8.E-01	
A03	BrSF0708	118.3	39	0	0	136	0	4	4.E-01	
A03	BoSF1779	122.0	36	0	0	127	0	16	4.E-01	
A03	CB10425	126.4	38	50	85	0	0	6	4.E-01	
A03	BrSF0918	129.1	43	0	0	132	0	4	9.E-01	
A04	BrSF0564	0.0	36	39	83	0	0	21	8.E-01	
A04	BoSF2609	1.8	0	45	0	0	134	0	1.E+00	
A04	BrSF0560	5.4	46	42	84	0	0	7	9.E-01	
A04	BrSF0112	6.6	48	45	86	0	0	0	8.E-01	
A04	BoSF0336a	11.2	0	49	0	0	130	0	5.E-01	
A04	IFLP014	13.4	45	44	90	0	0	0	1.E+00	

A04	BnGMS0115	14.9	45	44	88	0	0	2	1.E+00	
A04	BrSF1049	17.3	45	0	0	134	0	0	1.E+00	
A04	BrSF1032	18.0	0	39	0	0	140	0	3.E-01	
A04	niab143	19.1	20	0	0	70	0	89	5.E-01	
A04	BoSF1125	20.4	0	44	0	0	135	0	9.E-01	
A04	sN2025	24.6	0	41	0	0	126	12	9.E-01	
A04	CNU246	33.3	44	44	86	0	0	5	1.E+00	
A04	BoSF1161	35.1	43	50	86	0	0	0	7.E-01	
A04	CNU039	36.1	44	0	0	129	0	6	9.E-01	
A04	BrSF0726	47.0	44	51	84	0	0	0	5.E-01	
A04	BoSF0415a	48.4	40	49	86	0	0	4	6.E-01	
A04	BoSF0482	50.1	40	0	0	139	0	0	4.E-01	
A04	BrSF0719	50.6	0	58	0	0	118	3	1.E-02	
A04	BoSF1476	52.1	0	56	0	0	123	0	5.E-02	
A04	BrSF0646	54.8	35	54	88	0	0	2	1.E-01	
A04	BrSF0120	56.3	0	54	0	0	123	2	9.E-02	
A04	BoSF0231	56.8	37	56	86	0	0	0	1.E-01	
A04	BrSF0645	57.7	33	49	75	0	0	22	2.E-01	
A04	IFLP036	59.7	34	0	0	143	0	2	8.E-02	
A04	BoSF1555	60.1	0	56	0	0	122	1	5.E-02	
A04	BoSF1642	62.5	0	54	0	0	122	3	8.E-02	
A04	BrSF0643	63.2	37	0	0	124	0	18	6.E-01	
A04	BrSF0642a	64.4	25	25	33	0	0	96	2.E-01	
A04	BoSF0841b	68.3	34	0	0	143	0	2	8.E-02	
A04	BoSF2769	70.6	0	49	0	0	125	5	3.E-01	
A04	BrSF0969	79.4	25	0	0	152	0	2	8.E-04	No.73290
A04	BrSF0972	87.1	23	86	52	0	0	18	8.E-16	No.73290
A05	BoSF1666	0.0	39	46	90	0	0	4	7.E-01	
A05	Na14G02	3.7	41	29	102	0	0	7	2.E-02	
A05	BrSF0712	4.8	42	29	102	0	0	6	2.E-02	
A05	ns213	11.3	46	0	0	133	0	0	8.E-01	
A05	ns218	12.1	47	0	0	132	0	0	7.E-01	
A05	ns231	12.3	0	35	0	0	127	17	3.E-01	
A05	BoSF2141	14.4	44	0	0	134	0	1	9.E-01	
A05	BrSF0975a	20.7	43	37	97	0	0	2	4.E-01	
A05	BrSF0942	20.8	42	0	0	129	0	8	9.E-01	
A05	CNU398	20.8	59	0	0	119	0	1	1.E-02	
A05	BoSF1098	21.2	43	0	0	136	0	0	8.E-01	
A05	Na12E01a	22.8	40	40	95	0	0	4	5.E-01	
A05	BrSF0620	23.8	32	43	101	0	0	3	7.E-02	
A05	BnGMS0293	24.5	43	44	89	0	0	3	1.E+00	
A05	BrSF0952	24.5	44	41	92	0	0	2	8.E-01	
A05	CNU029	24.7	44	44	87	0	0	4	1.E+00	
A05	BrSF0550	25.1	44	45	89	0	0	1	1.E+00	
A05	BrSF1031b	26.5	0	40	0	0	134	5	5.E-01	
A05	BrSF0849	26.6	42	42	87	0	0	8	1.E+00	

A05	ns211	28.1	49	0	0	130	0	0	5.E-01	
A05	BrSF0618	29.9	42	41	96	0	0	0	6.E-01	
A05	BrGMS0293	34.2	43	42	91	0	0	3	9.E-01	
A05	BoSF1230	37.7	23	19	47	0	0	90	7.E-01	
A05	IFLP039	42.3	49	39	87	0	0	4	6.E-01	
A05	BrSF0497	45.7	43	38	94	0	0	4	5.E-01	
A05	BoSF0582	46.0	47	36	95	0	0	1	3.E-01	
A05	BoSF0259	46.1	48	0	0	131	0	0	6.E-01	
A05	BoSF1749	46.6	45	0	0	130	0	4	8.E-01	
A05	CNU268	49.2	0	34	0	0	144	1	7.E-02	
A05	BoSF2746	50.6	0	36	0	0	139	4	2.E-01	
A05	BoSF2030	51.3	0	34	0	0	136	9	1.E-01	
A05	BrSF0491	52.5	48	35	92	0	0	4	3.E-01	
A05	snap1047a	55.6	51	0	0	125	0	3	2.E-01	
A06	BoSF0784	0.0	59	32	87	0	0	1	2.E-02	
A06	BrSF0149	1.1	56	33	90	0	0	0	5.E-02	
A06	BoSF2253	2.0	58	32	84	0	0	5	2.E-02	
A06	BoSF0869	4.8	60	33	84	0	0	2	1.E-02	
A06	BoSF0455c	5.7	0	33	0	0	143	3	6.E-02	
A06	BrSF0860	7.4	52	0	0	109	0	18	3.E-02	
A06	CB10143	9.3	55	35	84	0	0	5	9.E-02	
A06	IFLP001	12.1	56	36	86	0	0	1	1.E-01	
A06	BoSF1212b	13.5	0	19	0	0	70	90	4.E-01	
A06	BrSF0741	17.7	0	39	0	0	140	0	3.E-01	
A06	BoSF1521	19.7	51	37	91	0	0	0	3.E-01	
A06	BrSF0743	21.4	52	37	88	0	0	2	3.E-01	
A06	BoSF2583	25.7	44	41	91	0	0	3	9.E-01	
A06	BoSF2360	29.5	48	41	85	0	0	5	7.E-01	
A06	BoSF0022a	30.2	45	45	88	0	0	1	1.E+00	
A06	BRAS052	34.2	48	56	72	0	0	3	4.E-02	
A06	BrSF0171	39.3	47	0	0	131	0	1	7.E-01	
A06	BoSF1990	41.4	45	45	86	0	0	3	1.E+00	
A06	BoSF1752	42.8	41	0	0	133	0	5	7.E-01	
A06	BnGMS0480	46.5	39	44	95	0	0	1	6.E-01	
A06	CNU050	47.1	40	0	0	135	0	4	5.E-01	
A06	IGF1653c	47.3	23	22	41	0	0	93	9.E-01	
A06	CNU149	47.6	41	46	92	0	0	0	8.E-01	
A06	CB10006	48.1	42	0	0	137	0	0	6.E-01	
A06	BrSF0858b	48.8	0	47	0	0	121	11	4.E-01	
A06	BrSF0789	52.5	42	52	84	0	0	1	4.E-01	
A06	ENA23	54.9	0	50	0	0	123	6	2.E-01	
A06	BoSF2407	55.5	0	52	0	0	122	5	1.E-01	
A06	BoSF1509	56.3	0	50	0	0	129	0	4.E-01	
A06	BrSF0154	57.5	42	56	79	0	0	2	1.E-01	
A06	BrSF0153	59.3	44	50	85	0	0	0	7.E-01	
A06	BoSF1414	60.6	47	0	0	132	0	0	7.E-01	

A06	BoGMS0314	63.7	45	48	67	0	0	19	1.E-01	
A06	BoSF1522	65.3	49	0	0	130	0	0	5.E-01	
A06	IGF5707b	65.9	0	25	0	0	65	89	5.E-01	
A06	sS1949	66.1	47	0	0	126	0	6	5.E-01	
A06	BoSF0213	66.7	0	52	0	0	127	0	2.E-01	
A06	BrSF47-382a	76.7	38	56	83	0	0	2	1.E-01	
A06	BrSF47-389	78.9	40	58	75	0	0	6	3.E-02	
A06	BrSF0162	79.7	44	53	71	0	0	11	8.E-02	
A06	CNU400	80.0	44	57	78	0	0	0	9.E-02	
A06	BrSF47-277	81.9	36	56	71	0	0	16	2.E-02	
A06	BrSF47-234	82.8	33	57	72	0	0	17	1.E-02	
A06	BoSF1088	83.5	38	0	0	141	0	0	2.E-01	
A06	BoSF2406	83.8	42	60	72	0	0	5	1.E-02	
A06	BrSF0820	84.3	0	60	0	0	115	4	5.E-03	
A06	BrSF46-22	84.3	0	61	0	0	109	9	1.E-03	
A06	BrSF47-10	84.3	37	58	71	0	0	13	1.E-02	
A06	BrSF47-54	84.5	39	61	75	0	0	4	1.E-02	
A06	BrSF0821	84.7	39	0	0	140	0	0	3.E-01	
A06	BoSF0783a	84.7	39	0	0	136	0	4	4.E-01	
A06	BrSF0819	85.0	39	61	78	0	0	1	2.E-02	
A06	BrSF46-186	85.3	34	60	68	0	0	17	2.E-03	
A06	BrSF46-177	85.4	37	57	69	0	0	16	1.E-02	
A06	BrSF46-176a	85.7	35	53	64	0	0	27	2.E-02	
A06	BrSF46-167	86.4	38	60	70	0	0	11	5.E-03	
A06	BrSF46-306	87.4	36	58	73	0	0	12	1.E-02	
A06	BrSF46-291	87.8	33	53	64	0	0	29	1.E-02	
A06	BrSF46-238	88.2	39	60	69	0	0	11	5.E-03	
A06	BrSF0822	88.9	0	60	0	0	119	0	8.E-03	
A06	BrSF0166	93.8	0	60	0	0	119	0	8.E-03	
A06	BnID108	97.0	38	55	78	0	0	8	1.E-01	
A06	BrSF0144	97.7	41	56	82	0	0	0	2.E-01	
A06	BoSF2702	98.3	41	56	82	0	0	0	2.E-01	
A06	BnID107	99.1	37	57	80	0	0	5	6.E-02	
A06	BoSF2788	99.7	98	0	0	79	0	2	1.E-20	Zhongshuang11
A06	BoSF0215	113.3	42	48	85	0	0	4	8.E-01	
A07	sR4047	0.0	34	44	80	0	0	21	5.E-01	
A07	sR282	0.9	33	0	0	140	0	6	7.E-02	
A07	BoSF2344	3.7	39	53	87	0	0	0	3.E-01	
A07	BoSF0665	6.9	40	0	0	139	0	0	4.E-01	
A07	BRAS023	13.2	40	53	85	0	0	1	3.E-01	
A07	AT1G27050	16.3	39	52	87	0	0	1	4.E-01	
A07	BoSF2653	17.8	42	0	0	136	0	1	7.E-01	
A07	BrSF0331	20.1	42	51	85	0	0	1	5.E-01	
A07	CNU273	35.2	37	54	88	0	0	0	2.E-01	
A07	BrSF0185	35.2	22	25	42	0	0	90	8.E-01	
A07	sR7223	37.8	40	48	86	0	0	5	7.E-01	

A07	niab043	46.9	37	54	85	0	0	3	2.E-01	
A07	BRMS036	53.7	25	50	90	0	0	14	1.E-02	
A07	BoSF0898	60.5	0	49	0	0	130	0	5.E-01	
A07	CNU339	61.2	33	49	94	0	0	3	2.E-01	
A07	CNU037	62.7	35	47	96	0	0	1	3.E-01	
A07	BoGMS2110	69.6	0	57	0	0	121	1	3.E-02	
A07	CNU331b	71.1	41	0	0	135	0	3	6.E-01	
A07	CNU043	73.8	40	52	79	0	0	8	3.E-01	
A07	CNU053	80.4	0	47	0	0	130	2	6.E-01	
A07	CNU052	81.1	38	51	87	0	0	3	4.E-01	
A07	BoSF2257	82.1	36	0	0	138	0	5	2.E-01	
A07	BrSF0480	84.9	39	56	78	0	0	6	8.E-02	
A07	BoSF2703	87.9	41	53	82	0	0	3	3.E-01	
A07	BoSF0707	91.5	0	50	0	0	126	3	3.E-01	
A07	BoSF1972	92.5	0	48	0	0	127	4	5.E-01	
A07	CNU308	96.8	0	48	0	0	126	5	4.E-01	
A08	BoSF0711a	0.0	0	45	0	0	125	9	7.E-01	
A08	BnEMS1142	1.2	34	46	95	0	0	4	2.E-01	
A08	BrGMS0339a	4.3	26	46	92	0	0	15	3.E-02	
A08	BrSF0210	6.8	35	45	98	0	0	1	2.E-01	
A08	snap0675	13.4	0	57	0	0	122	0	3.E-02	
A08	BoSF2672	14.1	38	0	0	135	0	6	4.E-01	
A08	BnEMS0412	16.1	0	51	0	0	117	11	1.E-01	
A08	BrSF0606	19.0	39	54	82	0	0	4	2.E-01	
A08	BoSF1830	19.5	37	0	0	139	0	3	2.E-01	
A08	BoSF0693	23.3	42	54	82	0	0	1	3.E-01	
A08	BnEMS0236	23.3	21	27	38	0	0	93	4.E-01	
A08	sN12352	25.4	0	52	0	0	116	11	7.E-02	
A08	BrGMS0825	27.4	37	50	83	0	0	9	4.E-01	
A08	BoSF2499	32.6	41	53	79	0	0	6	2.E-01	
A09	KS51170	0.0	157	7	15	0	0	0	3.E-82	Zhongshuang11
A09	BrSF0916a	1.6	151	7	19	0	0	2	3.E-75	Zhongshuang11
A09	BnID044	3.7	0	38	0	0	139	2	3.E-01	
A09	BrSF0255	7.0	0	43	0	0	136	0	8.E-01	
A09	BnID043	8.3	40	31	97	0	0	11	8.E-02	
A09	BnID045	9.1	38	36	70	0	0	35	9.E-01	
A09	BnID047	9.5	43	38	86	0	0	12	8.E-01	
A09	BnID046	10.3	44	32	90	0	0	13	2.E-01	
A09	CNU157	11.4	46	39	94	0	0	0	6.E-01	
A09	BnID042	12.2	42	33	89	0	0	15	3.E-01	
A09	CNU004	12.9	40	0	0	128	0	11	7.E-01	
A09	BnID040	14.0	41	33	92	0	0	13	3.E-01	
A09	BnID041	14.5	0	29	0	0	134	16	3.E-02	
A09	BoSF0270	15.4	42	38	98	0	0	1	4.E-01	
A09	BoSF2624	34.3	55	40	83	0	0	1	2.E-01	
A09	BnID052	36.8	55	38	74	0	0	12	6.E-02	

A09	BnID053	37.1	59	36	76	0	0	8	2.E-02	
A09	ns156	40.9	0	49	0	0	130	0	5.E-01	
A09	BoSF2489	44.0	57	54	67	0	0	1	4.E-03	
A09	BrSF0998	45.8	56	44	76	0	0	3	9.E-02	
A09	CNU402	47.2	55	51	73	0	0	0	4.E-02	
A09	BnID056	49.0	29	24	39	0	0	87	3.E-01	
A09	ns155	49.5	0	54	0	0	125	0	1.E-01	
A09	BrSF6-104	50.1	57	49	65	0	0	8	5.E-03	
A09	ns162	53.1	0	56	0	0	122	1	5.E-02	
A09	ns160	55.1	0	55	0	0	119	5	4.E-02	
A09	ns158	56.4	62	0	0	117	0	0	3.E-03	
A09	ns161	56.8	0	52	0	0	121	6	1.E-01	
A09	BoSF0232	58.9	0	45	0	0	134	0	1.E+00	
A09	ns164	60.5	0	38	0	0	127	14	6.E-01	
A09	ns163	60.5	0	50	0	0	127	2	3.E-01	
A09	BnSF2342-39	68.9	56	45	72	0	0	6	4.E-02	
A09	ns165	70.7	55	0	0	109	0	15	1.E-02	
A09	ns166	70.7	0	56	0	0	120	3	4.E-02	
A09	ns168	70.7	0	50	0	0	129	0	4.E-01	
A09	ns167	70.7	0	34	0	0	127	18	3.E-01	
A09	BnID076	71.4	54	37	82	0	0	6	1.E-01	
A09	BnID075	72.1	51	40	83	0	0	5	4.E-01	
A09	BrSF6-2245	74.2	46	36	58	0	0	39	6.E-02	
A09	BrSF6-2562	**76.4**	57	36	73	0	0	13	2.E-02	
A09	BnSF400-253	76.8	0	37	0	0	129	13	4.E-01	
A09	BnID060	77.1	58	38	73	0	0	10	2.E-02	
A09	BrSF0385	78.0	51	34	81	0	0	13	2.E-01	
A09	BnID081	79.1	57	36	79	0	0	7	4.E-02	
A09	BoSF0333	79.6	56	0	0	119	0	4	3.E-02	
A09	BoSF2710	81.5	55	0	0	123	0	1	7.E-02	
A09	BoSF2709	81.8	55	38	86	0	0	0	2.E-01	
A09	ENA27	82.0	55	0	0	123	0	1	7.E-02	
A09	BnID082	82.9	0	49	0	0	118	12	2.E-01	
A09	BnSF400-28	95.9	34	0	0	134	0	11	2.E-01	
A09	BrSF6-2380	96.8	35	0	0	139	0	5	1.E-01	
A09	BrSF0383	100.2	37	85	45	0	0	12	2.E-14	No.73290
A09	CNU264	100.2	36	91	46	0	0	6	1.E-16	No.73290
A09	BrSF0251	102.0	38	100	40	0	0	1	8.E-22	No.73290
A09	BrSF6-2389	102.0	38	95	43	0	0	3	1.E-18	No.73290
A09	BnSF400-29	102.5	36	94	35	0	0	14	2.E-21	No.73290
A09	BrSF0353	103.2	36	97	44	0	0	2	1.E-19	No.73290
A09	BnSF400-54	103.6	0	89	0	0	72	18	7.E-19	No.73290
A09	BrSF0389a	103.8	0	88	0	0	79	12	1.E-16	No.73290
A09	BoSF0223	104.8	0	91	0	0	88	0	1.E-15	No.73290
A09	BoSF1814	105.3	0	96	0	0	81	2	3.E-19	No.73290
A09	BrSF0250	105.3	39	92	48	0	0	0	7.E-16	No.73290

A09	BrSF6-2413	105.3	0	84	0	0	85	10	1.E-13	No.73290
A09	BnID079	107.2	44	86	44	0	0	5	2.E-14	No.73290
A09	BnID078	108.2	37	99	36	0	0	7	5.E-23	No.73290
A09	BnID086	110.3	43	81	48	0	0	7	1.E-11	No.73290
A09	BnID087	111.1	43	87	45	0	0	4	2.E-14	No.73290
A09	BnID061	112.4	44	76	47	0	0	12	3.E-10	No.73290
A09	BnID090	112.5	28	0	0	144	0	7	8.E-03	
A09	BoSF1920a	112.6	0	87	0	0	90	2	1.E-13	No.73290
A09	BnID063	113.4	41	83	39	0	0	16	5.E-15	No.73290
A09	BnID094	114.4	43	80	48	0	0	8	2.E-11	No.73290
A09	BnID062	114.6	41	82	47	0	0	9	2.E-12	No.73290
A09	BnID085	116.4	50	78	47	0	0	4	8.E-11	No.73290
A09	BnID095	117.0	51	39	84	0	0	5	4.E-01	
A09	BrSF0615b	117.0	0	129	0	0	48	2	5.E-49	No.73290
A09	BoSF0872b	117.0	0	114	0	0	47	18	4.E-41	No.73290
A09	BrSF0229	118.0	0	66	0	0	112	1	2.E-04	No.73290
A09	BoSF1885	120.7	27	0	0	142	0	10	7.E-03	
A09	BoSF2104a	120.9	0	102	0	0	75	2	1.E-23	No.73290
A09	BnEMS0337	121.7	45	36	94	0	0	4	4.E-01	
A09	BoSF2791	122.4	0	131	0	0	46	2	3.E-51	No.73290
A09	BrSF77-118	123.3	0	26	0	0	55	98	1.E-01	
A09	BnID097	124.7	51	39	85	0	0	4	4.E-01	
A09	BrSF82-562	125.1	20	0	0	67	0	92	7.E-01	
A09	BoSF1902	125.8	34	50	92	0	0	3	2.E-01	
A09	BrSF0261	125.9	0	144	0	0	33	2	4.E-67	No.73290
A10	BrSF0898a	0.0	44	0	0	134	0	1	9.E-01	
A10	BrSF0958	4.9	47	50	82	0	0	0	5.E-01	
A10	Na10E08	10.6	38	42	62	0	0	37	3.E-01	
A10	BrSF0436b	10.6	0	19	0	0	70	90	4.E-01	
A10	sS2066	25.4	49	53	76	0	0	1	1.E-01	
A10	BoSF0652	26.2	49	0	0	127	0	3	4.E-01	
A10	BrSF0287	26.8	0	54	0	0	125	0	1.E-01	
A10	niab140	26.8	0	52	0	0	124	3	2.E-01	
A10	sR12384	30.3	0	52	0	0	126	1	2.E-01	
A10	BrSF0425a	30.5	41	39	50	0	0	49	3.E-02	
A10	BrSF0283	33.6	51	0	0	127	0	1	3.E-01	
A10	niab122	36.0	51	55	73	0	0	0	4.E-02	
A10	BoSF0601	36.7	0	54	0	0	122	3	8.E-02	
A10	BrSF0415	37.3	0	56	0	0	123	0	5.E-02	
A10	niab103	44.1	48	47	83	0	0	1	7.E-01	
A10	BrGMS0536	44.9	40	42	74	0	0	23	8.E-01	
A10	sN8502	47.2	23	23	42	0	0	91	9.E-01	
A10	BoSF1808	48.5	46	48	75	0	0	10	3.E-01	
C01	BrSF0279	0.0	0	46	0	0	133	0	8.E-01	
C01	BoSF2670	4.6	43	40	95	0	0	1	6.E-01	
C01	BoSF0224a	7.3	44	46	88	0	0	1	1.E+00	

C01	CB10587	11.1	43	44	84	0	0	8	1.E+00	
C01	CNU139c	15.1	44	0	0	133	0	2	1.E+00	
C01	BoSF1469	18.2	45	57	77	0	0	0	8.E-02	
C01	BnEMS1012	25.9	0	46	0	0	127	6	6.E-01	
C01	niab068	29.2	0	42	0	0	136	1	7.E-01	
C01	IGF2026c	31.8	41	49	89	0	0	0	7.E-01	
C01	BoSF0678a	32.0	40	49	90	0	0	0	6.E-01	
C01	CB10369b	34.1	39	0	0	137	0	3	4.E-01	
C01	BoSF0442	34.4	42	45	89	0	0	3	9.E-01	
C01	BoSF1667	35.5	42	47	89	0	0	1	9.E-01	
C01	BoSF1998	35.7	39	0	0	138	0	2	4.E-01	
C01	BoGMS1565a	41.4	37	42	94	0	0	6	5.E-01	
C01	BoSF2182	41.4	35	47	94	0	0	3	3.E-01	
C01	BoSF0130	48.1	31	44	102	0	0	2	5.E-02	
C01	BoSF0133	50.8	35	42	99	0	0	3	2.E-01	
C01	BoSF0114	59.7	0	25	0	0	154	0	7.E-04	Zhongshuang11
C01	BrSF0811	65.1	0	45	0	0	133	1	9.E-01	
C01	BoSF2314	68.8	40	44	88	0	0	7	9.E-01	
C01	BrSF0705	70.8	42	0	0	133	0	4	8.E-01	
C01	BoSF2346	70.9	0	43	0	0	133	3	9.E-01	
C01	BoSF2538	73.3	39	0	0	128	0	12	6.E-01	
C01	BoSF0050b	75.8	0	40	0	0	117	22	9.E-01	
C01	BoSF1372	76.8	41	44	93	0	0	1	8.E-01	
C01	BoSF2575	78.7	43	45	90	0	0	1	1.E+00	
C01	BoSF1270	85.0	35	39	105	0	0	0	6.E-02	
C01	ns258	91.3	58	0	0	121	0	0	2.E-02	
C01	ns059	94.1	0	52	0	0	127	0	2.E-01	
C01	ns262	94.5	48	0	0	131	0	0	6.E-01	
C01	BoSF1597	99.1	30	0	0	148	0	1	1.E-02	
C01	FITO283	99.5	30	0	0	147	0	2	1.E-02	
C01	BoSF2820	102.4	31	0	0	146	0	2	2.E-02	
C02	BoSF2071	0.0	0	58	0	0	120	1	2.E-02	
C02	BoSF1799	1.9	0	53	0	0	126	0	2.E-01	
C02	BoSF1425	2.4	0	53	0	0	125	1	1.E-01	
C02	BnGMS0081	2.8	0	46	0	0	121	12	4.E-01	
C02	BoSF1539	3.2	0	53	0	0	126	0	2.E-01	
C02	BoGMS0665	3.8	30	43	75	0	0	31	3.E-01	
C02	BoSF1017	4.5	0	52	0	0	127	0	2.E-01	
C02	BoSF0132	5.1	34	53	89	0	0	3	1.E-01	
C02	BoSF2604	5.5	35	53	83	0	0	8	1.E-01	
C02	BoSF1825	5.8	0	48	0	0	113	18	2.E-01	
C02	Ol11H09	5.9	40	54	82	0	0	3	2.E-01	
C02	BoSF1826	5.9	0	55	0	0	117	7	3.E-02	
C02	CNU461	19.4	39	43	96	0	0	1	5.E-01	
C02	BoSF1264	28.3	0	40	0	0	138	1	4.E-01	
C02	BrSF0065b	30.1	44	42	91	0	0	2	9.E-01	

C02	niab124	31.2	0	40	0	0	139	0	4.E-01	
C02	BoSF2242	34.3	0	40	0	0	139	0	4.E-01	
C02	snap0674	36.2	0	40	0	0	138	1	4.E-01	
C02	snap0673	39.1	0	48	0	0	131	0	6.E-01	
C02	BoSF0033a	41.4	44	30	98	0	0	7	6.E-02	
C02	BoSF2475	52.1	0	42	0	0	137	0	6.E-01	
C02	BoSF0880	57.4	45	0	0	134	0	0	1.E+00	
C02	BnEMS0959	57.9	43	35	98	0	0	3	2.E-01	
C02	CB10026	61.5	48	37	88	0	0	6	5.E-01	
C02	BoSF0096	64.6	51	37	88	0	0	3	3.E-01	
C02	BoSF0757	66.2	52	34	91	0	0	2	1.E-01	
C02	BoSF0525b	66.6	54	0	0	125	0	0	1.E-01	
C02	BoSF1743	67.0	53	38	86	0	0	2	3.E-01	
C02	BoSF0284	74.9	48	34	95	0	0	2	2.E-01	
C02	BoSF1995a	84.4	22	21	45	0	0	91	1.E+00	
C02	ns314	90.6	0	56	0	0	123	0	5.E-02	
C02	ns085	96.9	0	38	0	0	141	0	2.E-01	
C02	MD038	100.8	65	0	0	114	0	0	5.E-04	Zhongshuang11
C02	BrSF0503	107.6	61	38	79	0	0	1	2.E-02	
C02	BoSF0310	110.0	57	0	0	120	0	2	3.E-02	
C02	BoGMS2128	111.2	46	28	73	0	0	32	1.E-01	
C02	BoSF1258	112.2	58	37	84	0	0	0	6.E-02	
C02	BoSF2269	113.7	56	32	82	0	0	9	3.E-02	
C03	BoSF2181	0.0	52	47	77	0	0	3	2.E-01	
C03	sR12137	4.5	54	42	83	0	0	0	3.E-01	
C03	BoSF1005	6.5	0	43	0	0	134	2	8.E-01	
C03	BoSF1977	7.5	0	48	0	0	130	1	5.E-01	
C03	BoGMS0707	8.5	50	38	73	0	0	18	2.E-01	
C03	BRMS006	8.9	0	41	0	0	131	7	7.E-01	
C03	BoSF1944	9.5	0	42	0	0	133	4	8.E-01	
C03	BoSF0421	9.7	0	42	0	0	131	6	8.E-01	
C03	niab077	9.9	0	46	0	0	132	1	8.E-01	
C03	BoSF2263	10.4	49	44	79	0	0	7	5.E-01	
C03	niab115b	10.8	48	0	0	130	0	1	5.E-01	
C03	BoSF2861	12.4	52	0	0	126	0	1	2.E-01	
C03	BoSF1510	13.0	52	0	0	127	0	0	2.E-01	
C03	BoGMS0407	17.3	47	45	81	0	0	6	7.E-01	
C03	BoSF0371	18.8	0	46	0	0	131	2	8.E-01	
C03	BoSF1970	25.5	58	41	76	0	0	4	4.E-02	
C03	BoSF2392b	30.5	0	53	0	0	123	3	1.E-01	
C03	BoSF2006	32.0	46	49	79	0	0	5	5.E-01	
C03	BoSF2005	32.2	0	48	0	0	128	3	5.E-01	
C03	BoGMS0678	34.8	49	50	77	0	0	3	3.E-01	
C03	BnID035a	35.8	47	0	0	127	0	5	5.E-01	
C03	FITO218	38.8	0	45	0	0	133	1	9.E-01	
C03	BoSF0466	39.3	45	45	86	0	0	3	1.E+00	

C03	BrBAC087b	42.2	49	47	82	0	0	1	6.E-01	
C03	BoSF2132	42.8	47	45	86	0	0	1	9.E-01	
C03	BrSF0845b	43.3	49	0	0	128	0	2	4.E-01	
C03	BN12A	43.3	0	45	0	0	130	4	8.E-01	
C03	CB10569	43.7	46	0	0	131	0	2	8.E-01	
C03	BoSF0805	44.7	44	45	87	0	0	3	1.E+00	
C03	CB10235	48.4	42	41	83	0	0	13	1.E+00	
C03	BoSF2258	49.5	44	0	0	131	0	4	1.E+00	
C03	BoSF2284	51.2	40	47	90	0	0	2	7.E-01	
C03	BrSF0299	54.7	47	0	0	121	0	11	4.E-01	
C03	ns027b	56.1	41	47	85	0	0	6	8.E-01	
C03	Na12E02b	56.6	0	51	0	0	126	2	2.E-01	
C03	BoSF0670	58.1	39	48	86	0	0	6	6.E-01	
C03	Ol10E05	59.7	37	50	80	0	0	12	3.E-01	
C03	BoSF1271b	61.4	38	57	84	0	0	0	9.E-02	
C03	BoSF1276	63.8	36	58	82	0	0	3	4.E-02	
C03	BoSF0840	66.1	36	56	78	0	0	9	5.E-02	
C03	snap0622	67.0	40	0	0	139	0	0	4.E-01	
C03	BoSF0160	73.2	36	49	93	0	0	1	3.E-01	
C03	BoSF0402	74.1	0	50	0	0	128	1	3.E-01	
C03	BoSF1911	74.8	34	0	0	145	0	0	6.E-02	
C03	BoSF2669	75.6	34	45	92	0	0	8	3.E-01	
C03	BoSF0883	76.8	36	50	92	0	0	1	3.E-01	
C03	Na12G05	78.0	0	61	0	0	115	3	3.E-03	
C03	BrGMS2766	79.3	35	40	77	0	0	27	8.E-01	
C03	CB10057	81.3	34	39	90	0	0	16	4.E-01	
C03	BoGMS0369	82.6	0	43	0	0	119	17	7.E-01	
C03	snap0260	84.8	0	60	0	0	119	0	8.E-03	
C03	BoSF2281	92.1	0	46	0	0	129	4	7.E-01	
C03	Na10E02	93.4	0	47	0	0	127	5	5.E-01	
C03	BoSF2723a	97.1	47	41	82	0	0	9	7.E-01	
C03	BoSF0711b	104.8	41	40	90	0	0	8	8.E-01	
C03	BoSF2510	107.2	44	41	92	0	0	2	8.E-01	
C03	BoSF2509	108.7	42	0	0	135	0	2	7.E-01	
C03	BoSF2754	109.4	43	41	95	0	0	0	7.E-01	
C03	CALSSR	109.4	40	0	0	131	0	8	6.E-01	
C03	BoSF2233	109.4	43	40	95	0	0	1	6.E-01	
C03	BoSF2821	109.6	44	40	94	0	0	1	7.E-01	
C03	BoSF0176	110.5	0	40	0	0	137	2	5.E-01	
C03	BoGMS0819	112.1	41	40	86	0	0	12	9.E-01	
C03	BoSF0926a	113.7	45	0	0	133	0	1	9.E-01	
C03	BrSF0193	116.3	41	0	0	137	0	1	5.E-01	
C03	BRAS068	120.9	43	42	92	0	0	2	9.E-01	
C03	Ol13C12	121.8	38	43	89	0	0	9	7.E-01	
C03	MR049	123.3	43	43	92	0	0	1	9.E-01	
C03	BoSF0960	130.2	0	38	0	0	138	3	3.E-01	

C03	snap0833	134.0	0	40	0	0	135	4	5.E-01	
C03	BoSF2513	135.7	0	14	0	0	76	89	4.E-02	
C03	BrSF0869	138.8	0	34	0	0	138	7	1.E-01	
C03	BoSF1650	140.4	0	39	0	0	140	0	3.E-01	
C03	BoSF1136	144.4	0	42	0	0	137	0	6.E-01	
C03	BrSF0214	148.7	37	0	0	142	0	0	2.E-01	
C04	snap1060	0.0	59	0	0	120	0	0	1.E-02	
C04	BoSF1319	6.0	46	47	85	0	0	1	8.E-01	
C04	BrSF0313	9.8	40	0	0	132	0	7	6.E-01	
C04	BoSF2273	15.3	45	48	84	0	0	2	8.E-01	
C04	BoSF0349	17.2	49	48	81	0	0	1	5.E-01	
C04	BoSF1492	17.6	47	48	80	0	0	4	5.E-01	
C04	CNU286	20.7	43	0	0	135	0	1	8.E-01	
C04	BoSF0643	20.7	42	0	0	133	0	4	8.E-01	
C04	FITO366	21.3	42	0	0	137	0	0	6.E-01	
C04	BoSF0016	21.9	41	45	91	0	0	2	9.E-01	
C04	BoGMS1219	23.4	41	39	91	0	0	8	7.E-01	
C04	BoSF1271a	24.6	38	48	90	0	0	3	5.E-01	
C04	BoSF0318	25.6	36	48	94	0	0	1	3.E-01	
C04	BnEMS0038	28.3	32	41	75	0	0	31	6.E-01	
C04	BoSF1722	29.6	40	0	0	138	0	1	4.E-01	
C04	niab002	30.3	38	0	0	141	0	0	2.E-01	
C04	snap0205	40.5	0	53	0	0	126	0	2.E-01	
C04	snap1299	42.5	50	0	0	127	0	2	3.E-01	
C04	snap1891	45.0	33	0	0	146	0	0	4.E-02	
C04	BoSF2018	45.8	38	56	82	0	0	3	1.E-01	
C04	MR119	47.5	38	53	85	0	0	3	3.E-01	
C04	BoSF2723b	47.9	0	52	0	0	123	4	1.E-01	
C04	BoSF2106	52.3	37	42	99	0	0	1	3.E-01	
C04	BoSF2884	53.6	38	0	0	139	0	2	3.E-01	
C04	BoSF0952a	53.8	41	0	0	138	0	0	5.E-01	
C04	BoSF1336	54.1	0	40	0	0	137	2	5.E-01	
C04	BoSF0997	54.8	37	44	91	0	0	7	6.E-01	
C04	CB10428	54.9	0	45	0	0	134	0	1.E+00	
C04	BoSF0552	55.4	39	0	0	138	0	2	4.E-01	
C04	BoSF2040	55.6	38	43	98	0	0	0	4.E-01	
C04	BoSF1852	55.8	0	40	0	0	139	0	4.E-01	
C04	BoSF2062	56.0	0	39	0	0	133	7	5.E-01	
C04	IFLP013	56.1	0	16	0	0	74	89	1.E-01	
C04	BoSF0718	56.2	39	42	94	0	0	4	6.E-01	
C04	BoSF0600	56.2	0	42	0	0	134	3	7.E-01	
C04	BoSF1843	56.2	0	42	0	0	135	2	7.E-01	
C04	BoSF0592	56.3	37	42	96	0	0	4	4.E-01	
C04	BrSF0552	56.7	0	36	0	0	136	7	2.E-01	
C04	BoSF0949	56.7	38	0	0	141	0	0	2.E-01	
C04	BoSF1121	56.9	39	40	97	0	0	3	4.E-01	

C04	BoSF0943	57.3	38	0	0	139	0	2	3.E-01	
C04	BoGMS2091	58.2	37	39	93	0	0	10	4.E-01	
C04	BRAS061	59.0	0	41	0	0	137	1	5.E-01	
C04	BoSF1692	62.2	36	42	99	0	0	2	2.E-01	
C04	BoSF2872	69.5	36	50	91	0	0	2	3.E-01	
C04	BoSF0495	69.8	0	49	0	0	128	2	4.E-01	
C04	BoSF0935	70.2	0	49	0	0	128	2	4.E-01	
C04	sN464	70.5	0	48	0	0	131	0	6.E-01	
C04	BoGMS1031	71.0	34	42	84	0	0	19	5.E-01	
C04	BoSF0613	71.7	0	53	0	0	125	1	1.E-01	
C04	ns104	73.8	0	52	0	0	125	2	2.E-01	
C04	BoGMS0282	76.8	35	44	80	0	0	20	6.E-01	
C04	snap0299	80.2	0	63	0	0	115	1	1.E-03	
C04	BoSF2508	81.9	31	53	92	0	0	3	5.E-02	
C04	BoSF2294	84.4	0	47	0	0	128	4	6.E-01	
C04	BoSF0955	84.7	37	0	0	142	0	0	2.E-01	
C04	BoSF2666	86.3	32	52	92	0	0	3	9.E-02	
C04	ns123	89.8	0	55	0	0	124	0	8.E-02	
C04	ns120	91.9	0	58	0	0	120	1	2.E-02	
C04	ns126	94.8	0	54	0	0	125	0	1.E-01	
C04	BnGMS0160b	105.1	0	47	0	0	113	19	2.E-01	
C04	snap1360	110.4	0	53	0	0	122	4	1.E-01	
C04	BoSF2838	110.6	32	48	94	0	0	5	1.E-01	
C04	Ol11H02	113.0	30	50	94	0	0	5	6.E-02	
C04	MR155	116.7	31	53	92	0	0	3	5.E-02	
C04	BoSF1009	118.9	36	51	90	0	0	2	3.E-01	
C04	BoSF1937	123.4	0	59	0	0	120	0	1.E-02	
C05	BnEMS1131	0.0	44	43	90	0	0	2	1.E+00	
C05	sORH13	5.4	46	43	87	0	0	3	9.E-01	
C05	BoSF2552	8.6	42	0	0	134	0	3	7.E-01	
C05	BoSF0017	10.3	45	40	89	0	0	5	8.E-01	
C05	BoSF2607	11.8	40	0	0	118	0	21	9.E-01	
C05	BoSF1212a	13.1	28	25	36	0	0	90	2.E-01	
C05	BoSF2676	24.2	46	40	89	0	0	4	8.E-01	
C05	BoGMS2688	26.7	42	32	85	0	0	20	4.E-01	
C05	BoSF1663	30.7	43	0	0	132	0	4	9.E-01	
C05	BoSF0022b	32.0	43	39	96	0	0	1	5.E-01	
C05	CB10374	39.3	46	0	0	128	0	5	7.E-01	
C05	BoSF0230	43.7	0	35	0	0	144	0	9.E-02	
C05	Na12C01	50.4	53	33	87	0	0	6	1.E-01	
C05	BrSF0858a	50.8	51	0	0	118	0	10	1.E-01	
C05	BoSF1969	52.0	54	33	91	0	0	1	8.E-02	
C05	BoSF2019	52.5	54	33	88	0	0	4	8.E-02	
C05	ns033	57.3	0	37	0	0	142	0	2.E-01	
C05	BoSF1918	58.3	51	0	0	128	0	0	3.E-01	
C05	BoSF0222	58.5	0	36	0	0	140	3	2.E-01	

C05	sS2129	59.2	52	37	87	0	0	3	3.E-01	
C05	Na10D11	59.7	49	33	82	0	0	15	2.E-01	
C05	sS2131	60.0	54	38	86	0	0	1	2.E-01	
C05	CB10170	61.0	54	37	87	0	0	1	2.E-01	
C05	BrSF0040	65.6	52	35	90	0	0	2	2.E-01	
C05	BoSF0926b	66.6	0	35	0	0	143	1	1.E-01	
C05	BRAS040	67.3	51	36	87	0	0	5	3.E-01	
C06	ns337	0.0	42	0	0	137	0	0	6.E-01	
C06	ns326	7.0	42	0	0	137	0	0	6.E-01	
C06	BoSF0455b	16.0	42	0	0	133	0	4	8.E-01	
C06	BoSF0733	16.0	0	45	0	0	132	2	9.E-01	
C06	BoSF1665	16.5	13	27	46	0	0	93	8.E-02	
C06	BoSF2316	17.7	44	0	0	135	0	0	9.E-01	
C06	snap0695b	30.3	47	0	0	124	0	8	5.E-01	
C06	BoSF0513	35.1	45	0	0	133	0	1	9.E-01	
C06	BoSF2395	35.6	44	0	0	135	0	0	9.E-01	
C06	BoSF1365	38.1	43	0	0	135	0	1	8.E-01	
C06	snap1395	41.8	46	0	0	128	0	5	7.E-01	
C06	Na12E01b	42.8	0	30	0	0	145	4	2.E-02	
C06	snap1392	45.6	42	0	0	136	0	1	7.E-01	
C06	BoSF0952b	48.7	56	0	0	114	0	9	2.E-02	
C06	MR133.1	50.2	0	31	0	0	143	5	3.E-02	
C06	BrSF0784	51.5	0	32	0	0	146	1	3.E-02	
C06	BoSF2507	52.6	45	32	94	0	0	8	2.E-01	
C06	BoSF1254	53.2	44	0	0	135	0	0	9.E-01	
C06	BoSF2506	54.5	44	39	91	0	0	5	7.E-01	
C06	BoSF0760	55.8	43	0	0	133	0	3	9.E-01	
C06	BoSF1126	60.0	0	36	0	0	142	1	1.E-01	
C06	BoSF2591	62.1	48	0	0	130	0	1	5.E-01	
C06	sR12387	76.0	37	0	0	134	0	8	3.E-01	
C06	BoSF1406	76.3	18	0	0	71	0	90	3.E-01	
C06	BoSF1144	76.9	39	46	94	0	0	0	6.E-01	
C06	BoSF1468	77.7	38	48	93	0	0	0	5.E-01	
C06	BoSF1951	82.6	0	46	0	0	132	1	8.E-01	
C06	BnEMS0350	85.1	0	49	0	0	124	6	3.E-01	
C06	CNU331a	93.7	40	0	0	128	0	11	7.E-01	
C06	Na10C06	99.6	48	0	0	131	0	0	6.E-01	
C06	ns347	109.7	0	56	0	0	123	0	5.E-02	
C07	CB10534	0.0	42	44	93	0	0	0	9.E-01	
C07	BoSF0778	8.2	40	37	101	0	0	1	2.E-01	
C07	BoSF2740	10.5	37	39	102	0	0	1	1.E-01	
C07	BoSF2771	11.9	36	37	103	0	0	3	8.E-02	
C07	BoSF1559	14.6	0	39	0	0	140	0	3.E-01	
C07	BoSF1324	18.4	0	44	0	0	135	0	9.E-01	
C07	BoSF0783b	20.3	22	17	50	0	0	90	4.E-01	
C07	BrSF46-176b	32.1	0	37	0	0	125	17	5.E-01	

C08	ns067	0.0	0	60	0	0	117	2	6.E-03	
C08	BoGMS1419	10.8	38	48	86	0	0	7	6.E-01	
C08	BoSF0521	13.6	35	40	101	0	0	3	1.E-01	
C08	BoSF0586a	16.9	34	51	93	0	0	1	2.E-01	
C08	BoGMS0456	17.5	34	44	81	0	0	20	5.E-01	
C08	BoSF2274	18.8	34	50	94	0	0	1	2.E-01	
C08	BoSF2646	19.4	33	49	93	0	0	4	2.E-01	
C08	CB10053	19.9	35	49	95	0	0	0	2.E-01	
C08	BoSF1481	20.4	0	46	0	0	130	3	7.E-01	
C08	BoSF0554	21.8	0	47	0	0	127	5	5.E-01	
C08	BoSF2163	26.7	0	51	0	0	128	0	3.E-01	
C08	BoSF2482	31.5	0	50	0	0	129	0	4.E-01	
C08	BnGMS0509	32.8	30	53	86	0	0	10	4.E-02	
C08	snap1382	36.2	0	62	0	0	117	0	3.E-03	
C08	BoSF2378	37.2	0	47	0	0	130	2	6.E-01	
C08	BoSF0023	38.5	33	48	97	0	0	1	1.E-01	
C08	BoSF2290	43.5	29	47	97	0	0	6	4.E-02	
C08	BnEMS0020	53.4	36	37	90	0	0	16	4.E-01	
C08	BoSF1926	56.8	32	44	102	0	0	1	7.E-02	
C08	BoSF2347	66.2	33	48	93	0	0	5	2.E-01	
C08	BoSF2751	67.9	34	42	96	0	0	7	2.E-01	
C08	BoSF2616	73.4	40	47	92	0	0	0	7.E-01	
C08	BoSF1222	74.8	41	46	92	0	0	0	8.E-01	
C08	BnSF287-186	80.6	34	55	80	0	0	10	6.E-02	
C08	niab129	81.5	37	51	86	0	0	5	3.E-01	
C08	BrSF0356	82.7	39	50	90	0	0	0	5.E-01	
C08	BrGMS0447	82.7	36	45	95	0	0	3	4.E-01	
C08	CB10179	85.3	41	50	84	0	0	4	5.E-01	
C08	IFLP021	92.1	32	52	95	0	0	0	8.E-02	
C08	BoSF0365	93.8	36	49	92	0	0	2	3.E-01	
C08	BnEMS1070	95.3	29	49	91	0	0	10	6.E-02	
C08	BoSF1064	98.2	31	47	98	0	0	3	7.E-02	
C08	BoSF0448	98.5	0	48	0	0	130	1	5.E-01	
C08	BoSF2073	101.0	33	44	101	0	0	1	1.E-01	
C08	BnEMS0606	103.8	29	45	92	0	0	13	8.E-02	
C08	sR5795	105.8	31	51	96	0	0	1	6.E-02	
C08	BoSF1920b	107.2	0	55	0	0	123	1	7.E-02	
C08	BN26A	107.2	33	0	0	144	0	2	5.E-02	
C08	BrSF0549a	108.0	0	51	0	0	127	1	3.E-01	
C08	CB10092	111.9	28	53	97	0	0	1	1.E-02	
C08	CB10028	112.2	25	52	99	0	0	3	4.E-03	
C08	BrSF53-712	115.6	0	52	0	0	121	6	1.E-01	
C09	BoSF0980	0.0	52	48	72	0	0	7	9.E-02	
C09	BoSF0336b	3.4	46	43	68	0	0	22	2.E-01	
C09	BrSF54-590	4.6	50	0	0	97	0	32	1.E-02	
C09	BoSF2578	5.8	0	48	0	0	131	0	6.E-01	

C09	BoSF0948	8.8	0	51	0	0	125	3	2.E-01	
C09	BnGMS0213	9.8	54	47	78	0	0	0	2.E-01	
C09	BoSF2398	11.2	56	45	69	0	0	9	2.E-02	
C09	BoSF1228	12.5	51	47	80	0	0	1	4.E-01	
C09	BoSF1976	12.9	53	49	77	0	0	0	2.E-01	
C09	BoSF2748	13.6	0	45	0	0	122	12	6.E-01	
C09	BoSF1872	14.0	51	49	77	0	0	2	2.E-01	
C09	BoSF1810	15.9	52	47	79	0	0	1	3.E-01	
C09	BrSF1008	16.8	49	0	0	130	0	0	5.E-01	
C09	BoGMS1561	17.1	0	48	0	0	119	12	3.E-01	
C09	BoSF1788	17.1	0	50	0	0	127	2	3.E-01	
C09	BoSF1817	18.1	41	49	81	0	0	8	5.E-01	
C09	BoGMS1453	20.5	40	44	75	0	0	20	7.E-01	
C09	BoSF0040	20.5	43	50	85	0	0	1	6.E-01	
C09	BoSF1647	22.4	0	45	0	0	131	3	9.E-01	
C09	BoSF2393	22.8	39	45	80	0	0	15	8.E-01	
C09	BoSF2774	23.6	46	0	0	127	0	6	6.E-01	
C09	BrSF0436a	24.1	45	49	85	0	0	0	7.E-01	
C09	Ol12A04	38.4	0	42	0	0	115	22	6.E-01	
C09	BoSF0185	39.7	48	48	71	0	0	12	2.E-01	
C09	BrSF0425b	41.2	42	45	82	0	0	10	9.E-01	
C09	BoSF0483	44.2	43	48	88	0	0	0	8.E-01	
C09	BrSF0369	45.7	41	0	0	137	0	1	5.E-01	
C09	BoGMS1473	49.7	47	0	0	119	0	13	3.E-01	

Table S4 Identification of genomic arrangements between the pseudochromosomes of *B. napus* and *B. rapa* /*B. oleracea* through comparative mapping.

Linkage_group	Marker_name	Genetic position (cM)	Physical position_*B.napus* (Mb)	Physical position_*B.rapa* /*B.oleracea* (Mb	Genomic rearrangement
A01	niab097a	6.137	0.638	1.041	
A01	BrGMS0081b	0.000	0.798	1.201	
A01	BoSF2770	8.826	0.885	1.282	
A01	BnEMS0886	13.258	1.026	1.428	
A01	niab106	27.155	1.062	1.466	
A01	niab071	37.502	1.324	1.787	
A01	BrSF0019	43.442	1.360	1.824	
A01	CNU142c	41.508	1.447	1.926	
A01	BoSF0224b	44.260	1.537	2.037	
A01	CNU139a	39.177	1.623	2.133	
A01	BoSF1331a	46.816	2.452	0.083	Translocation
A01	BoSF0302	44.813	2.567	0.183	Translocation
A01	CB10081	50.398	2.700	0.324	Translocation
A01	BRMS037	50.398	2.700	0.324	Translocation
A01	niab113a	51.996	2.743	0.367	Translocation
A01	BrSF0403	56.532		2.952	
A01	BoSF2131	59.369	2.828	3.125	
A01	CB10099a	69.142	3.903	4.226	
A01	BrSF0016	72.156	5.670	6.187	
A01	BrSF0446	82.652	8.537	9.917	
A01	CB10189	83.543	8.891	10.266	
A01	BoSF1659	91.434	9.995	11.205	
A01	BoSF0503	89.807	10.354	11.639	
A01	CNU132b	89.807	10.354	11.639	
A01	Ra2G05	90.667	10.428	11.565	
A01	BoSF0764	87.526	14.715	16.385	
A01	BrSF0702	93.336	14.863	16.498	

A01	BrSF0703	94.070	15.559	16.658	
A01	SA63	93.813	15.648	16.747	
A01	BrSF0706	94.994		17.373	
A01	BrSF0010	98.066	15.713	17.715	
A01	CNU068	98.066	15.713	17.715	
A01	BrSF0009	98.469	15.761	17.748	
A01	BoSF0050a	99.589	16.498	19.182	
A01	BrSF0006	102.109	17.654	20.337	
A01	CNU397	103.201	17.654	20.338	
A01	BrSF1002	104.651	16.378	21.166	
A01	TFZIP23	105.810	18.273	22.862	
A01	sN3523	106.942	18.773	21.647	
A01	BrSF0982	113.857		22.574	
A01	BoSF0911	119.126	21.557	26.906	
A01	BoSF2123	124.408			
A01	BrSF0973	125.451			
A01	Na14F11b	126.504		27.720	
A01	CNU388	123.510	22.720	27.924	
A01	CNU474	122.062	22.733	27.937	
A01	BrSF0949	128.788	22.744	27.946	
A01	BoSF0197	124.959	23.248	25.047	
A01	BoSF1939	124.130	23.811	25.609	
A02	niab025	0.000	0.124	1.257	
A02	sR94102	6.091	0.622	3.137	
A02	BoSF0239	12.750	1.390	3.994	
A02	BrGMS0511	11.109	1.567	4.171	
A02	BrSF0065a	11.495	1.564	4.188	
A02	BoSF2549	5.653	1.661	4.285	
A02	BrSF0051	9.366	1.687	4.311	
A02	MR052	20.344	1.958	4.583	

A02	BrSF0665	19.076	2.154	5.039	
A02	BRAS083	18.449	3.140	5.945	
A02	BrSF0062	23.627	3.258	6.062	
A02	BRAS037	22.352	3.512	6.335	
A02	BrSF0661	24.901	3.709	6.493	
A02	BoSF2224	27.243	3.773	6.555	
A02	sN3761	26.170	3.973	6.755	
A02	BrSF0663	27.955	3.976	6.757	
A02	sR6293	29.867	4.211	7.173	
A02	BrSF0045	30.517	4.294	7.256	
A02	BoSF0329	31.636	4.414	7.392	
A02	BrSF0049	38.209	4.815	7.834	
A02	BrSF0750	33.482	4.996	8.014	
A02	BrSF0053	33.958	5.187	8.205	
A02	BrSF0052	34.232	5.223	8.241	
A02	BrSF0754	34.611	5.576	8.626	
A02	BrSF0755	36.160	5.996	9.046	
A02	BrSF0031	43.056	7.043	10.197	
A02	BoSF1714	43.632	7.050	10.206	
A02	CNU046	41.949	7.105	10.253	
A02	BoSF2100	45.934	8.340	11.335	
A02	BrSF0056	47.796	8.621	11.912	
A02	BrSF0570	47.181	8.945	12.234	
A02	IFLP018	51.207	9.507		
A02	BoSF0216	53.595	10.518	12.319	
A02	FITO255	55.186	10.709	12.511	
A02	BoSF0926c	56.204	11.268	14.490	
A02	BrSF0680	58.123	11.097	14.653	
A02	BrSF0679	48.538	11.058	14.693	
A02	BrSF0682	52.142	12.574	16.347	

A02	BoSF2657	60.739	14.049	16.838	
A02	BrSF0032	61.277	14.270	17.090	
A02	BrSF1044	60.560	14.523	17.142	
A02	BrSF0695	61.096	14.661	17.233	
A02	BrSF0696	60.908	14.799	17.372	
A02	BrSF0836	61.383	16.482		
A02	BrSF0472	61.298	17.422	18.321	
A02	BoSF1924	58.519	17.659	18.558	
A02	BnEMS0401	65.481	17.732	18.632	
A02	CB10628	61.673	18.212	19.174	
A02	BrSF0520	61.821	18.310	19.267	
A02	BrSF0519	62.212	18.346	19.393	
A02	BoSF2324	63.208	18.402	19.444	
A02	BrSF0689	67.263	13.178	21.952	Translocation
A02	BoSF2214	79.579	22.380	24.248	
A02	Na14B05	82.883	22.654	24.522	
A03	niab115a	0.000	0.882	1.050	
A03	BrSF0427	15.601	1.430	1.597	
A03	BoSF1704	6.235	1.487	1.880	
A03	BrSF0029	18.587	2.218	2.665	
A03	BrSF0422	12.803	3.052	3.500	
A03	CB10036	16.469	3.618	4.065	
A03	BoSF2852	17.061	3.665	4.140	
A03	BrSF0350	16.072	3.703	4.165	
A03	BrGMS0642	12.057	3.824	4.295	
A03	BoSF2392a	20.613	4.090	4.569	
A03	TFZIP19	21.126	4.132	4.630	
A03	BrSF0090	21.803	4.458	5.026	
A03	KS50980	21.803	4.458	5.026	
A03	BrSF0091	25.358	4.529	5.095	

A03	BrSF0089	24.215	4.550	5.119	
A03	sR6688a	23.346	4.554	5.130	
A03	BoSF2807	26.810	5.379	5.966	
A03	BrSF0845a	25.748	5.414	6.000	
A03	BoSF0546	28.826	5.453	6.036	
A03	BrSF0087a	26.284	5.463	6.047	
A03	BoSF0560	30.901	5.881	6.465	
A03	IGF2134f2	33.461	6.342	7.056	
A03	BnEMS0894	32.160	6.496	7.221	
A03	BrSF0092	35.003	6.734	7.442	
A03	BoSF2277	40.535	7.084	7.791	
A03	BrSF0300	39.258	7.216	7.931	
A03	Na12E02a	38.657	7.237	7.954	
A03	Ol11B05	38.724	7.304	8.020	
A03	BoSF0415b	40.190	7.330	8.041	
A03	CNU253	36.982	7.367	8.074	
A03	CNU288	44.098	8.774	9.504	
A03	BoSF0664	45.234	8.994	9.763	
A03	BrSF0114	44.940			
A03	BoSF2744	48.097	9.708	10.529	
A03	BoSF2220	51.321	10.039	10.860	
A03	BoSF1709	50.759	10.175	11.057	
A03	BrSF0078	49.846	10.189	11.062	
A03	CNU466	48.903	10.435	11.321	
A03	BoSF2915	52.768	10.825	11.698	
A03	BoSF1715	55.365	11.303	12.186	
A03	CB10329	55.365	11.303	12.186	
A03	BrSF0295	55.903	11.411	12.336	
A03	BoSF1886	57.487	11.787	12.656	
A03	CNU098	62.056	13.536	14.358	

A03	BoSF1678	62.056	13.536	14.358	
A03	CNU259	63.987	13.786	14.616	
A03	FITO285	66.467	14.178	15.062	
A03	BoSF1651	66.975	14.299	15.242	
A03	BrSF0102	68.405	14.556	15.495	
A03	Na14G10	70.646	14.776	15.716	
A03	Ol11G11	70.646	14.776	15.716	
A03	CNU422	69.766	14.828	15.756	
A03	sN11722	73.614	15.085	16.001	
A03	CB10114	73.614	15.085	16.001	
A03	BoSF0598	73.745	15.125	16.042	
A03	BoSF2828	76.066	15.430	16.303	
A03	BoSF1085	98.859	20.728	21.933	
A03	CNU371	100.367	20.765	21.965	
A03	CNU223	101.388	20.873	22.058	
A03	sR11644	105.768	21.157	22.380	
A03	BoSF2145	104.130	21.184	22.406	
A03	BoSF0346	105.853	21.190	22.412	
A03	BoSF0393	105.853	21.191	22.412	
A03	MR181	109.775			
A03	KS40432	111.819	22.713	24.118	
A03	BoGMS1071	110.630	24.483	24.036	
A03	BrSF0708	118.254	23.229	24.850	
A03	BnGMS0616	114.400	23.462	25.083	
A03	BoSF1919	115.995	23.622	25.243	
A03	BoSF1779	121.972	23.997	25.618	
A03	BrSF0918	129.106	24.797	26.405	
A03	CB10425	126.413	25.015	26.638	
A04	BrSF0564	0.000	0.895	1.152	
A04	BrSF0560	5.422	1.585	1.857	

A04	BrSF0112	6.644	2.321	2.616	
A04	BoSF2609	1.806	2.855	3.150	
A04	BoSF0336a	11.158	7.258	5.472	
A04	IFLP014	13.370	7.593	5.807	
A04	BnGMS0115	14.865	8.598	6.724	
A04	BrSF1049	17.256	9.183	7.055	
A04	BrSF1032	18.017	9.530	7.403	
A04	niab143	19.104	10.508	8.811	
A04	BoSF1125	20.424	10.608	8.911	
A04	sN2025	24.625	11.525	9.809	
A04	CNU246	33.342	13.166	11.604	
A04	BoSF1161	35.118	13.312	11.753	
A04	CNU039	36.054	13.913	12.572	
A04	BrSF0726	47.004	15.297	14.271	
A04	BoSF0415a	48.352	15.580	14.583	
A04	BoSF0482	50.058	15.620		
A04	BrSF0719	50.566	15.781	14.745	
A04	BoSF1476	52.094			
A04	BoSF1555	60.071	16.202	15.433	
A04	BrSF0645	57.732	16.326	15.557	
A04	BrSF0646	54.765	16.467	15.698	
A04	BoSF0231	56.838	16.505	15.749	
A04	BrSF0120	56.303	16.614	15.858	
A04	IFLP036	59.688	17.067	16.311	
A04	BrSF0642a	64.418	17.721	17.036	
A04	BrSF0643	63.206	17.895	17.216	
A04	BoSF1642	62.457	18.015	17.341	
A04	BoSF0841b	68.278	18.259	17.601	
A04	BoSF2769	70.580	18.524	17.862	
A04	BrSF0969	79.398	19.073		

A04	BrSF0972	87.075	18.963	18.342	
A05	BoSF1666	0.000	1.932	1.810	
A05	Na14G02	3.685			
A05	BrSF0712	4.773			
A05	BoSF2141	14.432	7.955	9.762	
A05	BrSF0975a	20.703	8.626	10.433	
A05	ns231	12.303	9.363	10.623	
A05	ns218	12.089	9.824	11.084	
A05	ns213	11.287	10.128	11.595	
A05	Na12E01a	22.757			
A05	BrSF0952	24.498	10.921	14.302	reshuffling
A05	BoSF1098	21.205	11.957	11.998	reshuffling
A05	BrSF0942	20.763	11.962	15.492	reshuffling
A05	CNU398	20.763	11.962	15.492	reshuffling
A05	BrSF0550	25.121	12.489	12.492	reshuffling
A05	CNU029	24.725	12.504	12.508	reshuffling
A05	BnGMS0293	24.487	13.874	14.130	reshuffling
A05	BrSF1031b	26.496		15.654	
A05	BrSF0849	26.550		16.452	
A05	BrSF0620	23.775	15.937	17.746	
A05	BrSF0618	29.904	16.298	18.002	
A05	BrGMS0293	34.224	17.371	19.141	
A05	ns211	28.085	18.227	20.013	
A05	BoSF1230	37.693	18.323	20.109	
A05	IFLP039	42.296	18.623	20.410	
A05	BoSF1749	46.577	18.743	20.543	
A05	BoSF0582	45.996	18.827	20.620	
A05	BrSF0497	45.653	18.872	20.664	
A05	BoSF0259	46.101	18.996	20.788	
A05	BrSF0491	52.510	19.946	21.770	

A05	CNU268	49.231	20.118	21.979	
A05	snap1047a	55.602	20.194	22.070	
A05	BoSF2746	50.565	20.219	22.101	
A05	BoSF2030	51.322	20.281	22.163	
A06	BoSF0784	0.000	0.936	0.896	
A06	BrSF0149	1.122	1.109	1.069	
A06	BoSF2253	2.036			
A06	BoSF0869	4.756	1.839	1.873	
A06	BoSF0455c	5.725	1.849	1.895	
A06	CB10143	9.301	2.251	2.242	
A06	BrSF0860	7.427	2.259	2.255	
A06	BoSF1212b	13.533	2.549	2.588	
A06	IFLP001	12.059	2.617	2.670	
A06	BrSF0741	17.742	3.194	3.377	
A06	BoSF1521	19.652	3.266	3.448	
A06	BrSF0743	21.424	3.422	3.653	
A06	BoSF2583	25.721	4.053	4.263	
A06	BoSF0022a	30.223	4.374	4.555	
A06	BRAS052	34.204	4.394	4.583	
A06	BoSF2360	29.500	4.432	4.621	
A06	BrSF0171	39.307	5.818	6.379	
A06	BoSF1990	41.412	5.916	6.477	
A06	BoSF1752	42.769	6.138	6.751	
A06	BnGMS0480	46.507	7.247	7.606	
A06	IGF1653c	47.255	7.300	7.664	
A06	CNU050	47.136	7.418	7.784	
A06	CB10006	48.109	8.010	8.466	
A06	CNU149	47.603	8.051	8.532	
A06	BrSF0858b	48.764	8.538	9.070	
A06	BrSF0789	52.464	16.332	14.852	

A06	BoSF1414	60.554	17.936	16.424	
A06	BrSF0154	57.530	18.451	17.034	
A06	BoSF2407	55.461	18.470	17.054	
A06	BoSF1509	56.327	18.771	17.378	
A06	ENA23	54.901	18.941	17.545	
A06	BrSF0153	59.325	19.147	17.751	
A06	sS1949	66.073	19.329	17.910	
A06	BoSF1522	65.324	19.391	17.972	
A06	BoGMS0314	63.709	19.447	18.069	
A06	BoSF0213	66.688	19.838	18.443	
A06	IGF5707b	65.937	19.880	18.506	
A06	BrSF47-389	78.872	21.656	22.617	
A06	BrSF47-382a	76.704	21.664	22.625	
A06	CNU400	80.039	21.700	22.668	
A06	BrSF0162	79.727	21.700	22.669	
A06	BrSF47-277	81.929	21.870	22.845	
A06	BrSF47-234	82.832	21.965	22.915	
A06	BoSF1088	83.466	21.977	22.927	
A06	BrSF47-54	84.485	22.315	23.268	
A06	BoSF0783a	84.726	22.402	23.355	
A06	BrSF47-10	84.307	22.404	23.357	
A06	BoSF2406	83.843		23.371	
A06	BrSF0820	84.304	22.405	23.427	
A06	BrSF46-22	84.304	22.405	23.427	
A06	BrSF0821	84.681	22.424	23.448	
A06	BrSF0819	84.961	22.590	23.614	
A06	BrSF46-167	86.399	22.671	23.686	
A06	BrSF46-176a	85.709	22.683	23.699	
A06	BrSF46-177	85.365	22.686	23.699	
A06	BrSF46-186	85.269	22.695	23.707	

A06	BrSF46-238	88.186	22.787	23.806	
A06	BrSF0822	88.918	22.837	23.850	
A06	BrSF46-291	87.775	22.893	23.906	
A06	BrSF46-306	87.383	22.927	23.940	
A06	BrSF0166	93.842	23.131	24.232	
A06	BnID107	99.121	23.515	24.615	
A06	BnID108	96.956	23.566	24.667	
A06	BoSF2788	99.684	23.603	24.704	
A06	BoSF2702	98.257	23.628	24.725	
A06	BrSF0144	97.692	23.736	24.829	
A06	BoSF0215	113.265	25.150	26.244	
A07	sR4047	0.000	0.756	0.829	
A07	sR282	0.886	1.641	1.564	
A07	BoSF2344	3.692	5.038	3.080	
A07	BoSF0665	6.885	6.877	5.018	
A07	AT1G27050	16.259	9.070	7.722	
A07	BoSF2653	17.782	10.078	8.654	
A07	BRAS023	13.176	10.114	8.683	
A07	BrSF0331	20.103	10.195	8.750	
A07	CNU273	35.174	14.472	12.545	
A07	BrSF0185	35.174	14.472	12.545	
A07	sR7223	37.794	14.441	12.315	
A07	niab043	46.866	15.886	13.959	
A07	BRMS036	53.675	18.575	16.648	
A07	BoSF0898	60.479	20.077	18.185	
A07	CNU037	62.737	20.117	18.224	
A07	CNU339	61.221	20.118	18.226	
A07	BoGMS2110	69.554	20.736	18.917	
A07	CNU043	73.766	21.730	19.911	
A07	CNU331b	71.117	21.929	20.167	

A07	BoSF2257	82.081	22.564	21.015	
A07	CNU053	80.449	22.962	21.412	
A07	CNU052	81.096	23.017	21.479	
A07	BrSF0480	84.890	23.162	21.619	
A07	BoSF2703	87.874	23.163	21.620	
A07	BoSF0707	91.481	23.823	22.280	
A07	BoSF1972	92.450	23.871	22.460	
A07	CNU308	96.838	23.623	22.224	
A08	BrGMS0339a	4.326	13.905	16.432	
A08	BrSF0210	6.837	14.092	16.637	
A08	BnEMS1142	1.210	14.229	16.757	
A08	BoSF0711a	0.000	14.574	17.100	
A08	BoSF2672	14.103	14.848	17.391	
A08	snap0675	13.367	15.365	17.926	
A08	BnEMS0412	16.054	15.479	18.027	
A08	BrSF0606	18.997	15.748	18.295	
A08	BoSF1830	19.520	15.771	18.319	
A08	BnEMS0236	23.297	16.105	18.653	
A08	BoSF0693	23.297	16.105	18.653	
A08	sN12352	25.407	16.241	18.889	
A08	BrGMS0825	27.440	16.267	18.915	
A08	BoSF2499	32.550	16.515	19.177	
A09	KS51170	0.000	3.888	4.115	
A09	BrSF0916a	1.585	4.806	4.899	
A09	BnID040	13.982	4.863	5.191	
A09	BoSF0270	15.427	4.933	5.258	
A09	BnID041	14.524	5.029	5.354	
A09	BnID042	12.161	5.072	5.523	
A09	CNU157	11.414	5.221	5.671	
A09	BnID043	8.277	5.257	5.698	

A09	BnID044	3.719	5.362	5.803	
A09	BnID045	9.123	5.369	5.810	
A09	CNU004	12.892	5.397	5.852	
A09	BnID046	10.253	5.454	5.905	
A09	BrSF0255	6.978	5.729	7.240	
A09	BnID047	9.543	5.739	7.250	
A09	BoSF2624	34.291	17.581	20.650	
A09	BnID052	36.781	19.764	22.347	
A09	BnID053	37.051	19.792	22.381	
A09	BrSF0998	45.764	22.810	24.788	
A09	CNU402	47.167	23.197	25.175	
A09	BnID056	48.993	23.268	25.245	
A09	BoSF2489	43.963	23.604	25.458	
A09	ns155	49.486	24.006	25.921	
A09	ns156	40.901	24.018	25.934	
A09	ns158	56.370	24.143	26.068	
A09	BrSF6-104	50.096	24.217	26.148	
A09	ns160	55.118	26.700	28.759	
A09	ns161	56.753	26.700	28.759	
A09	ns162	53.123	26.705	28.765	
A09	BoSF0232	58.919	26.729	28.797	
A09	ns163	60.472	26.748	28.817	
A09	ns164	60.472	26.748	28.817	
A09	BnSF2342-39	68.871	28.125	30.342	
A09	ns165	70.699	28.129	30.346	
A09	ns166	70.699	28.129	30.346	
A09	ns167	70.699	28.129	30.346	
A09	ns168	70.699	28.129	30.346	
A09	BnID075	72.104	28.244	30.449	
A09	BnID076	71.371	28.258	30.463	

A09	BoSF1814	105.283	28.319	30.526	
A09	BrSF6-2245	74.181	28.349	30.600	
A09	BrSF0389a	103.808	28.427	30.678	
A09	BrSF0353	103.205	28.431	30.682	
A09	BnSF400-28	95.854	28.525	30.767	
A09	BnSF400-29	102.491	28.526	30.768	
A09	BnSF400-54	103.619	28.553	30.801	
A09	BrSF6-2380	96.817	28.563	30.811	
A09	BnID078	108.166	28.565	30.816	
A09	BrSF6-2389	102.032	28.572	30.823	
A09	BrSF0251	102.032	28.572	30.823	
A09	CNU264	100.245	28.591	30.842	
A09	BrSF0383	100.245	28.591	30.842	
A09	BoSF0223	104.757	28.595	30.849	
A09	BrSF0250	105.283	28.599	30.851	
A09	BrSF6-2413	105.283	28.599	30.851	
A09	BnID079	107.200	28.604	30.856	
A09	BnID060	77.113	28.838	31.151	
A09	BrSF6-2562	76.400	28.872	31.190	
A09	BnSF400-253	76.762	28.889	31.207	
A09	BrSF0385	77.975	28.955	31.280	
A09	BnID081	79.084	29.012	31.319	
A09	ENA27	81.982	29.072	31.376	
A09	BnID082	82.907	29.117	31.416	
A09	BoSF0333	79.578	29.240	31.546	
A09	BoSF2709	81.756	29.270	31.580	
A09	BoSF2710	81.489	29.272	31.581	
A09	BrSF0229	117.952	29.898	32.003	
A09	BnID085	116.408	30.019	32.156	
A09	BnID061	112.358	30.171	32.308	

A09	BnID086	110.293	30.293	32.475	
A09	BnID087	111.058	30.311	32.493	
A09	BnID090	112.490	30.917	33.217	
A09	BnID062	114.603		33.611	
A09	BnID094	114.379		33.658	
A09	BnID063	113.425	31.076	33.837	
A09	BoSF1920a	112.630	31.209	33.970	
A09	BrSF0615b	116.971	31.249	34.010	
A09	BoSF0872b	116.971	31.249	34.010	
A09	BnID095	116.956	31.255	34.018	
A09	BnID097	124.713	31.582	34.337	
A09	BoSF2791	122.361	31.697	34.464	
A09	BoSF1885	120.657	31.931	34.645	
A09	BoSF2104a	120.869	32.190	34.980	
A09	BnEMS0337	121.670	32.700	35.490	
A09	BrSF77-118	123.262	32.867	35.719	
A09	BrSF0261	125.864	33.699	36.739	
A09	BoSF1902	125.794	33.992	37.032	
A09	BrSF82-562	125.127	34.017	37.058	
A10	BrSF0898a	0.000			
A10	BrSF0958	4.862	7.634	4.604	
A10	Na10E08	10.623	8.511	5.482	
A10	BrSF0436b	10.623	8.511	5.482	
A10	BoSF0652	26.195	11.468	10.030	
A10	sS2066	25.380	11.706	10.326	
A10	BrSF0287	26.843	11.853	10.480	
A10	niab140	26.843	11.853	10.480	
A10	BrSF0425a	30.494	12.330	10.939	
A10	sR12384	30.314	12.366	10.977	
A10	BrSF0283	33.626	12.721	11.405	

A10	niab122	36.032	12.984	12.936	
A10	BrSF0415	37.266	13.110	13.068	
A10	BoSF0601	36.732	13.155	13.121	
A10	niab103	44.075	13.713	13.673	
A10	BrGMS0536	44.921	13.797	13.761	
A10	BoSF1808	48.493	13.832	13.796	
A10	sN8502	47.179	13.930	13.895	
C01	BrSF0279	0.000			
C01	BoSF2670	4.551	1.902	2.160	
C01	CB10587	11.112	2.273	2.531	
C01	BoSF0224a	7.295	2.337	2.595	
C01	CNU139c	15.069	2.467	2.713	
C01	BoSF1469	18.174	3.122	3.256	
C01	BnEMS1012	25.893	5.805	5.715	
C01	niab068	29.242	5.878	5.793	
C01	IGF2026c	31.768	5.910	5.827	
C01	BoSF0678a	32.028	5.955	5.882	
C01	CB10369b	34.099	6.282	6.209	
C01	BoSF0442	34.425	6.301	6.228	
C01	BoSF1667	35.483	6.430	6.357	
C01	BoSF1998	35.730			
C01	BoGMS1565a	41.390	8.212	7.994	
C01	BoSF2182	41.390	8.212	7.994	
C01	BoSF0130	48.079	11.032	10.809	
C01	BoSF0133	50.843	11.296	11.072	
C01	BoSF0114	59.748	14.780	14.556	
C01	BrSF0811	65.108	34.475	17.941	Translocation
C01	BrSF0705	70.767	28.235	23.234	
C01	BoSF2314	68.799	28.302	23.293	
C01	BoSF2538	73.258	28.875	26.281	

C01	BoSF2346	70.906	29.505	26.911	
C01	BoSF0050b	75.799	29.818	27.224	
C01	BoSF1372	76.790		28.263	
C01	BoSF2575	78.684	31.345	29.274	
C01	ns258	91.292		31.652	
C01	ns059	94.081	34.106	32.358	
C01	ns262	94.526	34.744	32.996	
C01	BoSF1270	84.975	35.169	33.230	
C01	FITO283	99.531	38.103	37.881	
C01	BoSF2820	102.385	38.358	37.626	
C01	BoSF1597	99.082	38.740	38.517	
C02	BoSF1425	2.383	0.672	2.217	Inversion
C02	BoSF1799	1.926	0.754	2.123	Inversion
C02	BoSF2071	0.000	0.823	2.061	Inversion
C02	BnGMS0081	2.848	1.135	1.496	Inversion
C02	BoSF1539	3.200	1.305	1.465	Inversion
C02	BoSF2604	5.461	1.547	0.438	Inversion
C02	BoSF1017	4.453	1.727	0.258	Inversion
C02	BoSF1825	5.750	1.864	0.120	Inversion
C02	BoSF1826	5.875	1.870	0.115	Inversion
C02	Ol11H09	5.875	1.870	0.115	Inversion
C02	BoSF0132	5.078	1.912	0.073	Inversion
C02	BoGMS0665	3.780	1.973	0.012	Inversion
C02	CNU461	19.422	2.235	2.573	
C02	BoSF1264	28.264	3.755	4.093	
C02	BrSF0065b	30.135	3.822	4.160	
C02	niab124	31.164	4.298	4.612	
C02	BoSF2242	34.257	8.266	6.147	
C02	snap0673	39.120	8.569	7.209	
C02	snap0674	36.162	8.569	7.209	

C02	BoSF0033a	41.434	8.886	7.527	
C02	BoSF2475	52.109	14.032	12.673	
C02	BoSF0880	57.365	15.596	13.657	
C02	BnEMS0959	57.945	15.469	13.535	
C02	CB10026	61.496	16.325	14.391	
C02	BoSF0096	64.580	20.317	18.623	
C02	BoSF0757	66.240		19.800	
C02	BoSF1743	67.005		19.983	
C02	BoSF0525b	66.560		20.025	
C02	BoSF0284	74.930	36.101	34.048	
C02	ns314	90.565	36.166	34.122	
C02	ns085	96.901	38.749	37.524	
C02	BoSF1995a	84.397	39.286	37.965	
C02	BrSF0503	107.579	45.882	41.790	
C02	BoSF0310	110.008	46.086	41.995	
C02	BoGMS2128	111.156	46.361	42.269	
C02	BoSF1258	112.187			
C02	MD038	100.832			
C02	BoSF2269	113.694			
C03	BRMS006	8.932			
C03	BoSF0421	9.729	0.012		
C03	BoSF1944	9.472	0.282	0.037	
C03	BoSF2181	0.000	0.385	0.129	
C03	BoSF1005	6.468	0.473	0.220	
C03	BoSF1977	7.535	0.592	0.338	
C03	niab077	9.853	0.658	0.419	
C03	niab115b	10.755	1.174	1.025	
C03	BoSF2263	10.442	1.450	1.320	
C03	BoGMS0707	8.526	1.691	1.581	
C03	sR12137	4.549	1.924	1.811	

C03	BoSF2861	12.364	2.082	1.973	
C03	BoSF1510	13.000	2.085	1.976	
C03	BoGMS0407	17.301	2.840	2.210	
C03	BoSF0371	18.824	3.723	3.170	
C03	BoSF1970	25.467	4.128	3.589	
C03	BoSF2006	32.044	5.465	4.995	
C03	BoSF2005	32.172	5.475	5.000	
C03	BoSF2392b	30.528	5.525	5.059	
C03	BnID035a	35.832	5.896	5.621	
C03	BoGMS0678	34.789	5.923	5.647	
C03	BoSF0466	39.293	6.290	5.961	
C03	FITO218	38.778	6.650	6.321	
C03	BrSF0845b	43.310	7.126	6.798	
C03	BrBAC087b	42.175	7.152	6.877	
C03	BoSF2132	42.802	7.194	6.919	
C03	BN12A	43.312	7.481	7.162	
C03	CB10569	43.688	7.509	7.190	
C03	BoSF0805	44.656	7.603	7.335	
C03	CB10235	48.435	8.160	7.978	
C03	BoSF2258	49.504	8.909	8.422	
C03	BoSF2284	51.154	9.141	8.653	
C03	Na12E02b	56.596	9.667	9.180	
C03	ns027b	56.117	10.222	9.734	
C03	Ol10E05	59.661	11.161	9.848	
C03	BrSF0299	54.737	11.175	9.862	
C03	BoSF0670	58.050	11.175	9.862	
C03	BoSF1271b	61.416		10.386	
C03	snap0622	67.039	12.656	11.694	
C03	BoSF1276	63.809	12.705	11.746	
C03	BoSF0840	66.133	13.900	12.482	

C03	CB10057	81.272	15.395	14.250	
C03	BoGMS0369	82.632	15.561	14.407	
C03	BoSF0402	74.117	15.561	14.407	
C03	BoSF0160	73.187	16.193	15.076	
C03	BoSF2669	75.601	17.551	16.540	
C03	BoSF0883	76.845	17.836	17.209	
C03	BrGMS2766	79.345	18.806	18.179	
C03	BoSF1911	74.833	18.840	18.213	
C03	Na10E02	93.369	28.723	21.435	
C03	Na12G05	78.000	29.730	24.330	
C03	BoSF2281	92.053	31.058	28.247	
C03	snap0260	84.782	33.055	29.280	
C03	BoSF2723a	97.115	40.387	32.624	
C03	BoSF0176	110.499	46.516	39.908	
C03	BoSF2821	109.640	46.820	40.211	
C03	BoSF2233	109.441	46.859	40.251	
C03	BoSF2754	109.353	46.892	40.284	
C03	CALSSR	109.433	45.929	40.355	
C03	BoGMS0819	112.089	46.073	40.499	
C03	BoSF2509	108.722	47.486	43.713	
C03	BoSF2510	107.179	47.494	43.721	
C03	BoSF0711b	104.750	47.536	43.777	
C03	BoSF0926a	113.718	47.672	45.402	
C03	BrSF0193	116.309	47.683	45.413	
C03	BRAS068	120.910	50.536	47.708	
C03	MR049	123.296	50.720	47.523	
C03	Ol13C12	121.768	50.801	48.412	
C03	BoSF0960	130.195	55.354	50.621	
C03	snap0833	134.048	55.800	51.444	
C03	BoSF2513	135.654	57.463	54.442	

C03	BoSF1650	140.367	57.685	54.773	
C03	BrSF0214	148.684	57.689	54.776	
C03	BrSF0869	138.768	57.724	54.806	
C03	BoSF1136	144.371	57.761	54.844	
C04	BoSF1319	6.001	1.090	0.122	
C04	snap1060	0.000	1.662	0.722	
C04	BrSF0313	9.826	1.797	0.857	
C04	BoSF0349	17.181	2.326	1.774	
C04	BoSF1492	17.648	2.415	1.865	
C04	BoSF2273	15.282	3.586	2.380	
C04	CNU286	20.731	3.158	2.894	
C04	BoSF0643	20.731	3.158	2.894	
C04	FITO366	21.311	3.228	2.966	
C04	BoSF0016	21.936	3.246	2.982	
C04	BoGMS1219	23.443	3.324	3.040	
C04	BoSF1271a	24.631	3.750	3.465	
C04	BoSF0318	25.599	3.863	3.579	
C04	BnEMS0038	28.314	3.903	7.304	
C04	snap0205	40.458	4.541	6.649	
C04	BoSF1722	29.574	4.674	6.492	
C04	niab002	30.278	4.687	6.478	
C04	snap1299	42.534		7.959	
C04	BoSF2018	45.771	8.912	19.592	
C04	MR119	47.488	8.986	19.666	
C04	BoSF2723b	47.914			
C04	BoSF2884	53.611			
C04	BoSF0952a	53.827			
C04	BoSF1336	54.086	14.375		
C04	BoSF0552	55.436	14.566		
C04	CB10428	54.911	14.748		

C04	BoSF2040	55.617	14.932		
C04	BoSF0997	54.813	16.282	12.198	reshuffling
C04	BoSF0592	56.299	17.709	26.429	reshuffling
C04	BoSF2106	52.269	18.344	25.248	reshuffling
C04	IFLP013	56.108	18.541	25.052	reshuffling
C04	BoSF0718	56.168	18.942	24.651	reshuffling
C04	BoSF2062	56.037			reshuffling
C04	BoSF0600	56.175	18.884		reshuffling
C04	BoSF1121	56.937	18.941		reshuffling
C04	BoSF0949	56.707	19.490	24.163	reshuffling
C04	snap1891	45.005	19.967		reshuffling
C04	BoSF1852	55.809	19.980		reshuffling
C04	BoSF1843	56.177	20.324		reshuffling
C04	BoSF0943	57.346		11.694	reshuffling
C04	BrSF0552	56.694	21.693	15.257	reshuffling
C04	BoGMS2091	58.214	22.111	14.839	reshuffling
C04	BRAS061	58.960	22.255	14.611	reshuffling
C04	BoSF1692	62.175	23.382		reshuffling
C04	snap0299	80.189	30.412	28.276	reshuffling
C04	BoSF0935	70.221	30.447	27.785	reshuffling
C04	sN464	70.522	30.516	27.716	reshuffling
C04	ns104	73.778	30.956	27.276	reshuffling
C04	BoGMS1031	71.015	31.086	27.146	reshuffling
C04	BoSF2872	69.537	31.088	27.144	reshuffling
C04	BoSF0495	69.848	31.124	27.112	reshuffling
C04	BoGMS0282	76.843	34.290	28.536	
C04	BoSF2508	81.875	35.151	29.397	
C04	BoSF2294	84.360	36.174	30.420	
C04	ns126	94.751	36.517	30.763	
C04	ns123	89.755	36.591	30.837	

C04	BoSF2666	86.275	36.852	31.098	
C04	ns120	91.878	36.898	31.143	
C04	BoSF0613	71.737	40.860	31.744	
C04	BoSF0955	84.665	42.817	33.661	
C04	BnGMS0160b	105.115	45.654	38.055	
C04	BoSF2838	110.630	47.462	39.909	
C04	BoSF1937	123.433	48.115	40.444	
C04	MR155	116.650	48.403	40.511	
C04	Ol11H02	113.012	48.327	40.657	
C04	BoSF1009	118.907	48.636	40.966	
C04	snap1360	110.352	48.735	41.065	
C05	BnEMS1131	0.000	1.694	1.718	
C05	sORH13	5.448	2.248	2.031	
C05	BoSF2552	8.620	2.441	2.480	
C05	BoSF0017	10.293	2.529	2.567	
C05	BoSF2607	11.844	2.589	2.656	
C05	BoSF1212a	13.112	2.676	2.696	
C05	BoSF2676	24.230	4.226	25.541	Translocation
C05	BoGMS2688	26.693	4.328	25.457	Translocation
C05	BoSF1663	30.692	4.874	3.350	
C05	BoSF0022b	31.957	5.088	3.564	
C05	CB10374	39.347	6.949	6.064	
C05	BoSF0230	43.716	7.443	6.663	
C05	Na12C01	50.373	10.659	16.976	
C05	BrSF0858a	50.763	10.780	17.096	
C05	BoSF1969	52.018	12.576	17.714	
C05	BoSF2019	52.523	12.673	17.811	
C05	ns033	57.307			
C05	BoSF1918	58.273	16.685	11.982	Translocation
C05	BoSF0222	58.530			

C05	sS2129	59.222	24.453	7.208	Translocation
C05	Na10D11	59.685	24.453	7.208	Translocation
C05	sS2131	60.020	25.701	8.855	Translocation
C05	BrSF0040	65.605			
C05	CB10170	60.990	30.174	20.318	
C05	BRAS040	67.332	30.992	21.135	
C05	BoSF0926b	66.617	31.097	21.241	
C06	BoSF1665	16.453	1.430	2.715	Inversion
C06	BoSF0733	16.041	1.826	1.177	Inversion
C06	BoSF2316	17.657	2.958	0.045	Inversion
C06	ns337	0.000	3.219	14.644	
C06	ns326	6.954	3.220	14.644	
C06	BoSF0455b	16.005	3.439	15.253	
C06	snap0695b	30.252	12.456	30.949	Translocation
C06	BoSF0513	35.133	12.521	22.354	
C06	BoSF2395	35.624	12.714	22.161	
C06	BoSF1365	38.134	14.166		
C06	Na12E01b	42.790			
C06	snap1392	45.562	17.705		
C06	snap1395	41.822	17.754		
C06	BoSF1254	53.196	17.762		
C06	BoSF0952b	48.734	17.951	12.635	Translocation
C06	BrSF0784	51.508	17.991	12.676	Translocation
C06	BoSF0760	55.751	17.893	10.807	Translocation
C06	MR133.1	50.171	18.102	11.057	Translocation
C06	BoSF2506	54.529	18.766		
C06	BoSF2507	52.558	18.782		
C06	BoSF1126	59.954	20.394	24.496	
C06	BoSF2591	62.084	20.438	24.540	
C06	BoSF1406	76.301	29.433		

C06	sR12387	75.964	29.486		
C06	BoSF1144	76.919	29.502		
C06	BoSF1951	82.591	30.587	32.127	
C06	BoSF1468	77.696	31.158	32.698	
C06	BnEMS0350	85.058	31.726	33.266	
C06	CNU331a	93.683	34.190	37.415	
C06	Na10C06	99.642	34.268	37.425	
C06	ns347	109.710	34.497	37.645	
C07	CB10534	0.000	19.301		
C07	BoSF0778	8.179			
C07	BoSF2740	10.544	26.053	28.351	
C07	BoSF2771	11.880	26.105	28.435	
C07	BoSF1559	14.550	26.895	29.290	
C07	BoSF1324	18.413	27.954	30.200	
C07	BrSF46-176b	32.105	28.130	30.261	
C07	BoSF0783b	20.299	28.889	31.005	
C08	ns067	0.000	4.950	7.683	
C08	BoSF0521	13.627	5.460	8.193	
C08	BoGMS1419	10.797	5.715	8.448	
C08	BoGMS0456	17.543	14.103	11.950	
C08	BoSF0586a	16.909	14.437	12.284	
C08	BoSF2163	26.736	15.226	13.087	
C08	BoSF2274	18.771	15.779	13.721	
C08	BoSF2646	19.384	15.887	14.067	
C08	CB10053	19.920	15.990	14.170	
C08	BoSF1481	20.392	15.996	14.185	
C08	BoSF0554	21.849			
C08	BoSF2482	31.472	19.127	18.004	
C08	BnGMS0509	32.841	19.214	18.092	
C08	snap1382	36.239	19.654	18.249	

C08	BoSF2378	37.229	19.844	18.439	
C08	BoSF0023	38.467	19.959	18.543	
C08	BoSF2290	43.519	20.577	19.598	
C08	BoSF1926	56.797	25.513	27.397	
C08	BnEMS0020	53.391	25.537	27.421	
C08	BoSF2751	67.868	28.387	30.261	
C08	BoSF2347	66.166	28.805	30.671	
C08	BoSF2616	73.432	29.723	31.590	
C08	BoSF1222	74.824	29.991	31.839	
C08	BnSF287-186	80.555	31.123	33.033	
C08	niab129	81.512	31.176	33.096	
C08	BrSF0356	82.689	31.222	33.141	
C08	BrGMS0447	82.689	31.222	33.141	
C08	CB10179	85.290	31.401	33.365	
C08	IFLP021	92.111	33.288	35.630	
C08	BoSF0365	93.779	33.449	35.791	
C08	BnEMS1070	95.337	33.673	36.056	
C08	BoSF0448	98.532	33.921	36.345	
C08	BoSF1064	98.195	34.053	36.477	
C08	BoSF2073	100.968	34.348	36.862	
C08	BnEMS0606	103.818	34.941	37.507	
C08	sR5795	105.811	35.121	37.731	
C08	BN26A	107.213	35.293	37.900	
C08	BoSF1920b	107.181	35.299	37.907	
C08	BrSF0549a	107.955	35.354	37.962	
C08	CB10092	111.915	36.037	38.689	
C08	BrSF53-712	115.583	36.106	38.751	
C08	CB10028	112.222	36.352	39.012	
C09	BoSF0980	0.000	7.970		
C09	BoSF0336b	3.403			

C09	BrSF54-590	4.620	13.856	5.104	
C09	BoSF2578	5.757	13.859	5.107	
C09	BoSF0948	8.847	14.614	5.862	
C09	BnGMS0213	9.795	14.254	6.222	
C09	BoSF1228	12.542	14.788	6.756	
C09	BoSF2748	13.646	15.359	7.435	
C09	BoSF1976	12.860	15.415	7.490	
C09	BoSF2398	11.199	15.549	7.624	
C09	BoSF1872	14.037	16.505	8.400	
C09	BoSF1810	15.929	17.958	9.738	
C09	BoGMS1561	17.108	18.047	9.827	
C09	BoSF1788	17.108	18.047	9.827	
C09	BrSF1008	16.810	18.115	9.895	
C09	BoSF1817	18.121	18.368	10.149	
C09	BoGMS1453	20.538	18.868	11.104	
C09	BoSF0040	20.538	18.868	11.104	
C09	BoSF2393	22.837	32.027	19.941	
C09	BoSF2774	23.578	32.123	20.037	
C09	BrSF0436a	24.124	32.262	20.176	
C09	BoSF1647	22.397	32.391	20.452	
C09	BrSF0425b	41.175	41.614	31.085	
C09	BoSF0185	39.731	41.667	31.156	
C09	BrSF0369	45.689	41.868	31.405	
C09	Ol12A04	38.407	41.998	31.535	
C09	BoGMS1473	49.680	42.762	31.542	
C09	BoSF0483	44.177	43.110	32.435	

Table S5 ANOVA analysis of pod number and seed number per pod in five experiments.

**Trait**	**Source**	**DF**	**SS**	**MS**	**F value**	**P value**	***б*** ^{2}	***h*** ^{2}
PNb	Genotype	183	1352503	7391	2.0	3.7E-11	277.9	0.45
	Environment	3	2257494	752498	269.1	1.9E-118	1355.9	
	Genotype×Environment	469	1902498	4056	1.5	3.0E-06	420.2	
	Error	748	2091418	2796			2796.0	
PNm	Genotype	183	88726	485	4.9	7.9E-55	20.9	0.65
	Environment	4	246328	61582	816.2	1.2E-271	111.3	
	Genotype×Environment	646	110737	171	2.3	2.3E-27	32.0	
	Error	751	56666	75			75.5	
PNw	Genotype	183	1788677	9774	2.0	9.1E-11	400.4	0.49
	Environment	3	1588670	529557	152.5	4.0E-77	950.3	
	Genotype×Environment	469	2330885	4970	1.4	6.5E-06	499.3	
	Error	748	2597027	3472			3472.0	
SNPPb	Genotype	183	4388	24	9.6	6.4E-113	1.6	0.82
	Environment	3	1741	580	235.8	2.1E-107	1.0	
	Genotype×Environment	469	1978	4	1.7	2.7E-11	0.6	
	Error	741	1823	2			2.5	
SNPPm	Genotype	183	6243	34	13.2	2.4E-148	1.9	0.83
	Environment	4	10539	2635	1038.2	4.3E-303	4.8	
	Genotype×Environment	652	3819	6	2.3	2.6E-28	1.1	
	Error	746	1893	3			2.5	
SNPPw	Genotype	183	4522	25	11.7	6.4E-134	1.7	0.83
	Environment	3	2031	677	324.9	1.0E-134	1.2	
	Genotype×Environment	470	1940	4	2.0	3.7E-17	0.7	
	Error	743	1548	2			2.1	

Table S6A List of all detected QTLs at both significant and suggestive level for pod number and seed number per pod.

Trait_name	Experiments	Linkage_groups	Position	Interval	Additive_effect	Dominant effect	LOD_value	*R* ^2	Level
Pod number	W10F2:3m	A01	104.1	94.3-104.5	-4.31	2.24	3.0	12.6%	Significant
Pod number	X11F2:3m	A01	104.7	104.2-105.8	-5.48	2.79	3.0	12.5%	Significant
Pod number	W11F2:3b	A03	33.5	31.9-39.3	-7.75	7.52	2.8	2.8%	Suggestive
Pod number	W11F2:3w	A03	61.5	57.5-61.9	-9.65	7.90	3.6	3.0%	Significant
Pod number	W11F2:3m	A03	81.6	64.6-98	-1.73	-6.23	3.8	3.4%	Significant
Pod number	W09F2m	A05	10.8	4-13.8	-4.67	9.17	3.4	12.6%	Significant
Pod number	W10F2:3m	A06	86.3	82.8-92.6	-7.40	-2.70	9.9	23.9%	Significant
Pod number	W11F2:3m	A06	86.3	82.8-92.4	-6.70	-2.50	6.9	20.5%	Significant
Pod number	X11F2:3m	A06	86.3	82.6-93.0	-3.96	-1.48	7.7	24.0%	Significant
Pod number	X11F2:4m	A06	86.3	82.6-92.5	-6.09	-3.28	8.0	22.6%	Significant
Pod number	X11F2:3w	A09	44	34.5-46.4	4.02	-7.55	2.6	2.0%	Suggestive
Pod number	X11F2:3b	A09	44	37.8-46.1	4.09	-6.84	2.7	2.6%	Suggestive
Pod number	W11F2:3m	C02	5.1	3.2-17.4	-2.34	3.02	4.0	17.8%	Significant
Pod number	X11F2:3m	C02	9.8	5.5-21.5	-6.25	3.19	4.6	19.0%	Significant
Pod number	W10F2:3m	C02	9.8	5.8-18.2	-6.82	2.51	4.4	18.6%	Significant
Pod number	W09F2m	C02	107.6	100.4-113.6	-0.83	7.51	2.9	2.0%	Suggestive
Pod number	W11F2:3b	C03	11.4	5.9-12.6	-2.56	-8.49	2.7	2.1%	Suggestive
Pod number	X11F2:3w	C03	128.4	112-132.2	-6.34	-8.78	2.6	2.0%	Suggestive
Pod number	W10F2:3m	C06	51.2	49.1-53	5.78	-6.20	4.2	11.8%	Significant
Pod number	W11F2:3m	C06	51.2	49.4-53	8.80	-8.75	4.6	18.2%	Significant
Seed number per pod	X11F2:3b	A01	17.3	12.4-23.2	-1.01	-1.05	4.2	8.8%	Significant
Seed number per pod	X11F2:4b	A01	113.9	109.7-116.2	-0.83	1.03	4.1	3.1%	Significant
Seed number per pod	X11F2:4m	A01	113.9	106.6-117.7	-0.94	1.13	3.1	2.2%	Suggestive
Seed number per pod	X11F2:4w	A01	113.9	108.1-116.6	-0.89	1.09	4.0	2.9%	Significant

Seed number per pod	W10F2:3b	A02	6.1	0.4-12.8	0.28	0.77	4.2	3.7%	Suggestive
Seed number per pod	W10F2:3w	A02	6.1	0.7-17.6	0.44	0.75	5.2	6.9%	Significant
Seed number per pod	X11F2:4m	A02	21.1	19.9-23.3	-0.34	0.88	3.6	2.3%	Suggestive
Seed number per pod	X11F2:4b	A02	43.1	36.2-45.9	0.94	-0.59	4.4	6.5%	Significant
Seed number per pod	X11F2:4m	A02	43.1	37.8-45.2	1.27	-0.64	7.2	6.9%	Significant
Seed number per pod	X11F2:4w	A02	43.1	36.2-45.5	1.13	-0.68	5.9	7.7%	Significant
Seed number per pod	X11F2:4m	A02	81.6	79.7-82.9	0.24	0.08	3.9	3.9%	Significant
Seed number per pod	X11F2:4w	A02	81.6	80.2-82.9	0.40	0.04	4.6	4.6%	Significant
Seed number per pod	X11F2:3m	A03	14.1	12-20.1	-1.73	1.33	4.3	6.3%	Significant
Seed number per pod	X11F2:3b	A03	118	116-123.7	-0.92	1.67	3.3	5.6%	Suggestive
Seed number per pod	X11F2:3w	A03	118	116-121.9	-0.71	1.51	3.0	3.0%	Suggestive
Seed number per pod	X11F2:3m	A05	0	0-1.8	0.69	-1.59	3.9	3.5%	Suggestive
Seed number per pod	X11F2:4b	A05	8.8	4.2-13.7	-0.89	1.32	4.1	4.8%	Significant
Seed number per pod	X11F2:4w	A05	8.8	4.1-13.5	-1.04	1.48	4.4	5.3%	Significant
Seed number per pod	X11F2:4m	A05	10.8	6.8-14	-1.15	1.60	4.0	4.2%	Significant
Seed number per pod	W10F2:3b	A06	89.9	85.3-97.5	1.56	0.40	8.0	28.1%	Significant
Seed number per pod	W11F2:3b	A06	89.9	85.4-97.6	1.36	0.69	7.7	22.4%	Significant
Seed number per pod	X11F2:3b	A06	89.9	85.0-96.9	1.88	0.55	4.7	31.5%	Significant
Seed number per pod	X11F2:4b	A06	89.9	85.3-96.3	2.29	0.53	16.4	39.4%	Significant
Seed number per pod	W09F2m	A06	89.9	85.2-96.3	2.15	0.86	10.0	25.6%	Significant
Seed number per pod	W10F2:3m	A06	89.9	85.5-96.3	1.97	0.51	10.4	28.1%	Significant
Seed number per pod	W11F2:3m	A06	89.9	85.3-96.3	1.84	0.69	13.4	34.6%	Significant
Seed number per pod	X11F2:3m	A06	89.9	85.4-96.3	2.40	0.31	11.3	44.7%	Significant
Seed number per pod	X11F2:4m	A06	89.9	85.3-96.3	2.49	0.56	21.8	43.6%	Significant
Seed number per pod	W10F2:3w	A06	89.9	85.4-97.0	1.66	0.56	9.8	24.0%	Significant
Seed number per pod	W11F2:3w	A06	89.9	85.3-97.1	1.54	0.64	10.3	27.7%	Significant
Seed number per pod	X11F2:3w	A06	89.9	85.1-97.4	2.17	0.63	7.2	37.4%	Significant

Seed number per pod	X11F2:4w	A06	89.9	85.3-96.8	2.24	0.73	11.9	29.6%	Significant
Seed number per pod	W11F2:3b	A07	5.7	3.7-15.9	0.76	0.48	5.4	6.8%	Significant
Seed number per pod	W11F2:3w	A07	7.7	3.7-15.8	0.74	0.57	6.3	7.1%	Significant
Seed number per pod	X11F2:4b	A07	50.9	35.8-53.7	0.72	0.23	6.3	15.0%	Suggestive
Seed number per pod	W10F2:3b	A07	53.7	48.1-59.1	0.73	0.94	10.2	16.2%	Significant
Seed number per pod	W10F2:3w	A07	55.7	47.9-61.2	0.75	0.61	7.2	17.7%	Significant
Seed number per pod	W11F2:3b	A07	57.2	45.4-59.6	1.25	0.69	6.4	14.2%	Suggestive
Seed number per pod	W11F2:3m	A07	73.6	69.6-77.8	0.27	0.75	4.2	3.9%	Significant
Seed number per pod	W11F2:3w	A07	75.8	69.6-78.7	0.39	0.18	2.9	2.7%	Suggestive
Seed number per pod	W10F2:3m	A07	77.8	73.6-78.8	0.78	0.62	4.5	4.9%	Significant
Seed number per pod	X11F2:3b	A09	52	42.3-54.1	1.09	-0.50	3.6	3.4%	Suggestive
Seed number per pod	W11F2:3b	A09	68.5	62.5-74	0.66	0.17	3.9	5.8%	Significant
Seed number per pod	W11F2:3w	A09	68.9	62.5-76.2	0.52	0.13	3.2	4.0%	Suggestive
Seed number per pod	W11F2:3w	C02	5.8	2-18.1	-0.71	-0.13	3.8	6.7%	Significant
Seed number per pod	W11F2:3b	C06	49.6	47.8-58.2	0.75	0.73	3.6	3.9%	Suggestive
Seed number per pod	W11F2:3w	C06	57.2	48-58.2	0.68	0.04	3.8	5.5%	Significant
Seed number per pod	W10F2:3w	C06	13.5	11.5-17.5	0.98	-1.00	4.5	7.0%	Significant
Seed number per pod	W10F2:3b	C06	19.7	16.9-21.7	1.09	-1.04	4.7	10.2%	Significant

Table S6B List of identified QTLs after deleting non-reproducible suggestive QTLs for pod number and seed number per pod.

Identified_QTL	Trait_name	Experiments	Linkage_group	Position	Interval	Additive_effect	Dominant effect	LOD_value	*R* ^2	Level
qPN01	Pod number	W10F2:3m	A01	104.1	94.3-104.5	-4.31	2.24	3.0	12.6%	Significant
qPN02	Pod number	X11F2:3m	A01	104.7	104.2-105.8	-5.48	2.79	3.0	12.5%	Significant
Delelte	Pod number	W11F2:3b	A03	33.5	31.9-39.3	-7.75	7.52	2.8	2.8%	Suggestive
qPN03	Pod number	W11F2:3w	A03	61.5	57.5-61.9	-9.65	7.90	3.6	3.0%	Significant
qPN04	Pod number	W11F2:3m	A03	81.6	64.6-98	-1.73	-6.23	3.8	3.4%	Significant
qPN05	Pod number	W09F2m	A05	10.8	4-13.8	-4.67	9.17	3.4	12.6%	Significant
qPN06	Pod number	W10F2:3m	A06	86.3	82.8-92.6	-7.40	-2.70	9.9	23.9%	Significant
qPN07	Pod number	W11F2:3m	A06	86.3	82.8-92.4	-6.70	-2.50	6.9	20.5%	Significant
qPN08	Pod number	X11F2:3m	A06	86.3	82.6-93.0	-3.96	-1.48	7.7	24.0%	Significant
qPN09	Pod number	X11F2:4m	A06	86.3	82.6-92.5	-6.09	-3.28	8.0	22.6%	Significant
qPN10	Pod number	X11F2:3w	A09	44	34.5-46.4	4.02	-7.55	2.6	2.0%	Suggestive
qPN11	Pod number	X11F2:3b	A09	44	37.8-46.1	4.09	-6.84	2.7	2.6%	Suggestive
qPN12	Pod number	W11F2:3m	C02	5.1	3.2-17.4	-2.34	3.02	4.0	17.8%	Significant
qPN13	Pod number	X11F2:3m	C02	9.8	5.5-21.5	-6.25	3.19	4.6	19.0%	Significant
qPN14	Pod number	W10F2:3m	C02	9.8	5.8-18.2	-6.82	2.51	4.4	18.6%	Significant
Delelte	Pod number	W09F2m	C02	107.6	100.4-113.6	-0.83	7.51	2.9	2.0%	Suggestive
Delelte	Pod number	W11F2:3b	C03	11.4	5.9-12.6	-2.56	-8.49	2.7	2.1%	Suggestive
Delelte	Pod number	X11F2:3w	C03	128.4	112-132.2	-6.34	-8.78	2.6	2.0%	Suggestive
qPN15	Pod number	W10F2:3m	C06	51.2	49.1-53	5.78	-6.20	4.2	11.8%	Significant
qPN16	Pod number	W11F2:3m	C06	51.2	49.4-53	8.80	-8.75	4.6	18.2%	Significant
qSN01	Seed number per pod	X11F2:3b	A01	17.3	12.4-23.2	-1.01	-1.05	4.2	8.8%	Significant
qSN02	Seed number per pod	X11F2:4b	A01	113.9	109.7-116.2	-0.83	1.03	4.1	3.1%	Significant
qSN03	Seed number per pod	X11F2:4m	A01	113.9	106.6-117.7	-0.94	1.13	3.1	2.2%	Suggestive
qSN04	Seed number per pod	X11F2:4w	A01	113.9	108.1-116.6	-0.89	1.09	4.0	2.9%	Significant
qSN05	Seed number per pod	W10F2:3b	A02	6.1	0.4-12.8	0.28	0.77	4.2	3.7%	Suggestive
qSN06	Seed number per pod	W10F2:3w	A02	6.1	0.7-17.6	0.44	0.75	5.2	6.9%	Significant

Delelte	Seed number per pod	X11F2:4m	A02	21.1	19.9-23.3	-0.34	0.88	3.6	2.3%	Suggestive
qSN07	Seed number per pod	X11F2:4b	A02	43.1	36.2-45.9	0.94	-0.59	4.4	6.5%	Significant
qSN08	Seed number per pod	X11F2:4m	A02	43.1	37.8-45.2	1.27	-0.64	7.2	6.9%	Significant
qSN09	Seed number per pod	X11F2:4w	A02	43.1	36.2-45.5	1.13	-0.68	5.9	7.7%	Significant
qSN10	Seed number per pod	X11F2:4m	A02	81.6	79.7-82.9	0.24	0.08	3.9	3.9%	Significant
qSN11	Seed number per pod	X11F2:4w	A02	81.6	80.2-82.9	0.40	0.04	4.6	4.6%	Significant
qSN12	Seed number per pod	X11F2:3m	A03	14.1	12-20.1	-1.73	1.33	4.3	6.3%	Significant
qSN13	Seed number per pod	X11F2:3b	A03	118	116-123.7	-0.92	1.67	3.3	5.6%	Suggestive
qSN14	Seed number per pod	X11F2:3w	A03	118	116-121.9	-0.71	1.51	3.0	3.0%	Suggestive
Delelte	Seed number per pod	X11F2:3m	A05	0	0-1.8	0.69	-1.59	3.9	3.5%	Suggestive
qSN15	Seed number per pod	X11F2:4b	A05	8.8	4.2-13.7	-0.89	1.32	4.1	4.8%	Significant
qSN16	Seed number per pod	X11F2:4w	A05	8.8	4.1-13.5	-1.04	1.48	4.4	5.3%	Significant
qSN17	Seed number per pod	X11F2:4m	A05	10.8	6.8-14	-1.15	1.60	4.0	4.2%	Significant
qSN18	Seed number per pod	W10F2:3b	A06	88.9	85.3-97.5	1.56	0.40	8.0	28.1%	Significant
qSN19	Seed number per pod	W11F2:3b	A06	88.9	85.4-97.6	1.36	0.69	7.7	22.4%	Significant
qSN20	Seed number per pod	X11F2:3b	A06	88.9	85.0-96.9	1.88	0.55	4.7	31.5%	Significant
qSN21	Seed number per pod	X11F2:4b	A06	88.9	85.3-96.3	2.29	0.53	16.4	39.4%	Significant
qSN22	Seed number per pod	W09F2m	A06	88.9	85.2-96.3	2.15	0.86	10.0	25.6%	Significant
qSN23	Seed number per pod	W10F2:3m	A06	88.9	85.5-96.3	1.97	0.51	10.4	28.1%	Significant
qSN24	Seed number per pod	W11F2:3m	A06	88.9	85.3-96.3	1.84	0.69	13.4	34.6%	Significant
qSN25	Seed number per pod	X11F2:3m	A06	88.9	85.4-96.3	2.40	0.31	11.3	44.7%	Significant
qSN26	Seed number per pod	X11F2:4m	A06	88.9	85.3-96.3	2.49	0.56	21.8	43.6%	Significant
qSN27	Seed number per pod	W10F2:3w	A06	88.9	85.4-97	1.66	0.56	9.8	24.0%	Significant
qSN28	Seed number per pod	W11F2:3w	A06	88.9	85.3-97.1	1.54	0.64	10.3	27.7%	Significant
qSN29	Seed number per pod	X11F2:3w	A06	88.9	85.1-97.4	2.17	0.63	7.2	37.4%	Significant
qSN30	Seed number per pod	X11F2:4w	A06	88.9	85.3-96.8	2.24	0.73	11.9	29.6%	Significant
qSN31	Seed number per pod	W11F2:3b	A07	5.7	3.7-15.9	0.76	0.48	5.4	6.8%	Significant
qSN32	Seed number per pod	W11F2:3w	A07	7.7	3.7-15.8	0.74	0.57	6.3	7.1%	Significant
qSN33	Seed number per pod	X11F2:4b	A07	50.9	35.8-53.7	0.72	0.23	6.3	15.0%	Suggestive
qSN34	Seed number per pod	W10F2:3b	A07	53.7	48.1-59.1	0.73	0.94	10.2	16.2%	Significant

qSN35	Seed number per pod	W10F2:3w	A07	55.7	47.9-61.2	0.75	0.61	7.2	17.7%	Significant
qSN36	Seed number per pod	W11F2:3b	A07	57.2	45.4-59.6	1.25	0.69	6.4	14.2%	Suggestive
qSN37	Seed number per pod	W11F2:3m	A07	73.6	69.6-77.8	0.27	0.75	4.2	3.9%	Significant
qSN38	Seed number per pod	W11F2:3w	A07	75.8	69.6-78.7	0.39	0.18	2.9	2.7%	Suggestive
qSN39	Seed number per pod	W10F2:3m	A07	77.8	73.6-78.8	0.78	0.62	4.5	4.9%	Significant
Delelte	Seed number per pod	X11F2:3b	A09	52	42.3-54.1	1.09	-0.50	3.6	3.4%	Suggestive
qSN40	Seed number per pod	W11F2:3b	A09	68.5	62.5-74	0.66	0.17	3.9	5.8%	Significant
qSN41	Seed number per pod	W11F2:3w	A09	68.9	62.5-76.2	0.52	0.13	3.2	4.0%	Suggestive
qSN42	Seed number per pod	W11F2:3w	C02	5.8	2-18.1	-0.71	-0.13	3.8	6.7%	Significant
qSN43	Seed number per pod	W11F2:3b	C06	49.6	47.8-58.2	0.75	0.73	3.6	3.9%	Suggestive
qSN44	Seed number per pod	W11F2:3w	C06	57.2	48-58.2	0.68	0.04	3.8	5.5%	Significant
qSN45	Seed number per pod	W10F2:3w	C06	13.5	11.5-17.5	0.98	-1.00	4.5	7.0%	Significant
qSN46	Seed number per pod	W10F2:3b	C06	19.7	16.9-21.7	1.09	-1.04	4.7	10.2%	Significant

Table S6C List of consensus-QTL after the integration of reproducible identified-QTLs for pod number and seed number per pod, respectively.

Consensus QTL	Identified QTL	Experiment code	Linkage group	Peak Position	Confidence Interval	Additive effect	Dominant effect	LOD value	*R* ^2
	qPN01	W10F2:3m	A01	104.1	94.3-104.5	-4.31	2.24	3.0	12.6%
	qPN02	X11F2:3m	A01	104.7	104.2-105.8	-5.48	2.79	3.0	12.5%
qPN.A01-1		**W10F2:3m/X11F2:3m**	**A01**	**104.4**	**94.3-105.8**	**-4.90**	**2.52**	**3.0**	**12.5%**
qPN.A03-1	**qPN03**	**W11F2:3w**	**A03**	**61.5**	**57.5-61.9**	**-9.65**	**7.90**	**3.6**	**3.0%**
qPN.A03-2	**qPN04**	**W11F2:3m**	**A03**	**81.6**	**64.6-98**	**-1.73**	**-6.23**	**3.8**	**3.4%**
qPN.A05-1	**qPN05**	**W09F2m**	**A05**	**10.8**	**4-13.8**	**-4.67**	**9.17**	**3.4**	**12.6%**
	qPN06	W10F2:3m	A06	86.3	82.8-92.6	-7.40	-2.70	9.9	23.9%
	qPN07	W11F2:3m	A06	86.3	82.8-92.4	-6.70	-2.50	6.9	20.5%
	qPN08	X11F2:3m	A06	86.3	82.6-93.0	-3.96	-1.48	7.7	24.0%
	qPN09	X11F2:4m	A07	86.3	82.6-92.5	-6.09	-3.28	8.0	22.6%
qPN.A06-1		**W10F2:3m/W11F2:3m/X11F2:3m/X11F2:4m**	**A06**	**86.3**	**82.6-93.0**	**-6.04**	**-2.49**	**8.1**	**22.8%**
	qPN10	X11F2:3w	A09	44.0	34.5-46.4	4.02	-7.55	2.6	2.0%
	qPN11	X11F2:3b	A09	44.0	37.8-46.1	4.09	-6.84	2.7	2.6%
qPN.A09-1		**X11F2:3bw**	**A09**	**44.0**	**42.5-54.4**	**4.05**	**-7.20**	**2.7**	**2.3%**
	qPN12	W11F2:3m	C02	5.1	3.2-17.4	-2.34	3.02	4.0	17.8%
	qPN13	X11F2:3m	C02	9.8	5.5-21.5	-6.25	3.19	4.6	19.0%
	qPN14	W10F2:3m	C02	9.8	5.8-18.2	-6.82	2.51	4.4	18.6%
qPN.C02-1		**W10F2:3m/W11F2:3m/X11F2:3m**	**C02**	**8.2**	**3.2-21.5**	**-5.14**	**2.91**	**4.3**	**18.5%**
	qPN15	W10F2:3m	C06	51.2	49.1-53	5.78	-6.20	4.2	11.8%
	qPN16	W11F2:3m	C06	51.2	49.4-53	8.80	-8.75	4.6	18.2%
qPN.C06-1		**W10F2:3m/W11F2:3m**	**C06**	**51.2**	**49.1-53.0**	**7.29**	**-7.48**	**4.4**	**15.0%**
qSN.A01-1	**qSN01**	**X11F2:3b**	**A01**	**17.3**	**12.4-23.2**	**-1.01**	**-1.05**	**4.2**	**8.8%**
	qSN02	X11F2:4b	A01	113.9	109.7-116.2	-0.83	1.03	4.1	3.1%
	qSN03	X11F2:4m	A01	113.9	106.6-117.7	-0.94	1.13	3.1	2.2%
	qSN04	X11F2:4w	A01	113.9	108.1-116.6	-0.89	1.09	4.0	2.9%

qSN.A01-2		**X11F2:4bmw**	**A01**	**113.9**	**106.6-117.7**	**-0.89**	**1.09**	**3.7**	**2.7%**
	qSN05	W10F2:3b	A02	6.1	0.4-12.8	0.28	0.77	4.2	3.7%
	qSN06	W10F2:3w	A02	6.1	0.7-17.6	0.44	0.75	5.2	6.9%
qSN.A02-1		**W10F2:3bw**	**A02**	**6.1**	**0.4-17.6**	**0.36**	**0.76**	**4.7**	**5.3%**
	qSN07	X11F2:4b	A02	43.1	36.2-45.9	0.94	-0.59	4.4	6.5%
	qSN08	X11F2:4m	A02	43.1	37.8-45.2	1.27	-0.64	7.2	6.9%
	qSN09	X11F2:4w	A02	43.1	36.2-45.5	1.13	-0.68	5.9	7.7%
qSN.A02-2		**X11F2:4bmw**	**A02**	**43.1**	**36.2-45.9**	**1.11**	**-0.64**	**5.8**	**7.0%**
	qSN10	X11F2:4m	A02	81.6	79.7-82.9	0.24	0.08	3.9	3.9%
	qSN11	X11F2:4w	A02	81.6	80.2-82.9	0.40	0.04	4.6	4.6%
qSN.A02-3		**X11F2:4mw**	**A02**	**81.6**	**79.7-82.9**	**0.32**	**0.06**	**4.2**	**4.3%**
qSN.A03-1	**qSN12**	**X11F2:3m**	**A03**	**14.1**	**12-20.1**	**-1.73**	**1.33**	**4.3**	**6.3%**
	qSN13	X11F2:3b	A03	118.0	116.0-123.7	-0.92	1.67	3.3	5.6%
	qSN14	X11F2:3w	A03	118.0	116.0-121.9	-0.71	1.51	3.0	3.0%
qSN.A03-2		**X11F2:3bw**	**A03**	**118.0**	**116.0-123.7**	**-0.82**	**1.59**	**3.1**	**4.3%**
	qSN15	X11F2:4b	A05	8.8	4.2-13.7	-0.89	1.32	4.1	4.8%
	qSN16	X11F2:4w	A05	8.8	4.1-13.5	-1.04	1.48	4.4	5.3%
	qSN17	X11F2:4m	A05	10.8	6.8-14	-1.15	1.60	4.0	4.2%
qSN.A05-1		**X11F2:4bmw**	**A05**	**9.5**	**4.1-13.7**	**-1.03**	**1.47**	**4.1**	**4.7%**
	qSN18	W10F2:3b	A06	88.9	85.3-97.5	1.56	0.40	8.0	28.1%
	qSN19	W11F2:3b	A06	88.9	85.4-97.6	1.36	0.69	7.7	22.4%
	qSN20	X11F2:3b	A06	88.9	85.0-96.9	1.88	0.55	4.7	31.5%
	qSN21	X11F2:4b	A06	88.9	85.3-96.3	2.29	0.53	16.4	39.4%
	qSN22	W09F2m	A06	88.9	85.2-96.3	2.15	0.86	10.0	25.6%
	qSN23	W10F2:3m	A06	88.9	85.5-96.3	1.97	0.51	10.4	28.1%
	qSN24	W11F2:3m	A06	88.9	85.3-96.3	1.84	0.69	13.4	34.6%
	qSN25	X11F2:3m	A06	88.9	85.4-96.3	2.40	0.31	11.3	44.7%
	qSN26	X11F2:4m	A06	88.9	85.3-96.3	2.49	0.56	21.8	43.6%
	qSN27	W10F2:3w	A06	88.9	85.4-97	1.66	0.56	9.8	24.0%
	qSN28	W11F2:3w	A06	88.9	85.3-97.1	1.54	0.64	10.3	27.7%

	qSN29	X11F2:3w	A06	88.9	85.1-97.4	2.17	0.63	7.2	37.4%
	qSN30	X11F2:4w	A06	88.9	85.3-96.8	2.24	0.73	11.9	29.6%
qSN.A06-1		**W09F2m/W10F2:3bmw/W11F2:3bmw/X11F2:3bmw/X11F2:4bn**	**A06**	**88.9**	**85.0-97.6**	**2.00**	**0.60**	**11.2**	**32.4%**
	qSN31	W11F2:3b	A07	5.7	3.7-15.9	0.76	0.48	5.4	6.8%
	qSN32	W11F2:3w	A07	7.7	3.7-15.8	0.74	0.57	6.3	7.1%
qSN.A07-1		**W11F2:3bw**	**A07**	**6.7**	**3.7-15.9**	**0.75**	**0.53**	**5.9**	**6.9%**
	qSN33	X11F2:4b	A07	50.9	35.8-53.7	0.72	0.23	6.3	15.0%
	qSN34	W10F2:3b	A07	53.7	48.1-59.1	0.73	0.94	10.2	16.2%
	qSN35	W10F2:3w	A07	55.7	47.9-61.2	0.75	0.61	7.2	17.7%
	qSN36	W11F2:3b	A07	57.2	45.4-59.6	1.25	0.69	6.4	14.2%
qSN.A07-2		**W10F2:3bw/W11F2:3b/X11F2:4b**	**A07**	**54.4**	**35.8-61.1**	**0.86**	**0.62**	**7.5**	**15.8%**
	qSN37	W11F2:3m	A07	73.6	69.6-77.8	0.27	0.75	4.2	3.9%
	qSN38	W11F2:3w	A07	75.8	69.6-78.7	0.39	0.18	2.9	2.7%
	qSN39	W10F2:3m	A07	77.8	73.6-78.8	0.78	0.62	4.5	4.9%
qSN.A07-3		**W10F2:3m/W11F2:3mw**	**A07**	**75.7**	**69.6-78.8**	**0.48**	**0.52**	**3.9**	**3.8%**
	qSN40	W11F2:3b	A09	68.5	62.5-74	0.66	0.17	3.9	5.8%
	qSN41	W11F2:3w	A09	68.9	62.5-76.2	0.52	0.13	3.2	4.0%
qSN.A09-1		**W11F2:3bw**	**A09**	**68.7**	**68.5-82.2**	**0.59**	**0.15**	**3.6**	**4.9%**
qSN.C02-1	**qSN42**	**W11F2:3w**	**C02**	**5.8**	**2-18.1**	**-0.71**	**-0.13**	**3.8**	**6.7%**
	qSN43	W11F2:3b	C06	49.6	47.8-58.2	0.75	0.73	3.6	3.9%
	qSN44	W11F2:3w	C06	57.2	48-58.2	0.68	0.04	3.8	5.5%
qSN.C06-1		**W11F2:3bw**	**C06**	**53.4**	**47.8-58.2**	**0.72**	**0.39**	**3.7**	**4.7%**
	qSN45	W10F2:3w	C06	13.5	11.5-17.5	0.98	-1.00	4.5	7.0%
	qSN46	W10F2:3b	C06	19.7	16.9-21.7	1.09	-1.04	4.7	10.2%
qSN.C06-2		**W10F2:3bw**	**C06**	**16.6**	**82.9-97.7**	**1.03**	**-1.02**	**4.6**	**8.6%**

Table S7 List of QTLs identified in the current and previous studies for pod number and seed number per pod in rapeseed.

Referecne	Trait name	QTL name	Linkage group	Marker interval	Genomic region (Mb)	LOD value	R^2	Additive effect	Repeat number
Shi et al. 2009	PNw	*qPN.A1-1*	A01	CB10097 / niab096	1.6-2.0	5.0	7.7	-23.60	1
Shi et al. 2009	PNw	*qPN.A1-2*	A01	pW145 / S15M04-2-330	2.2-2.9	5.5	8.6	-24.8	1
Shi et al. 2009	PNw	*qPN.A1-3*	A01	sR11990a / RA2G09	3.0-5.5	4.4	7	-22.3	1
Shi et al. 2009	PNw	*qPN.A1-4*	A01	CB10095 / CNU235	5.9-6.8	2.7-7.9	4.7-12.2	-	2
Current study	PN	*qPN.A01-1*	A01	BrSF0706 / TFZIP23	15.3-18.3	3.0	12.5	-4.90	2
Ding et al. 2012	PNw	*PNLP-A1*	A01	MR38 / niab056	22.2-24.5	/	13.7	+	2
Shi et al. 2009	PNw	*qPN.A2-1*	A02	HUA133 / P10M8-130	0.1-/	2.9-3.8	4.6-6	-	2
Ding et al. 2012	PNw	*PNLP-A2*	A02	B070J05 / Na14H11	2.4-4.5	/	10.4	-	1
Wang et al. 2010	PNw	*qSNP2*	A02	ME6EM4b / ME14EM5b	/-/	2.5	35.64	171.89	1
Shi et al. 2009	PNw	*qPN.A3-1*	A03	FLC3 / pw189	1.3-2.7	2.4-3.8	3.4-6.2	±	2
Ding et al. 2012	PNw	*PNLP-A3*	A03	BoGMS0230 / BnGMS257	5.1-6.1	/	9.2	-	1
Shi et al. 2013	PNw	*qPN-LP2-A3b*	A03	MR123 / BRMS043	5.1-7.4	4.5	8.6	+	1
Radoev et al. 2008	PNw	*Sil/dm^2_N3*	A03	E32M51_283E / MR12	10.8-13.2	10.7	12	2.88	1
Current study	PN	*qPN.A03-1*	A03	BrSF0295 / CNU098	11.4-13.5	3.6	3.0	-9.65	1
Current study	PN	*qPN.A03-2*	A03	FITO285 / BoSF2828	14.2-15.4	3.8	3.4	-1.73	1
Shi et al. 2009	PNw	*qPN.A3-2*	A03	SA29 / CNU276	14.0-15.2	3.4	6.4	25.10	1
Shi et al. 2013	PNw	*qPN-LP1-A3/qPN-LP2-A3c/qPN-OP1-A3a*	A03	CNU002 / CNU370	16.1-20.4	3.6	7.3	+	3
Shi et al. 2013	PNw	*qPN-OP1-A3b*	A03	B072K21-4 / CNU371	18.7-20.8	3.8	8.0	+	1
Shi et al. 2013	PNw	*qPN-OP2-A3*	A03	HBr033 / BnSQD1-A3	23.7-27.3	3.7	7.8	+	1
Shi et al. 2013	PNw	*qPN-LP2-A3a*	A03	pw189 / HBr061	43.5-45.0	3.4	6.6	+	1
Shi et al. 2009	PNw	*qPN.A5-1*	A05	sNRD03 / P01M22-200	0.0-0.8	4.1	4.4	12.90	1
Ding et al. 2012	PNw	*PNNP-A5*	A05	BnPHT1 / BoGMS1565c	3.3-4.0	/	8.9	-	1
Shi et al. 2009	PNw	*qPN.A5-2*	A05	E1HM31-130 / CNU344	3.6-4.6	4.5	8	38.30	1
Current study	PN	*qPN.A05-1*	A05	BrSF0712 / BoSF2141	6.0-8.0	3.4	12.6	-4.67	1
Shi et al. 2009	PNw	*qPN.A5-3*	A05	sR9222 / Na10B08	10.4-11.2	4.6	6.7	35.30	1

Shi et al. 2009	PNw	*qPN.A5-4*	A05	CNU206 / pW161-2b	12.5-13.2	3.7	6.2	33.00	1
Shi et al. 2009	PNw	*qPN.A5-5*	A05	Na10E02 / CNU268	19.1-20.1	3.3	4.9	18.30	1
Shi et al. 2009	PNw	*qPN.A6-1*	A06	IGF1075d / niab37	7.3-11.1	3.2-3.4	3.5-5	±	2
Ding et al. 2012	PNw	*PNLP-A6*	A06	H001J23 / BnGMS531	8.5-12.5	/	13.3	-	1
Current study	PN	*qPN.A06-1*	A06	BrSF47-234 / BrSF0166	22.0-23.1	5.1	22.8	-6.02	4
Radoev et al. 2008	PNw	*Sil/dm*^{2}*_N7*	A07	CB10439 / MR153b	10.5-13.5	7.3	8.8	2.64	1
Shi et al. 2009	PNw	*qPN.A7-1*	A07	E1HM34-220 / 35RXTRAP10-4	11.0-12.9	2.6-3.3	4.6-6.2	±	2
Yi et al. 2006	PNw	*NPP1-LG4*	A07	M9E12750 / Ra2A01	/-14.5	4.3	17.42	19.95	1
Shi et al. 2009	PNw	*qPN.A7-2*	A07	niab063 / sR7223	14.1-14.4	2.5-2.7	4.9-5.4	±	2
Shi et al. 2009	PNw	*qPN.A7-3*	A07	IGF3309b / P2M8-110	20.1-21.3	2.9	5.6	-17.70	1
Shi et al. 2009	PNw	*qPN.A7-4*	A07	WRI1-2 / CNU168	21.1-22.0	3.5-6.9	6.5-13.7	-	2
Shi et al. 2009	PNw	*qPN.A7-5*	A07	FTin1-a / IGF1226a	23.3-23.8	2.8-3.8	5.2-8	-	2
Wang et al. 2010	PNw	*qSNP8*	A08	ME17EM8c / Na12B05a	/-15.4	2.7	12.96	−8.18	1
Ding et al. 2012	PNw	*PNLP-A8*	A08	em15me1b / em8me2	/-/	/	11.5	-	1
Radoev et al. 2008	PNw	*Sil/dm*^{2}*_LG10*	A08	E34M47_185R / E45M60_234E	/-/	7.7	4.4	1.75	1
Ding et al. 2012	PNw	*PNNP-A9b*	A09	HBr304 / MR13	3.0-4.2	/	10.7	+	1
Ding et al. 2012	PNw	*PNNP-A9a*	A09	HBr303 / BnPAP11	3.3-4.8	/	18.4	+	1
Shi et al. 2009	PNw	*qPN.A9-1*	A09	FTin1-b / E8HM40-510	20.9-21.8	4.2	5.8	-24.80	1
Current study	PN	*qPN.A09-1*	A09	BrSF0998 / BrSF6-104	22.8-24.2	2.7	2.3	4.05	2
Shi et al. 2009	PNw	*qPN.A9-2*	A09	niab131 / CNU008	29.8-30	3.1	4.7	17.90	1
Shi et al. 2013	PNw	*qPN-LP2-A9*	A09	H091P21-4 / pW123aH	31.2-32.5	4.9	9.5	+	1
Shi et al. 2013	PNw	*qPN-LP1-A9*	A09	HBr075 / H091P21-4	32.5-32.8	4.2	9.1	+	1
Radoev et al. 2008	PNw	*Sil/dm*^{2}*_N9*	A09	CB10533a / MR230	32.7-33.0	6.4	1.9	-1.15	1
Shi et al. 2009	PNw	*qPN.A10-2*	A10	E5HM31-490 / IGF5108e	16.2-17.7	2.6-7.7	5.9-12.2	±	3
Ding et al. 2012	PNw	*PNNP-C1*	C01	CB10369 / BoGMS0806	6.3-8.3	/	9.2	-	1
Wang et al. 2010	PNw	*qSNP11a*	C01	Me9EM10c / Ol12F11a	/-11.1	2.8	28.71	159.25	1
Wang et al. 2010	PNw	*qSNP11b*	C01	Na12C08 / ME16EM2a	21.9-/	2.6	34.02	141.05	1
Radoev et al. 2008	PNw	*Sil/dm*^{2}*_N11*	C01	MD60a / Ol10E12	25.0-18.5	4.7	2.9	1.40	1
Shi et al. 2009	PNw	*qPN.C1-1*	C01	E1HM34-150 / P13M6-90	/-/	4.3	8.1	33.50	1

Current study	PN	*qPN.C02-1*	C02	BoSF1539 / CNU461	1.3-2.5	4.3	13.5	-5.14	3
Radoev et al. 2008	PNw	*Sil/dm ^{2} _N12b*	C02	E35M62_117E / Na12E04b	/-4.5	14.3	15	3.22	1
Shi et al. 2009	PNw	*qPN.C2-2*	C02	sR6293b / CB10530b	9.2-11.2	3.4-4.7	6.4-8.5	+	2
Radoev et al. 2008	PNw	*Sil/dm ^{2} _N12a*	C02	CB10022a / MR104	41.8-45.4	7.7	6.1	-1.79	1
Shi et al. 2009	PNw	*qPN.C2-1*	C02	S13-50 / P04M21-490	/-/	4.9	10.3	15.80	1
Shi et al. 2009	PNw	*qPN.C2-3*	C02	Na12C12 / 21FXTRAP10-1	/-/	4.0	9.3	-22.40	1
Ding et al. 2012	PNw	*PNNP-C3*	C03	niab115 / BoGMS1288	2.2-3.3	/	9.5	-	1
Shi et al. 2013	PNw	*qPN-LP3-C3a*	C03	BRMS106 / pX141aE	3.9-6.7	3.4	7.4	-	1
Shi et al. 2009	PNw	*qPN.C3-1*	C03	STS01 / pX141bE	/-6.0	3.7	4.3	-19.20	1
Shi et al. 2013	PNw	*qPN-LP3-C3b*	C03	HG-FLC-C3b / HBr056	14.1-20.0	5.5	12.3	+	1
Shi et al. 2009	PNw	*qPN.C3-2*	C03	Ol11G11b / pW143	21.4-23.7	2.4-3.3	4.9-6.1	+	2
Shi et al. 2009	PNw	*qPN.C3-3*	C03	IGF3165b / CNU099	24.1-28.8	7.0	12	28.30	1
Shi et al. 2009	PNw	*qPN.C3-4*	C03	IGF1141f / E7HM31-450	30.3-/	5.7	9.3	25.10	1
Shi et al. 2009	PNw	*qPN.C5-1*	C05	P10M10-330 / CB10124	0.4-1.0	2.9-2.9	4.4-4.8	+	2
Shi et al. 2009	PNw	*qPN.A10-1*	C05	Na10D11 / P11M10-140	/-24.4	3.1-3.9	6.5-6.9	+	2
Current study	PN	*qPN.C06-1*	C06	BoSF1254 / BoSF2507	17.9-18.8	4.4	15.0	7.29	2
Ding et al. 2012	PNw	*PNNP-C6*	C06	BnGMS118 / MR133.1	18.1-18.9	/	13.2	-	2
Shi et al. 2009	PNw	*qPN.C6-1*	C06	PA28 / rFT2-a	28.6-30.8	2.9	5.8	-11.90	1
Radoev et al. 2008	PNw	*Sil/dm ^{2} _N16*	C06	CB10211b / CB10632	33.7-36.3	6.6	5.7	1.98	1
Ding et al. 2012	PNw	*PNNP-C8*	C08	BnGMS319 / HBr150	27.7-28.5	/	8.1	+	1
Ding et al. 2012	PNw	*PNLP-C8*	C08	CNU296 / HBr123	29-30.4	/	9	+	1
Ding et al. 2012	PNw	*PNLP-C9*	C09	BoGMS0989 / e3m9a	2.3-/	/	12	+	1
Shi et al. 2009	PNw	*qPN.C9-1*	C09	SA30 / S15M04-2-92	28.0-/	7.5	11.6	28.90	1
Shi et al. 2009	PNw	*qPN.C9-2*	C09	E2HM34-260 / Na12G04	/-33.5	5.9	9.4	31.20	1
Shi et al. 2009	PNw	*qPN.C9-3*	C09	Na10D07b / S05M05-130	39.4-41.7	3.0-3.0	5.4-5.5	+	2
Shi et al. 2013	PNw	*qPN-OP3-C9*	C09	HR-Sp1-170 / pw155a	41.7-43.6	4.3	10.0	-	1
Current study	SNPP	*qSN.A01-1*	A01	BoSF2770 / niab106	0.5-1.5	4.2	8.8	-1.01	1
Shi et al. 2009	SNPPw	*qSN.A1-1*	A01	niab71 / CNU142	0.7-1.5	2.4-3.9	4-6.1	±	2
Shi et al. 2009	SNPPw	*qSN.A1-2*	A01	niab096 / EST156a	2.0-2.1	2.7-8.1	4.7-12.2	+	2

Shi et al. 2009	SNPPw	*qSN.A1-3*	A01	sR11990a / RA2G09	4.0-5.5	2.5-2.8	5.5-5.8	+	2
Shi et al. 2009	SNPPw	*qSN.A1-4*	A01	E5HM31-505 / S10M16-2-130	/-/	4.9-6.7	8.8-11.4	+	2
Shi et al. 2009	SNPPw	*qSN.A1-5*	A01	CNU235 / Ol12F11	6.6-7.0	5.5-7.1	9.5-10	+	2
Shi et al. 2009	SNPPw	*qSN.A1-7*	A01	E10HM34-230 / EST165	/-8.7-/	4.3-5.5	4.2-9.7	+	2
Qi et al. 2014	SNPPm	*qSNA1*	A01	Na14G06 / CB10189	8.2-8.9	/	23.54	1.93	3
Shi et al. 2009	SNPPw	*qSN.A1-6*	A01	IGF2071e / EST98b	/-17.5	3-9.1	4.3-12.4	+	5
Current study	SNPP	*qSN.A01-2*	A01	TFZIP23 / BrSF0982	18.3-19.6	3.7	2.7	-0.89	2
Wang et al. 2010	SNPPw	*qSS1*	A01	ME6EM8d / ME3EM8a	/-/	2.9	8.41	−3.6	1
Current study	SNPP	*qSN.A02-1*	A02	niab025 / MR052	0.1-1.8	4.7	5.3	0.36	1
Shi et al. 2013	SNPPw	*qSN-OP1-A2b/qSN-OP2-A2*	A02	BnSIZ1-A2 / BnMGD2-A2b	2.0-3.0	7.9	14.5	-	2
Shi et al. 2013	SNPPw	*qSN-OP3-A2/qSN-OP1-A2a/qSN-LP3-A2*	A02	sR6293a / BnSIZ1-A2	3.0-4.7	4.5	9.4	-	3
Ding et al. 2012	SNPPw	*SNLP-A2*	A02	Na14H11 / B049H14	4.2-4.5	/	11.2	-	1
Current study	SNPP	*qSN.A02-2*	A02	BrSF0755 / BoSF2100	6.0-8.3	5.8	7.0	1.11	3
Shi et al. 2009	SNPPw	*qSN.A2-1*	A02	pX154 / pX155	10.2-10.3	10.2	15.5	-1.81	1
Shi et al. 2013	SNPPw	*qSN-LP1-A2a/qSN-LP1-A2b/qSN-LP2-A2*	A02	ZAAS113 / B084M08a	10.3-13.1	3.7	6.8	-	3
Current study	SNPP	*qSN.A02-3*	A02	BoSF2214 / Na14B05	22.4-22.7	4.2	4.3	0.32	2
Shi et al. 2009	SNPPw	*qSN.A2-2*	A02	S10M16-2-210 / S10M03-1-280	/-/	3.0	5.5	-0.98	1
Current study	SNPP	*qSN.A03-1*	A03	BrSF0427 / BoSF2392a	1.5-4.1	4.3	16.3	-1.73	1
Shi et al. 2013	SNPPw	*qSN-LP3-A3b*	A03	HBr049 / CNU098	12.8-13.5	4.2	8.0	+	1
Shi et al. 2009	SNPPw	*qSN.A3-1*	A03	CNU210 / S13M08-1-157	15.3-15.7	2.3-3.9	4.5-5.6	±	2
Ding et al. 2012	SNPPw	*SNLP-A3*	A03	CNU270 / HBr131	15.4-16.2	/	11.4	-	1
Current study	SNPP	*qSN.A03-2*	A03	BrSF0708 / CB10425	23.3-24.6	3.1	4.3	-0.82	1
Ding et al. 2012	SNPPw	*SNNP-A3*	A03	BoGMS1539 / BoGMS0408	25.5-27.3	/	16.1	+	1
Shi et al. 2009	SNPPw	*qSN.A4-2*	A04	IGF3395d / IGF3391a	0.0-0.5	6.7-7.5	8.5-12.1	-	2
Shi et al. 2013	PNw	*qPN-LP3-A4*	A04	BnLPR2-A4 / znS13M26-220	4.3-8.7	3.5	7.5	+	1
Shi et al. 2009	SNPPw	*qSN.A4-3*	A04	HUA39 / CNU360	15.9-16.2	2.7-3	5.6-6.1	+	2
Shi et al. 2009	SNPPw	*qSN.A4-4*	A04	CNU256 / E9HM31-80	16.2-17.2	4.5-8.1	7.7-13.2	+	2
Shi et al. 2009	SNPPw	*qSN.A4-5*	A04	E10HM34-180 / S14M08-2-270	/-/	3-3.3	3.7-4.1	±	2
Shi et al. 2009	SNPPw	*qSN.A4-1*	A04	E6HM31-400 / P7M1-275	/-/	3.3-7	6.1-14	+	2
Shi et al. 2009	SNPPw	*qSN.A5-1*	A05	CNU257 / BRAS063	3.2-3.3	3.9	7.6	-0.54	1

Shi et al. 2013	PNw	*qPN-OP1-A5*	A05	BnPHT1-A5 / sN12353b	3.2-4.4	3.4	6.6	+	1
Current study	SNPP	*qSN.A05-1*	A05	BrSF0712 / BoSF2141	5.8-7.8	4.1	4.7	-1.03	3
Radoev et al. 2008	SNPPm	*S/Sil_N5*	A05	MD21 / MR113	8.0-12.4	14.1	11.8	-1.55	1
Shi et al. 2009	SNPPw	*qSN.A5-2*	A05	CNU206 / pW161-2b	11.2-12.8	3.9	6	-0.78	1
Wang et al. 2010	SNPPw	*qSS5*	A05	ME1EM6b / ME7EM6c	/-/	2.4	7.87	−5.38	1
Cai et al. 2014	SNPPm	*UN*	A06	BnGMS583	/-16.8-/	3.6	4.8	0.7	1
Current study	SNPP	*qSN.A06-1*	A06	BrSF0819 / BrSF0144	22.6-23.7	11.0	32.1	1.97	13
Ding et al. 2012	SNPPw	*SNLP-A6*	A06	BnGS1 / em13me5	/-/	/	13	+	2
Shi et al. 2009	SNPPw	*qSN.A7-1*	A07	CNU063 / RA2G08	0.8-5.2	3.3	5.6	0.62	1
Current study	SNPP	*qSN.A07-1*	A07	BoSF2344 / AT1G27050	5.0-8.9	5.9	6.9	0.75	2
Cai et al. 2014	SNPPm	*UN*	A07	BrGMS4507	/-10.3-/	3.1	5.2	1.38	1
Current study	SNPP	*qSN.A07-2*	A07	CN64 / CNU339	15.0-20.1	6.0	10.8	0.86	4
Current study	SNPP	*qSN.A07-3*	A07	BoGMS2110 / CNU053	20.8-22.5	3.9	3.8	0.48	3
Qi et al. 2014	SNPPm	*qSNA7*	A07	T10 / EA2MG2a	/-/	/	3.75	0.77	3
Zhang et al. 2011	SNPPm	*qSS.N8-1*	A08	EA02MG05-210 / CB10026	7.2-9.2	4.4	12.23	-3.10	3
Ding et al. 2012	SNPPw	*SNLP-A8*	A08	H012K23 / BnPAP25	12.0-12.7	/	9.4	+	1
Qi et al. 2014	SNPPm	*qSNA8*	A08	niab050 / Lat21-2	16.5-17.6	/	5.51	-0.93	3
Shi et al. 2009	SNPPw	*qSN.A8-1*	A08	CNU489 / P7M5-170	/-/	2.6-3.3	4.4-5.2	-	2
Zhang et al. 2011	SNPPm	*qSS.N8-3*	A08	SA06TC14-270 / S77096	/-/	2.9	9.63	2.73	1
Zhang et al. 2011	SNPPm	*qSS.N8-4*	A08	SA06TC14-270 / S77096	/-/	2.5	8.35	2.83	2
Zhang et al. 2011	SNPPm	*qSS.N8-2*	A08	SA10TC11-100 / SA03TC09-370	/-/	2.9	10.6	4.07	3
Shi et al. 2009	SNPPw	*qSN.A9-1*	A09	P04M21-130 / IGF5222b	1.0-1.8	4.1	6.5	0.77	1
Ding et al. 2012	SNPPw	*SNNP-A9*	A09	HBr199c / HBr097c	1.2-1.8	/	10.5	-	2
Shi et al. 2013	SNPPw	*qSN-LP1-A9b*	A09	IGF5385f / IGF5222b	1.8-2.0	4.7	8.4	+	1
Shi et al. 2009	SNPPw	*qSN.A9-2*	A09	CB10311 / pW203b	7.2-8.6	4.1	5.4	0.76	1
Shi et al. 2013	SNPPw	*qSN-LP1-A9a/qSN-OP2-A9*	A09	H055O17-4 / HBr096	25.1-29.8	3.9	6.9	+	2
Current study	SNPP	*qSN.A09-1*	A09	BnSF2342-39 / BnID082	28.1-29.1	3.6	4.9	0.59	2
Shi et al. 2009	SNPPw	*qSN.A9-3*	A09	niab33 / pW123aH	30.0-31.2	3.0	5.6	-0.47	1
Qi et al. 2014	SNPPm	*qSNA9*	A09	CNU034 / niab038	/-/	/	2.73	-0.66	3
Ding et al. 2012	SNPPw	*SNLP-A10*	A10	Na10D07 / S002B15-1	10.5-11.5	/	12.8	+	1

Ding et al. 2012	SNPPw	*SNLP-C1*	C01	CB10369 / BoGMS0806	6.3-8.3	/	11.9	+	1
Wang et al. 2010	SNPPw	*qSS11*	C01	Ol12F11a / ME5EM9c	10.1-12.1	3.0	24.37	−5.51	1
Shi et al. 2013	SNPPw	*qSN-LP2-C1*	C01	ZAAS156b / znS08M15-80	19.1-22.0	3.6	7.1	-	1
Shi et al. 2013	SNPPw	*qSN-OP2-C1*	C01	BnGPT2-C1 / pW190b	28.3-29.3	3.5	7.5	-	1
Shi et al. 2013	SNPPw	*qSN-OP1-C1*	C01	HR-Sp2-170 / BRMS175	36.5-38.8	3.7	6.3	-	1
Radoev et al. 2008	SNPPm	*S/Sil_N11*	C01	CB10536 / CB10357b	35.3-37.7	15.6	8.2	-1.49	1
Current study	SNPP	*qSN.C02-1*	C02	BoSF1425 / CNU461	0.8-2.2	3.8	6.7	-0.71	1
Shi et al. 2009	SNPPw	*qSN.C2-1*	C02	pW119 / sR12095	1.4-1.7	2.6-3.4	3.3-4.7	+	2
Ding et al. 2012	SNPPw	*SNNP-C3*	C03	BoGMS0576 / MR123	9.1-10.0	/	17.3	+	1
Qi et al. 2014	SNPPm	*qSNC3*	C03	t8_at_3 / CNU270	23.2-/	/	5.12	0.90	3
Shi et al. 2009	SNPPw	*qSN.C3-1*	C03	IGF1141f / E7HM31-450	30.3-/	8.6	10.8	-1.43	1
Zhang et al. 2011	SNPPm	*qSS.N13-2*	C03	EA02MC05-160 / EA13MC01-160	/-/	3.4	9.7	-2.31	2
Zhang et al. 2011	SNPPm	*qSS.N13-1*	C03	EA02MC05-160 / EA13MC01-160	/-/	5.8	15.21	-3.21	2
Ding et al. 2012	SNPPw	*SNNP-C5*	C05	BnGMS352 / BnGMS309	15.3-16.0	/	11.5	-	1
Ding et al. 2012	SNPPw	*SNLP-C5*	C05	Ol10A10 / sS2129	24.5-25.7	/	14.7	+	1
Shi et al. 2009	SNPPw	*qSN.C6-4*	C06	HUA64-2 / Na12E01a	16.9-18.1	4.3	6.9	0.88	1
Current study	SNPP	*qSN.C06-1*	C06	BoSF1254 / BoSF1126	17.8-19.0	3.7	4.7	0.72	2
Shi et al. 2009	SNPPw	*qSN.C6-3*	C06	CB10010 / IGF3380b	20.2-20.5	4.1-4.2	3.6-7.7	+	2
Shi et al. 2009	SNPPw	*qSN.C6-2*	C06	E5HM40-205 / BRMS015	20.6-21.1	3.9	6.8	0.62	1
Shi et al. 2009	SNPPw	*qSN.C6-1*	C06	pW134 / AP1a	29.6-32.0	3.9	6.8	0.71	1
Current study	SNPP	*qSN.C06-2*	C06	BoSF1951 / CNU331a	30.6-34.2	4.6	8.6	1.03	2
Shi et al. 2013	SNPPw	*qSN-OP3-C6b/qSN-LP1-C6*	C06	BnPHT1-C6 / PA28	34.4-36.0	4.2	8.6	+	2
Qi et al. 2014	SNPPm	*qSNC6*	C06	CNU182 / CNU052	34.8-35.9	/	0.78	0.35	3
Shi et al. 2013	SNPPw	*qSN-LP3-C6/qSN-OP3-C6a*	C06	IGF1226b / BnPHT1-C6	36.1-36.9	4.0	9.2	+	2
Wang et al. 2010	SNPPw	*qSS16a*	C06	ME6EM4d / ME3EM4b	/-/	2.6	8.57	−5.85	1
Wang et al. 2010	SNPPw	*qSS16b*	C06	ME6EM4d / ME3EM4b	/-/	3.7	14.31	−6.15	1
Shi et al. 2009	SNPPw	*qSN.C8-2*	C08	IGF3369a / MR64	28.8-31.4	5.5	5.1	0.73	1
Shi et al. 2009	SNPPw	*qSN.C8-3*	C08	sN11670a / IGF2529z	31.8-33.6	5.3-7.4	5.4-7.8	+	2
Shi et al. 2009	SNPPw	*qSN.C8-4*	C08	IGF2529z / pW123cE	33.6-35.3	4.6	4.3	0.39	1
Shi et al. 2009	SNPPw	*qSN.C8-1*	C08	S15M04-2-100 / Ol12G04	/-/	3.4-5.4	5.1-5.2	-	2

Shi et al. 2009	SNPPw	*qSN.C9-2*	C09	E2HM34-260 / Na12G04	33.2-34.0	2.6-6.4	4.3-8.6	-	6
Zhang et al. 2011	SNPPm	*qSS.N19*	C09	EA13MC03-150 / Na10D07	39.4-/	2.5	6.11	2.07	1
Shi et al. 2009	SNPPw	*qSN.C9-3*	C09	IGF5193b / CB10288	39.0-40.0	2.3-5.9	3.2-8.1	-	6
Zhang et al. 2012	SNPPm	*qSS.C9*	C09	SRC9-022 / SA09MC04	44.6-46.0	22.5	57	5.40	3
Radoev et al. 2008	SNPPm	*S/Sil_N19*	C09	CB10345 / Na10B11b	11.1-12.4	6.1	3.6	0.99	1
Shi et al. 2009	SNPPw	*qSN.C9-1*	C09	SA30 / S15M04-2-92	/-/	2.7-5.8	3.4-7.9	-	4
Cai et al. 2014	SNPPm	/	/	EA05MC08_8	/-/	3.07	3.96	0.8	1
Cai et al. 2014	SNPPm	/	/	EA06MC09_10	/-/	3.45	4.44	1.4	1
Cai et al. 2014	SNPPm	/	/	EA06MG08_9	/-/	3.02	3.85	2.1	1
Cai et al. 2014	SNPPm	/	/	EA06MG10_5	/-/	3.63	4.7	1.5	1
Cai et al. 2014	SNPPm	/	/	EA08MC10_5	/-/	3.45	4.4	1.1	1

Table S8 Information of the putative single-locus SSR markers chosen from the target major QTL region for use in regional association analysis.

Marker name	Scaffold start (bp)	Scaffold end (bp)	Forward_primer (5'-3')	Reverse_primer (5'-3')	Tm (°C) forward	Tm (°C) reverse	Product size	Polymorphism & single-locus
BrSF47-179	373,862	373,877	TTGGATGAGCGTAGTTGTGG	TTTTGGATGCCCTAAGCAAT	60	60	152	√
BrSF47-175	371,407	371,420	TCCATCAATAATGAACATGCG	ATATCCGCGTAATAATTTAGCAA	59	57	264	
BrSF47-163	342,625	342,646	AACGCGAGCAATTAGGAGAA	CAAGCCCACCTCAATAATCG	60	60	250	√
BrSF47-127	268,155	268,170	TTTGAATACAATATTATCCATTGTTCA	TGCTGATTGTCACATTCTCCAT	58	61	218	
BrSF47-78	171,249	171,262	TTCCTAAACATTCTTTAATTTTGTGC	TTTTACGTGACGTGGTGGAG	59	60	261	√
BrSF47-71	156,414	156,427	CAAATATTTCCTGGCGTCGT	CCGGATCAGTCGATAGGTCT	60	59	131	
BrSF47-54	114,543	114,686	GAGTTGAAACCCCAAGCAAG	GCGAAACAGAGGCAATAAGG	60	60	219	√
BrSF47-23	44,651	44,677	GATCTATAAGGGCGCTGCAA	ATTGGCCCAAAATCTCTTGA	60	60	235	√
BrSF47-10	25,879	25,898	ATCAATTTTTCCGAGACCCC	AAGCTGGTGAACTCGTTTCG	60	60	264	√
BrSF46-1	1,253	1,267	TCCCCATTAAGTAGGAGAGGC	CGGTGAGGAAATCCATGTTT	60	60	185	
BrSF46-3	8,690	8,703	TTGTTCTCAGAAACCATGAGAGA	TGGCCAAACATTATGGTCATT	59	60	151	
BrSF46-7	15,318	15,330	CTGGTTCGAGACAGAAACACA	TTCCGATTTGGAAACACAAA	59	59	276	√
BrSF46-15	33,566	33,581	GTGGGGAAGTACCGAGCTTT	CCACATCATTTGTTTTGCCA	60	60	267	
BrSF46-20	37,841	37,858	AAGGTCAGGATTATGGGGCT	AAAACTTTGCATGATCGGCT	60	60	212	
BrSF46-22	44,114	44,245	TCGTGTAAGTGAGAGGAGAGTAAAGA	AGCTGGTGCAGTATGTCAGC	60	59	229	
BrSF46-27	53,797	53,813	ATCTTGACATTTCGAGGCGT	TTCATGAATCCATTAGGCTCC	60	59	252	
BrSF46-28	54,246	54,257	CGTGGCGACATGTCTGAATA	AGGTAGGGAGGGGATTTGAA	61	60	171	√
BrSF46-49	94,698	94,709	TGCATTTCATTTTCATGCCA	AATGATTGAAATACGGTGGGA	61	59	275	√
BrSF46-75	145,365	145,382	GTGACAGCTGTTGGCTGTTG	CTCATGCACGCTCTCCCTAT	61	60	247	
BrSF46-78	148,450	148,574	TGCATAATCACCTAATACTAGTTTGC	GCACAGCCAACGTTTTGAA	58	61	280	√
BrSF46-117	210,176	210,224	CCATTCTGCCTCTCTCCTTG	GGCCTTATTGGAAGCATGAA	60	60	184	√
BrSF46-136	246,805	246,818	GTCAGGAGGTAAGAGCGGTG	ATCCGATTCTCTTGCCATCA	60	61	166	√
BrSF46-141	249,690	249,745	GCTAGTCAAGCTTCGTCGGT	TCGATATTCCAGAAGGCAGG	60	60	245	
BrSF46-142	249,860	249,943	TTTGATAGCCTGCCTTCTGG	TCCGAGGATTCGTTGGTTAG	60	60	237	
BrSF46-160	291,338	291,352	CAGATCGTTGCCACATCAGT	ACTCCCAAGCCTCTGTGCTA	60	60	217	
BrSF46-161	291,761	291,776	CCCAAAAATAATACTCCCTCCG	ATCTGTTACACCGCAATCCG	61	61	276	√

BrSF46-162	293,215	293,234	TCTTCACTCCTACCCCTCCC	GAAAAGCGAAGACGGAAATG	60	60	208	
BrSF46-166	299,780	299,793	TTACGTTTTTGGGCTTGGTC	TGGCTTGACCCCAGTCTTTA	60	61	164	
BrSF46-167	303,352	303,389	TCGGCTAGAATGGCTACAAGA	TGCCCATTCGTGTATGTTTG	60	60	273	√
BrSF46-176	315,522	315,543	ATCACCATTGTGTTATTTCTTCAG	GGATCAAATGGATTTAGCGG	57	59	256	
BrSF46-177	316,223	316,240	AGCTGGTACATGATTGCGTG	CAACTCTGCCATTCACATGC	60	60	268	√
BrSF46-186	324,357	324,370	GCCAAAGTGCATCCCATCTA	CCTTTGGAAAGTCGAGAGACC	61	60	161	√
BrSF46-204	345,157	345,170	TCCAACCGATTCAACCAAAT	TGTATGCCAAAATAACATTCATCT	60	58	206	
BrSF46-211	353,809	353,823	GCCTTTAGTTTGCAACCATTC	AAAACACTTTGCGATTTGGG	59	60	251	
BrSF46-213	358,746	358,760	GGAGATAGCTTTACGGTGCG	TCATGGGTCCACACATACATTT	60	60	220	
BrSF46-220	371,686	371,756	CGCTGTCTCTTCGCTTCTCT	ATCGACGCCAAATCTAAACC	60	59	246	
BrSF46-223	385,268	385,303	GGGTTTAGAGATAAAGAATGGAGTTT	TGAAAATAATGAACAATTCTCTCAAA	59	58	255	
BrSF46-230	407,280	407,354	TGAGGTACAAACTTCACAATCTTACA	GTACCATCGGATTTTGGTGG	59	60	258	
BrSF46-232	407,786	407,799	TTGAGAAGAATTAGAAGAAAAGACGA	CAGCTATTACTCCTTCCGTTTCA	59	60	278	
BrSF46-235	416,782	416,893	TGAATTGGGAACAGCTAGGG	GGTGAGTTCGAGTCGGGTTA	60	60	209	
BrSF46-238	422,781	422,806	TTGCGAGATTATTTTTAGATATGTGC	GAAAGCGTGAATCAAAAACG	60	58	146	√
BrSF46-240	429,763	429,776	TGAAAAAGGAGTTCAGAGTAAAACAA	TGTGAAAGGATTGAACATTGC	60	59	280	
BrSF46-256	459,725	459,850	TGAATGCAACCACCTCACAT	GTCTTGAGGAGCAGCGAAAT	60	60	236	
BrSF46-259	467,296	467,321	GGGCAGGCTAACCCCTAGTA	GAAGACGACGACATCGGTTTA	60	60	276	√
BrSF46-265	477,163	477,177	TGTGAGGTGACCATTGTTTTTA	CGTGGTCAATGAAACACACA	58	59	249	√
BrSF46-267	483,948	484,041	TATTATCTTCTCCCGGCGAC	AAGTATTAAAGGACGGCGGA	59	59	278	
BrSF46-269	485,062	485,079	TCAATGTTTCCCTCTTCTTTGA	CCCAAGGATTTAGGATTTAGGG	59	60	256	
BrSF46-276	490,198	490,213	AACCATGTTACACCGTTCCG	CAGTTTTGAATTTTATTTTGGGG	61	59	264	
BrSF46-286	508,142	508,159	TGCTCTGCATAATCACATTTCA	ATTTGATTGTCCGCTTCATT	59	57	260	
BrSF46-288	516,227	516,242	ACTGGTCAAGTGGTCCTTGG	CACTGATGATCGTTGCTGCT	60	60	223	
BrSF46-291	523,348	523,362	CCACGTCTGAGGTGAGGTTT	CTTTTCTTCAATGCCACCGT	60	60	183	√
BrSF46-292	523,613	523,640	ACGGTGGCATTGAAGAAAAG	TTCTCTCTCCCACACACAACA	60	59	237	
BrSF46-306	557,446	557,469	ATGATCATGTGGACCGTTGA	TGATTGTTATTCCCAACTTTTTAGG	60	60	233	√
BrSF46-314	573,510	573,524	TTGGGCCTCACTGATAGTCC	TCAAAACAAAGCCCAAAACC	60	60	237	
BrSF46-319	578,293	578,349	TTTTTGGGCTTGGTCTACGA	CCTCTGTCTTCGGAGCAAGT	61	60	248	
BrSF46-325	589,391	589,420	TTTCAAGCTGACGTGGACAG	AACATTGGTGCTTGATTGTGA	60	59	202	

BrSF46-353	651,753	651,776	GCCACTGGGTCAAAATGTCT	TTGGTGAGACGACTTGTGATTT	60	60	251	√
BrSF46-367	680,283	680,308	TCAAGGTAAATTCCCATCCA	CATTGTGCTTGGAGATGGTG	58	60	224	
BrSF46-383	706,218	706,260	CCATTTTTCATCTTACCTTATTTTGC	CCTAGCCAAAACCATTCTATGT	60	57	280	√
BrSF46-386	712,186	712,220	TTGGTAAGAAAATGAATCTTGGG	GCGGGTCAAAGGCTAGTTCT	59	61	263	
BrSF46-407	765,285	765,304	TGGGACAGAAATCAAATCCC	GTGGCGGAGAAAAGAAACTG	60	60	143	√
BrSF46-415	779,110	779,134	TTCTTCCTCTCGGCTTCGTA	CATGCATTTTCTGCTTTTGC	60	59	239	
BrSF46-432	810,447	810,528	TTGGGGTTGTTTCATGTTTG	CGGTAGTCCACGGGAAATTA	59	60	191	√
BrSF46-439	828,748	828,779	CCAGTCGTTCTCTCTGAGGC	GGGCGTATGGATTTGCTAGA	60	60	277	
BrSF46-454	864,942	865,005	CGGTTCAGTTACGGCAAAGT	CACCTTTCGCTTTTGTTCTTTT	60	60	280	√
BrSF46-467	903,409	903,441	TGCTCTGCACAAACTCCATC	TGGAAGCTTGGAGGAAGAAA	60	60	272	√
BrSF46-485	945,845	945,866	TCCCCCAAAATAAATATCCACA	GGCCTCAGAGGAGACAAGAA	60	60	279	
BrSF46-509	976,750	976,783	AGCCACTAGGAGCAAGATGA	TTTGTTTGTCTTTTTGGGGG	58	60	237	√
BrSF46-533	1,019,716	1,019,824	TGACCAATTACAGAGCGTCG	GGCCTTAACGGTTCCTCAAT	60	60	244	
BrSF46-541	1,041,232	1,041,258	CCAACAGTGGAGCCTAAAGC	CTCGTTTGTCGCTGGTGTTA	60	60	232	
BrSF46-551	1,050,185	1,050,292	TTCACGTACGTACTCGATTTCTTC	TGAGAAAGGGAGTTTCCTGG	60	59	241	√
BrSF46-552	1,050,924	1,050,943	CCTTCTCCGCGAAGTATCAG	GCAGAAACAGAGGCAAGACC	60	60	276	
BrSF46-554	1,053,021	1,053,042	GCGCTCTATTTTGGTTGCTC	GAGGCAAACCACTTGGAAAT	60	59	222	
BrSF46-557	1,061,135	1,061,154	GCGAGTAACTTTGGGCAGAC	GAGTTGCAACCACAACTTGG	60	59	248	
BrSF46-574	1,099,671	1,099,697	TGAGTGTATGTGTTGATGTGGC	ACAGAAAAGTTTTGGTTATTGCTT	59	58	256	√
BrSF46-576	1,103,028	1,103,049	CCCGCTATGCAGGCTTAAAT	TGTTGTTTTCTTTTGTAACTGGGA	61	60	272	
BrSF46-583	1,118,083	1,118,123	CACAGACCAGGGATGTAAGGA	CATTTAGCAAGCGATGACCA	60	60	213	
BrSF46-586	1,121,623	1,121,646	GTGAAGCCCATCGTCATCTT	CGAAGACTTTGGAGGAGCAG	60	60	239	√
BrSF46-610	1,185,437	1,185,540	TTTTTGTTTGTTTCTCACTCAACA	CATCCATTAATTGGGAACTATGA	59	58	164	
BrSF46-614	1,198,103	1,198,124	TGTTGTTGTACCCGTAATGAAGA	TTTTTCATGTTTCATCTTTCTCTCT	59	58	270	
BrSF46-619	1,204,669	1,204,692	AAAAGATTTGTTTGGAGTTGGAA	TTGAGGTTTGGTTTGGAAGAA	59	60	215	
BrSF46-626	1,220,435	1,220,547	TTTTGAAGTTACACCAATTTTCAGA	ACAAAGCATGCCATCATCAA	59	60	261	√
BrSF46-648	1,256,758	1,256,783	GACTTCACTGTTCCCACGGT	CCCTCTCACCCATTTCTCAA	60	60	277	
BrSF46-661	1,287,088	1,287,111	CAACGAATTTTAAAGACGCAAC	TCTTTAAATCGTCTTTTCCATGC	59	60	211	√
BrSF46-673	1,309,908	1,309,980	TAAGGGCGTTCACAAACGAT	AGAAGCTCATCTCTGATGCGT	61	59	234	
BrSF46-687	1,345,821	1,345,891	TATTTTCCCCCGTTTCACAA	TTTTGCTCGCTTTCGATTTT	60	60	203	√

BrSF46-700	1,379,320	1,379,433	AATTTGGGTTAAAACCCTTAAACT	CTCTTCCATAGGTCCGGGAT	58	60	206	√
BrSF46-711	1,411,875	1,411,910	AAAGTAGGAATGAAAAATTTTGGG	CAAAGCTGAAAGCACGAGTT	59	58	235	
BrSF46-744	1,459,489	1,459,620	AACAAAATTTTCATTGTGGGA	CAAAATCATATCAGTCGAACGTC	57	59	246	
BrSF46-791	1,558,134	1,558,266	TGGAAAATTTAGCAAAGTATAGCAAA	TTTTTGCGACCGATAAAATTAAA	60	60	248	
BrSF46-796	1,562,809	1,562,905	GCAAAAGCAACAAGAGGGAG	CTCGTTTCGGTTTCAATTCC	60	60	155	
BrSF46-799	1,564,189	1,564,210	AACCGCTTTATTTGGCAATG	GCGAGGGACAGAGTGATTTC	60	60	182	
BrSF46-812	1,595,044	1,595,075	TGCATTTAGGGCCCAGTATT	TGGCTTCGTCGAGAAAATCT	59	60	256	
BrSF46-827	1,622,834	1,622,853	TGTCACGCGTCATTCATAGA	TGCATGATTTTTGACAGGCT	59	59	268	
BrSF46-836	1,639,161	1,639,251	TCGATCTACAGGAATCTTGAAAAA	CCAAAACAAGAAAGGTTGGTT	59	58	280	
BrSF46-837	1,641,718	1,641,827	CTGCGATGGAATTGGAATCT	AGAGTAGCTAGCATCACCAAAAA	60	57	245	
BrSF46-854	1,674,049	1,674,070	GGTGGCAGAACCGTAAACAT	TCTGATGGGTCTTTCGTGTG	60	60	257	
BrSF46-880	1,731,797	1,731,856	GCAGCTTCCACAACAAACAA	CGAAGTGCGTATTCACAAGC	60	59	137	
BrSF46-887	1,753,395	1,753,453	GCGATCAAAGGATTCAAAGC	CCTGTCTCCAACAAGTTCCC	60	60	225	
BrSF46-895	1,774,150	1,774,183	CCTCTTCTTCTTGGTCTCGC	AGGGCATTGTTAAGTCCCTG	59	59	246	
BrSF46-910	1,801,928	1,801,997	GGCTAGTGAAGTAAACGGCG	AATCTCGGACCGGAGACAT	60	59	280	
BrSF46-913	1,817,946	1,818,030	TGCCAAACCAAACACTTTCA	GGGAACAGTGGGAGTAGCAA	60	60	219	
BrSF46-920	1,829,662	1,829,694	CTGGTGTAACCTGGTGCAAA	CCCCCTTAATGAGTTTGTGC	60	59	238	
